# Cognitive functioning in adolescents with severe obesity undergoing bariatric surgery or intensive non-surgical treatment in Sweden (AMOS2): a multicentre, open-label, randomised controlled trial

**DOI:** 10.1016/j.eclinm.2024.102505

**Published:** 2024-02-27

**Authors:** Kajsa Järvholm, Eva Gronowitz, Annika Janson, Markku Peltonen, Lovisa Sjögren, Andrew J. Beamish, Jovanna Dahlgren, Johan Mårtensson, Torsten Olbers

**Affiliations:** a Department of Psychology, Lund University, Lund, Sweden; b Childhood Obesity Unit, Skåne University Hospital, Malmö, Sweden; c Department of Paediatrics, Institute of Clinical Sciences, Sahlgrenska Academy, University of Gothenburg, Gothenburg, Sweden; d National Childhood Obesity Centre, Karolinska University Hospital, Sweden; e Department of Women's and Children's Health, Karolinska Institutet, Stockholm, Sweden; f Finnish Institute for Health and Welfare, Helsinki, Finland; g Region Västra Götaland, Sahlgrenska University Hospital, Regional Obesity Center, Gothenburg, Sweden; h Welsh Institute of Metabolic and Obesity Surgery, Morriston Hospital, Swansea, United Kingdom; i Swansea University Medical School, Swansea University, Swansea, United Kingdom; j Department of Clinical Sciences Lund, Lund University, Lund, Sweden; k Department of Biomedical and Clinical Sciences and Wallenberg Center for Molecular Medicine, Linköping University, Linköping, Sweden

**Keywords:** Obesity, Adolescents, Bariatric surgery, Cognitive functioning

## Abstract

**Background:**

Severe obesity during childhood is associated with cognitive deficits. Studies in adults have suggested improvements in executive functioning and memory after bariatric surgery. Our aim was to explore changes in cognitive function in adolescents over two years after bariatric surgery or intensive non-surgical treatment.

**Methods:**

The Adolescent Morbid Obesity Surgery 2 (AMOS2) is a multicentre, open-label, randomised controlled trial in which adolescents (aged 13–16 years) with severe obesity (defined as body mass index (BMI) ≥35 kg/m^2^) at three specialised obesity centres in Sweden, were randomly assigned to receive bariatric surgery or intensive non-surgical treatment. Herein we report the results of the prespecified exploratory endpoint of change in cognitive functioning. Inclusion in AMOS2 required Tanner pubertal stage ≥3, previous participation in lifestyle obesity treatment for at least one year, and passed assessment form a paediatrician and a paediatric psychologist. Adolescents with severe intellectual disability or other severe, pervasive developmental disorder were excluded. Participants underwent baseline assessment of general intellectual ability, executive functioning, and memory before randomisation. Tests were administrated by clinical psychologists and repeated at one- and two-year follow-up timepoints. Differences in means between groups during follow-up are provided with confidence intervals. The trial is registered at ClinicalTrials.gov, NCT02378259.

**Findings:**

Between October 28 2015 and June 7 2017, 46 adolescents (74% girls), with a mean age of 15.8 (±0.92) years and a mean BMI of 42.8 (±5.4) kg/m^2^, were included and randomised (23 to bariatric surgery and 23 to intensive non-surgical treatment). At baseline 23/46 (50%) of the adolescents had general intellectual functioning classified as borderline or below. For 15/18 (83%) aspects of cognitive functioning, no significant differences in change over two years were identified between groups; Immediate (average difference during follow-up 1.0 [95% CI: −2.6 to 4.6]) and Delayed (0.5 [95% CI: −0.6 to 1.6]) Verbal Recall, Category Fluency (1.1 [95% CI: −1.6 to 3.8]) and Switching (1.5 [95% CI: −0.0 to 2.9]), Number (−6.0 [95% CI: −12.3 to 0.3]) and Letter (0.1 [95% CI: −5.2 to 5.3]) Sequencing, Number-Letter Switching (−10.3 [95% CI: −26.4 to 5.8]), Motor Speed (−8.3 [95% CI: −17.5 to 0.9]), Colour Naming (−1.9 [95% CI: −4.2 to 0.3]), Inhibition (−3.6 [95% CI: −9.6 to 2.5]), Inhibition Switching (−6.7 [95% CI: −15.3 to 1.9]), Mazes (−0.5 [95% CI: −4.9 to 3.9]), Digit Span Forward (0.1 [95% CI: −0.6 to 0.9 ]) and Backward (0.6 [95% CI: −0.4 to 1.6 ]), and Estimated IQ (0.4 [95% CI: −3.9 to 4.8]; all p > 0.05). Three sub-tests assessing fundamental cognitive skills improved more over two years in operated adolescents than in intensive non-surgical treatment; Letter Fluency (average difference during follow-up 3.8 [95% CI: 0.1–7.5]; p = 0.046), Visual Scanning (−6.5 [95% CI: −11.6 to −1.5]; p = 0.011), and Word Reading (−1.9 [95% CI: −3.3 to −0.4]; p = 0.011).

**Interpretation:**

In contrast to non-randomised studies in adults, we could not demonstrate an association of bariatric surgery and its accompanying significant weight loss with overall greater improvement in executive functions and memory in adolescents over two years compared with a non-surgical group without weight loss. However, lack of statistical power is a potential limitation. The clinical relevance of greater improvements in basic cognitive skills needs to be explored.

**Funding:**

Sweden's innovation agency (VINNOVA), 10.13039/501100004359Swedish Research Council, Joanna Cocozza foundation for paediatric research, The Skane University Hospital Psychology 10.13039/100006190Research and Development Grant, Tore Nilsson’s Foundation, SUS Foundations and Donations, and Mary von Sydow’s Foundation.

**Research in context**

**Evidence before this study**

PubMed was searched for full-text clinical reports and reviews, not limited to English language, published before 1st January 2015 using the MeSH term “bariatric surgery” and “cognitive function∗” in all fields. The search returned 27 articles, of which 17 were deemed relevant. No relevant review was found.

Of the 17 articles, 13 emanated from the same US observational multicentre study of adults undergoing bariatric surgery. A follow-up of 50 middle-aged adults demonstrated improvements in memory and executive functions three years after bariatric surgery, a finding supporting the notion that bariatric surgery may, at least partially, reverse cognitive deficits related to obesity.

**Added value of this study**

In this randomised trial of 46 adolescents with severe obesity aged 13–16 years, we could not demonstrate any clear overall difference between surgically and non-surgically treated participants over two years in tests assessing memory or executive function. However, operated adolescents demonstrated statistically greater improvements in three subtests assessing fundamental cognitive skills, such as visual scanning.

**Implications of all the available evidence**

Studies in adults have indicated improved memory and executive functioning after bariatric surgery. The present study contributes the first randomised data on cognitive functioning after bariatric surgery and could not show that bariatric surgery is superior to intensive non-surgical treatment in improving memory or executive function in adolescents across two years. The clinical relevance of greater improvement in fundamental cognitive skills after surgery is unclear, and the findings need to be confirmed in future studies.

## Introduction

Bariatric surgery is the most effective treatment for severe obesity, leading to sustainable weight-loss and reduced morbidity and mortality.^1^ Its use in adolescents with severe obesity is increasing.^2^

Even during childhood, obesity is associated with cognitive deficits,^3^ and it has been suggested that particularly severe obesity is associated with a reduced likelihood of adolescents reaching their full cognitive potential.^4^ The cognitive deficits seen are especially related to executive functions, i.e. the ability to plan, to regulate and inhibit impulses, and sequencing.^3^^,^^5^ There is support for a bidirectional relationship between obesity and cognitive deficits,^5^ but no causal mechanism has been demonstrated. Obesity-related comorbidities, such as obstructive sleep apnoea and type 2 diabetes, are associated with a heightened risk of impaired cognitive functioning. Still, the relationship between obesity and cognitive deficits appears to transcend that attributable to its comorbidities alone.^3^^,^^6^

Prospective studies in adults have suggested improvements in several cognitive domains, sustained at least until three years after bariatric surgery.^7^^,^^8^ Thus, it has been hypothesised that cognitive deficits associated with obesity could have a degree of reversibility in response to weight loss.^9^ However, it has been suggested that only immediate verbal memory and delayed memory show significantly greater improvement among patients undergoing bariatric surgery when including non-operated comparators.^8^

So far, few studies have assessed cognitive functioning and outcomes in adolescents undergoing bariatric surgery. A study using functional MRI suggested normalisation of brain activity in areas associated with executive functioning after adolescent bariatric surgery.^10^ However, there were no significant improvements in verbal memory and executive functioning in adolescent and young adult females (n = 21; age 13–24 years) 12 months after bariatric surgery, in comparison to a group without obesity.^11^

In conclusion, findings are inconsistent regarding the effect of bariatric surgery and related weight loss on cognition, especially in adolescents. The present study reports baseline cognitive functioning in adolescents with severe obesity included in a randomised controlled trial and uses validated assessments from clinical practice to explore the changes in cognitive functioning of these participants over two years after bariatric surgery or intensive non-surgical treatment.

## Methods

### Study design and participants

The Swedish Adolescent Morbid Obesity Surgery 2 (AMOS2) study is a randomised open-label multicentre trial assessing outcomes in adolescents with severe obesity after bariatric surgery (predominantly Roux-en-Y gastric bypass [RYGB]) and intensive non-surgical treatment with an initial eight-week low calorie diet. Adolescents were included from childhood obesity clinics at three Swedish university hospitals (Stockholm, Gothenburg, and Malmö). All adolescents randomised to bariatric surgery were operated in Gothenburg. The primary endpoint in AMOS2 was change in body mass index (BMI) over two years. Participant recruitment and study design have been reported elsewhere.^12^ Reporting adhered to the CONSORT guidelines for randomised trials.

AMOS2 was approved by the ethical review board in Gothenburg in January 2014 (registration number 578–13). The inclusion of cognitive tests in AMOS2 was approved in an addendum to the original ethical approval in June 2015.

Guardians and participants ≥15 years signed an informed consent at study inclusion. Participants <15 years provided signed assent. Inclusion criteria in AMOS2 were age 13–16 years (in original protocol 13–15 years, adjusted due to slow recruitment), BMI ≥35 kg/m^2^, having participated in a comprehensive obesity treatment at a paediatric unit for at least one year, and a preparedness to undergo bariatric surgery.^12^ Exclusion criteria included monogenic or syndromic obesity, obesity secondary to a brain injury, and severe intellectual disability or other severe, pervasive developmental disorder (a full list of inclusion and exclusion criteria is presented in Supplementary Appendix p.4). Adolescents with milder forms of intellectual disabilities were not excluded if they were able to understand and assent (<15 years) or consent (≥15 years) to the study protocol. Inclusion in AMOS2 stopped when 50 participants were included.

### Randomisation and masking

Participants were randomly assigned 1:1 to bariatric surgery or intensive non-surgical treatment using computerised randomisation, with stratification for sex and recruitment site. The randomisation was centralised to a research laboratory, not otherwise involved in the study, and the allocation was concealed for participants, caregivers, and research staff until the end of the day of inclusion in the study. Allocation was thereafter unblinded as group allocation was impossible to conceal (surgical vs non-surgical treatment).

### Procedures

Cognitive functioning was assessed with validated tests frequently used in clinical assessment. All tests were paper-and-pencil administrated by clinical psychologists. The assessment was done at the same day as the inclusion in AMOS2 or on a day just before inclusion. For some participants the baseline assessment was done on two different days due to the high response burden in the study. The cognitive assessment was repeated at the follow-up encounters one and two years after inclusion.

For assessment of general cognitive functioning, four subtests (Verbal Comprehension, Perceptual Reasoning, Working Memory, and Processing Speed; see Table 1) from the Wechsler Intelligence Scale for Children—Fourth Edition (WISC-IV) or Wechsler Adult Intelligence Scale—Fourth Edition (WAIS-IV) were used to assess an estimated full intelligence quotient (IQ). The four subtests represent a core test from each primary index in WISC-IV and WAIS-IV.

**Table 1:** Description of the subtests used in the cognitive assessment.

Test	Subtests	Description
WISC-IV/WAIS-IV	Vocabulary	A core Verbal Comprehension subtest assessing several cognitive factors such as verbal comprehension, language development, and crystallised knowledge. No speed component.
	Matrix Reasoning	A core Perceptual Reasoning subtest assessing broad visual intelligence. No speed component.
	Digit Span	A core Working Memory test assessing auditory short-term memory and auditory sequential processing. Both WISC-IV and WAIS-IV contain Digit Span Forward (repeating digits as given) and Digit Span Backward (repeating digits in reverse order). WAIS-IV also contains Digit Span Sequencing, where the test taker is asked to repeat the digits in an ascending numerical sequence. A higher raw score indicates better performance for all conditions.
	Coding	A core Processing Speed test assessing visual motor-coordination, visual short-term memory, attention, and concentration.
D-KEFS	Verbal Fluency	Assesses fluent productivity in the verbal domain in three conditions: Letter Fluency^a^ Category Fluency^a^ Category Switching^b^ A higher raw score indicates better performance for all conditions.
	Trail Making Test	Assesses the flexibility of thinking on a visual-motor task in five conditions: Visual Scanning^a^ Number Sequencing^a^ Letter Sequencing^a^ Number-Letter Switching^b^ Motor Speed^a^ A lower raw score indicates better performance for all conditions.
	Colour-Word Interference Test	Assesses verbal inhibition of an overlearned verbal response as well as flexibility in four conditions. Based on the Stroop procedure. Colour Naming^a^ Word Reading^a^ Inhibition^b^ Inhibition/Switching^b^ A lower raw score indicates better performance for all conditions.
WISC-IV integrated	Elithorn Mazes	A non-verbal measure of executive functioning that requires self-monitoring, planning, and inhibition of impulses. A higher raw score indicates better performance.
NEPSY-II	Verbal Memory	Assesses direct and delayed verbal recall using a list of 15 words. A higher raw score indicates better performance.

WISC, Wechsler Intelligence Scale for Children; WAIS, Wechsler Adult Intelligence Scale; D-KEFS, Delis-Kaplan Executive Function System; NEPSY-II, A Developmental NEuroPSYchological Assessment.

a A sub-test from D-KEFS assessing fundamental cognitive skills.

b A sub-test from D-KEFS assessing executive functioning.

WISC-IV can be used to assess general cognitive ability in children and adolescents aged 6:0–16:11 (years:months), and WAIS-IV can be used for assessment in adolescents and adults aged 16:0–90:11. Several subtests are analogous in WISC-IV and WAIS-IV and the appropriate test for each participant was chosen according to their age. Participants aged 16 years at baseline completed the WAIS-IV assessment, as it enabled assessment with the same instrument over follow-up. All assessments with a participant aged below 16 years at the time of completion used WISC-IV.

Raw scores on each subtest in WISC-IV and WAIS-IV are converted to scaled scores (mean of 10 and a standard deviation [SD] of 3; range 1–19) using age-specific norms. In WISC-IV norm values are given for every age group in three-month intervals e.g., 16-0-0 through 16-3-30 (years-months-days), whereas WAIS-IV norm values for adolescents (16–19 years) are given in two-year intervals e.g., 16-0-0 through 17-11-30. Scaled scores 1–4 are interpreted as far below average, 5–7 as below average, 8–12 as average, 13–15 above average, and 16–19 as superior.^13^

The sum of the scaled scores from the 4 subtests in either WISC-IV or WAIS-IV was converted to an estimated full scale IQ using tables published by Sattler and colleagues.^13^^,^^14^ In population samples mean IQ is 100 (SD ± 15) and in population samples general cognitive functioning is supposed to be normally distributed. IQ scores can be classified as intellectual disability (≤69), borderline intellectual functioning range (70–84), normal range (85–115), and above-average range (>115).

Executive functioning was assessed with three subtests from Delis-Kaplan Executive Function System (D-KEFS), part of a subtest from NEPSY-II (A Developmental NEuroPSYchological Assessment), and one subtest from WISC-Integrated, see Table 1 for a detailed description of the subtests. The subtest Digit Span from the WISC-IV and WAIS-IV was also considered a measure of executive functioning as it assesses working memory. In D-KEFS, some conditions measure fundamental cognitive skills (e.g., attention, perception, and language), and other conditions measure executive functioning (e.g., inhibition, planning and cognitive flexibility). Raw scores on D-KEFS were transformed to scaled scores using age-specific norms.

Sex data were retrieved from medical records and thereafter confirmed with the adolescent's self-identification.

### Outcomes

Changes in cognitive functioning (verbal memory, executive functioning, fundamental cognitive skills, and estimated IQ) over two years are reported, which were prespecified exploratory endpoints in the AMOS2-trial.

### Statistical analysis

Mean values with SD and proportions with frequency counts were used to describe baseline characteristics of the participants. Longitudinal changes were analysed using mixed-effects regression models. The observations were considered nested within the individuals, and the statistical tests and CIs were calculated taking the repeated measurements into account. For individuals who dropped out of the study, we assumed missing data were “missing at random” (i.e. missing data are only dependent on the participant's observed data), and all the observed data were used in the analyses. Thus, all the baseline and 1- and 2-year follow-up data was used to estimate the differences between the treatment groups. The assumptions of the models were evaluated through analysis of residuals (independence of residuals, equal variance).

The effect of treatment is expressed as the mean difference between the groups during follow-up, adjusted for respective variables baseline and for stratification variables (sex and study centre), with 95% confidence intervals. No correction for multiple testing was done, as analyses are considered explorative. All analyses on cognitive functioning variables were prespecified, and we report the results of them all here.

The primary analysis was based on intention to treat. All participants were analysed in the treatment group they were originally assigned to, even after crossing over to the other treatment arm. A sensitivity analysis was also done based on as treated (per-protocol). In this analysis, persons who crossed-over to the other treatment arm were censored at the corresponding time-point, and their data after conversion was not used in the estimation of the treatment effect. In addition, as 3/23 patients (13%) in the surgery group and 8/23 patients (35%) in the intensive non-surgical treatment group dropped out before the 2-year follow-up, a sensitivity analysis was also done with multiple imputation of the missing outcome data. Imputation was based on regression using sex, age, BMI, study centre, treatment group, and all the existing data of respective cognitive function variable, as predictors of missing data. The number of imputations (complete datasets with imputed values) were set to 30.

All statistical tests were two-tailed, and p-values less than 0.05 were considered statistically significant. Statistical analyses were carried out using Stata, version 15.1 (StataCorp LP). This study is registered at ClinicalTrials.gov, NCT02378259.

### Role of the funding source

The funders of the study had no role in study design, data collection, data analysis, data interpretation, writing of the report, or the decision to submit the paper for publication. KJ and MP had full access to and verify the underlying study data. All authors had a final responsibility for the decision to submit for publication.

## Results

### Participant characteristics and treatment

**Fig. 1:**
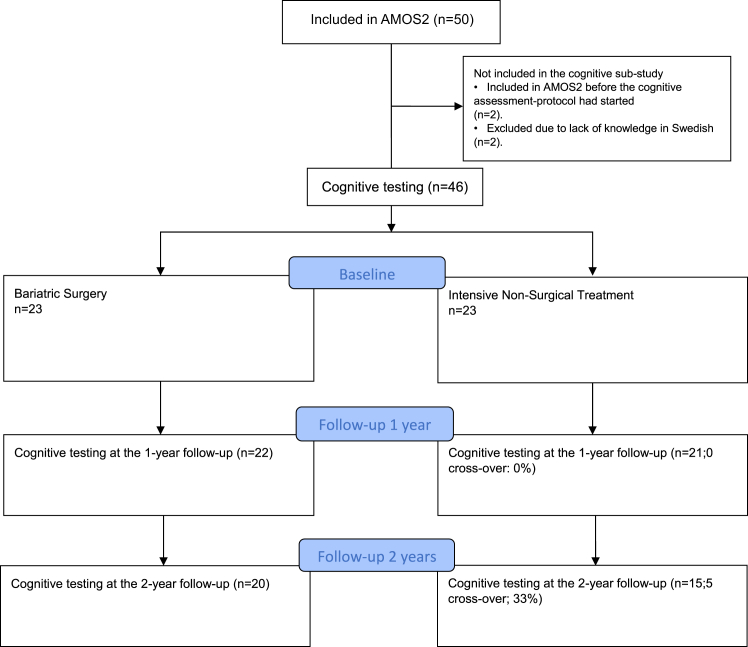
Trial profile. AMOS, Adolescent Morbid Obesity Surgery. Flow chart showing the number of participants at each assessment point. No participant randomised to intensive non-surgical treatment crossed over to bariatric surgery between baseline and the one-year follow-up. Five participants randomised to intensive non-surgical treatment crossed over to bariatric surgery after the one-year follow-up, but before the two-year follow-up.

Out of 50 participants included in AMOS2, 46 (92%) adolescents participated in this study on cognitive functioning. The 46 participants (74% girls) were included in AMOS2 between October 28, 2015, and June 7, 2017. Two participants were enrolled before the cognitive testing was initiated in AMOS2, and two others were excluded as their knowledge of the Swedish language was insufficient (Fig. 1).

The primary and the secondary two-year outcomes in AMOS2, except cognitive functioning, have been reported previously. Bariatric surgery was superior to intensive non-surgical treatment in achieving weight loss, improvements in cardiovascular risk factors, physical quality of life, and control over eating.^15^

All participants underwent cognitive assessment before being randomised to either bariatric surgery or intensive non-surgical treatment in the AMOS2 study. The mean age of the participants was 15.82 (±0.92; range 13.30–16.97) years, and mean BMI was 42.81 (±5.36) kg/m^2^ at baseline. Baseline characteristics for each group are reported in Table 2.

**Table 2:** Patient characteristics and cognitive functioning at baseline.

	Surgery (n = 23)^a^ n (%) or mean (SD)	Intensive non-surgical treatment (n = 23)^a^ n (%) or mean (SD)
Patient characteristics		
Female	17 (74%)	17 (74%)
Male	6 (26%)	6 (26%)
Age, years	15.6 (1.1)	16.0 (0.7)
BMI (kg/m^2^)	43.1 (5.2)	42.5 (5.6)
General cognitive functioning		
Estimated IQ	89.39 (14.31)	79.52 (16.73)
Vocabulary WISC-IV^b^ scaled score	6.85 (3.29; n = 13)	3.70 (3.50; n = 10)
Vocabulary WAIS-IV^b^ scaled score	8.50 (2.42; n = 10)	6.92 (1.66; n = 13)
Matrix reasoning WISC-IV^b^ scaled score	9.14 (2.51; n = 14)	7.46 (4.37; n = 13)
Matrix reasoning WAIS-IV^b^ scaled score	9.11 (3.06; n = 9)	8.60 (1.08; n = 10)
Digit span WISC-IV^b^ total scaled score	8.23 (2.28; n = 13)	6.30 (3.34; n = 10)
Digit span WAIS-IV^b^ total scaled score	9.40 (2.46; n = 10)	8.25 (2.49; n = 12)
Coding WISC-IV^b^ scaled score	8.08 (2.87; n = 13)	5.40 (3.44; n = 10)
Coding WAIS-IV^b^ scaled score	8.70 (2.41; n = 10)	8.46 (1.85; n = 13)
Verbal memory		
Immediate recall NEPSY-II raw score	55.52 (10.17)	52.70 (9.35)
Delayed recall NEPSY-II raw score	11.70 (3.02)	11.57 (2.21)
Executive functioning		
Verbal fluency		
Letter fluency D-KEFS scaled score	10.57 (4.21)	7.70 (3.24)
Category fluency D-KEFS scaled score	11.83 (4.52)	8.87 (3.05)
Category switching D-KEFS scaled score	10.39 (3.27)	9.87 (3.61)
Trail making test		
Visual scanning D-KEFS scaled score	8.61 (3.12)	8.91 (2.61)
Number sequencing D-KEFS scaled score	8.96 (3.28)	7.96 (3.70)
Letter sequencing D-KEFS scaled score	8.68 (3.58; n = 22)	5.52 (4.07)
Number-letter switching D-KEFS scaled score	8.05 (3.47; n = 22)	6.17 (2.93)
Motor speed D-KEFS scaled score	7.30 (3.71)	4.96 (4.10)
Colour-word interference test		
Colour naming D-KEFS scaled score	6.17 (2.23)	6.09 (3.13)
Word reading D-KEFS scaled score	7.30 (2.72)	6.91 (2.70)
Inhibition D-KEFS scaled score	7.78 (2.61)	6.86 (2.71; n = 22)
Inhibition/Switching D-KEFS scaled score	7.17 (2.86)	7.52 (3.64)
Mazes		
Elithorn mazes WISC-IV integrated raw score	34.60 (10.27; n = 20)	29.91 (11.61; n = 22)

SD, standard deviation; BMI, body mass index; IQ, intelligence quotient; WISC, Wechsler Intelligence Scale for Children; WAIS, Wechsler Adult Intelligence Scale; NEPSY-II, A Developmental NEuroPSYchological Assessment; D-KEFS, Delis-Kaplan Executive Function System.

a N = 23 except where noted.

b Participants were either assessed with WISC-IV (<16:0; n = 23), or WAIS-IV (≥16:0; n = 23). As the stimulus book for Matrix Reasoning from WAIS-IV was not available in the beginning at one centre, 4 participants completed 3 subtests with WAIS-IV and Matrix Reasoning with WISC-IV at baseline. Scaled scores have a mean of 10 and a standard deviation [SD] of ±3; range 1–19.

Of the 46 adolescents with a baseline cognitive assessment, 23 were assigned to bariatric surgery, and 23 to intensive non-surgical treatment. Three of the 46 adolescents (7%) had previously been diagnosed with a mild intellectual disability and were in special education. All three were randomised to intensive non-surgical treatment. Twenty-two of the adolescents allocated to bariatric surgery underwent surgery within two weeks after randomisation (21 RYGB and one sleeve gastrectomy). One adolescent initially hesitated and underwent surgery six months later (sleeve gastrectomy). One participant randomised to non-surgical treatment went abroad for surgery during the first year and was thereafter lost to follow-up. Two participants, one in each treatment group, participated partially in the one-year follow-up in AMOS2, but cognitive assessment was not carried out. During the second year of follow-up, five participants in the intensive non-surgical treatment, crossed over to undergo bariatric surgery (three RYGB and two sleeve gastrectomy). At the two-year follow-up, two participants randomised to non-surgical treatment were lost to follow-up. Three participants randomised to bariatric surgery and six participants randomised to intensive non-surgical treatment participated partially in the two-year follow-up in AMOS2 but did not carry out cognitive assessment at year two. For an overview of the number of patients in each group at each assessment point, please see Fig. 1.

### Baseline cognitive functioning

Baseline cognitive functioning for both groups is presented in Table 2. There was no significant correlation between BMI and any measure of cognitive functioning at baseline (all p > 0.05, data not shown).

### Qualitative interpretation of the estimated IQ-scores

**Fig. 2:**
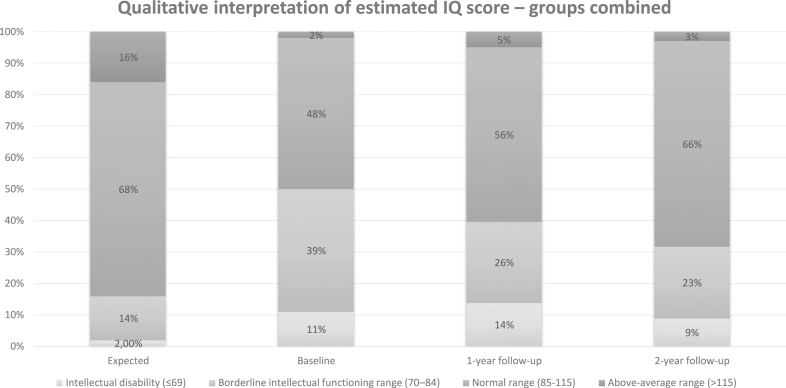
Qualitative interpretation of estimated IQ score at baseline and follow-ups. IQ, intelligence quotient. The proportion of participants in each category based on the estimated IQ score at baseline, one-year, and two-year follow-up presented for adolescents randomised to bariatric surgery and intensive non-surgical treatment together. The reference bar shows the expected proportion in the general population. Scores ≤69 are classified as intellectual disability, 70–84 as borderline intellectual functioning, 85–115 as normal range, and >115 above-average range. Baseline n = 46, 1-year follow-up n = 43, and 2-year follow-up n = 35.

The qualitative interpretation of the estimated IQ-scores at baseline and over follow-up is presented in Fig. 2. At baseline, 23/46 (50%) of the adolescents had an estimated IQ-score corresponding to a borderline intellectual functioning or lower. At the follow-ups at one and two years, the corresponding proportion was 17/43 (40%) and 11/35 (31%).

### Comparison between adolescents undergoing bariatric surgery and intensive lifestyle treatment over 2-years

Participants randomised to bariatric surgery lost significantly more weight than participants in the non-surgical treatment arm (Figure S1), with a mean BMI at two years 30.06 (±5.94) vs 41.99 (±8.14), p < 0.0001.

**Fig. 3:**
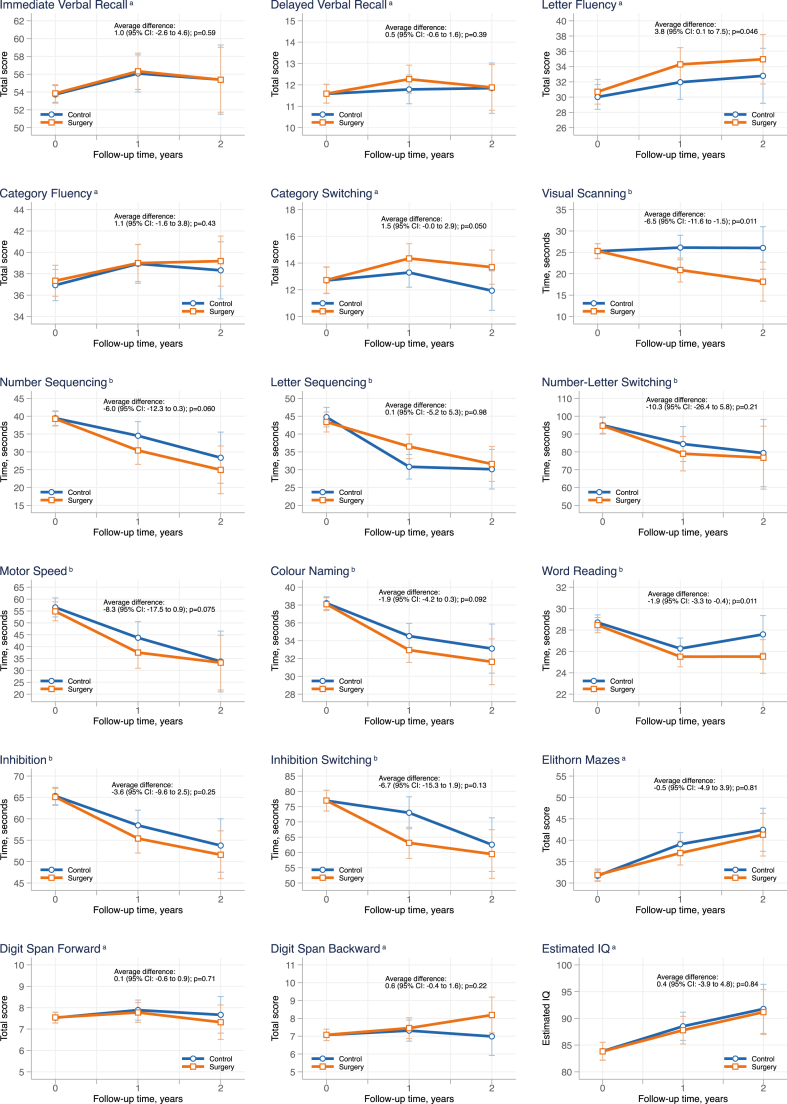
Cognitive functioning in adolescents with severe obesity over 2 years after random assignment to bariatric surgery or intensive non-surgical treatment. IQ, intelligence quotient. Estimated means with 95% CI: (confidence interval) bars from a mixed-effect regression model on tests assessing verbal memory, basic cognitive skills, executive functioning, and IQ in adolescents randomised to bariatric surgery or intensive non-surgical treatment over 2 years.^a^ A higher score indicates better performance.^b^ A shorter time indicates better performance. Data analysed as intention to treat with mixed-effects regression models, with adjustment for baseline value and stratification variables (sex and centre). The difference between groups is expressed as the average difference during the follow-up. Red lines represent adolescents randomised to bariatric surgery (participants with data at baseline [n = 23], one-year follow-up [n = 22], and two-year follow-up [n = 20]). Blue lines represent adolescents randomised to intensive non-surgical treatment (participants with data at baseline [n = 23], one-year follow-up [n = 21], and two-year follow-up [n = 15]).

Changes in cognitive functioning over two years adjusted for baseline values and stratification variables are shown in Fig. 3. In 15/18 (83%) of assessed aspects of cognitive functioning, no significant difference between the treatment groups was found. The average difference over two years was significantly different between the groups in three sub-tests from D-KEFS assessing fundamental cognitive skills; Letter Fluency, Visual Scanning, and Word Reading, with a greater improvement in the operated adolescents compared to the adolescents undergoing intensive non-surgical treatment (Fig. 3). One test assessing executive functioning, Category Switching, was borderline significant, p = 0.050.

Results in sensitivity analyses with multiple imputation of missing data remained mostly unchanged (Figure S2), but a statistically significant difference was evident in Category Switching between the groups, with higher performance among the operated adolescents over follow-up (average difference 1.6 [95% CI: 0.1–3.1] p = 0.038). The results from the per-protocol analysis, where the participants were analysed as treated, were similar to the results from the analysis with multiple imputations, but then also Motor Speed, a fundamental cognitive skill test from D-KEFS, was statistically different between the groups (average difference: −9.5 [95% CI: −18.8 to −0.2] p = 0.046; Figure S3) indicating greater improvement in the operated adolescents.

## Discussion

Impaired cognitive function has been suggested as a worrying potential consequence of the paediatric obesity epidemic.^4^ Preliminary findings in adults, showing substantial and sustained cognitive improvements after bariatric surgery,^7^^,^^16^ have sparked interest regarding the potential to attain such improvements in adolescents with severe obesity, who are at risk for poorer school performance and more school absenteeism compared to normal weight peers.^17^^,^^18^

In the present study, cognitive functioning was assessed in a sample of adolescents with severe obesity randomised to bariatric surgery or non-surgical treatment. Although bariatric surgery resulted in major weight loss and intensive non-surgical treatment resulted in almost no weight loss, we did not show an overall pattern of different outcomes for executive functioning and memory between the groups. However, the adolescents in the surgical group demonstrated a statistically significantly greater improvement in three tests assessing fundamental cognitive skills.

A systematic review and meta-analysis, published in 2022, including data predominantly from adult bariatric surgery patients, concluded that bariatric surgery significantly improves immediate verbal memory function and delayed memory function, but that these improvements might be limited to patients undergoing RYGB.^8^ In the present study the majority of the adolescents underwent RYGB (21/23; 91%), yet we found no differences in immediate or delayed verbal recall over two years between the groups.

Compared to obesity and weight loss in adulthood, it is unclear whether obesity and weight loss during adolescence affects cognitive functioning differently. A study from the USA in adults undergoing bariatric surgery showed less recovery of attention in participants who had had obesity during adolescence, and it was suggested that cognitive alterations associated with early onset obesity are less responsive to weight loss in comparison to those related to obesity with adulthood onset.^19^

Previous prospective studies reporting improvements in cognitive functioning after bariatric surgery usually follow patients before and after surgery without a relevant control group.^7^^,^^8^^,^^20^, ^21^, ^22^ However, studies that have included a non-operated comparator group appear to show no, or few, significant differences between the groups.^8^^,^^11^^,^^23^^,^^24^ The latter observation is in line with our findings in this study where, although we demonstrated improvements at follow-up in the surgical group in several cognitive tests, similar improvements were also observed in the non-surgical group, resulting in few significant differences between the groups.

The similar outcomes in cognitive functions between the treatment groups may have several explanations. Improvements in cognitive function over time may be explained by a positive effect of obesity treatment and care in general, not restricted to bariatric surgery.^25^^,^^26^ Another possible explanation for better results at follow-up might be a practice effect that is equal for both groups. A study in adults, however, showed that controls with obesity had a decline in memory at repeated testing.^9^ Animal and letter fluency have been recommended as appropriate tests for repeated testing in bariatric surgery samples due to low practice effect, and these tests were used in the present study (animal fluency is included in category fluency). However, we found no significant differences between the groups in any of these tests. There were several participants with weak performance at baseline, and improvements at follow-up can also be a result of regression to the mean.

Although we found few differences in cognitive function between groups, the adolescents undergoing surgery had significantly greater improvements in three subtests of D-KEFS compared to the control group. In contrast to previous studies in adults showing greater improvements in executive functioning or memory after bariatric surgery,^27^ the greater improvements in the surgical group were seen in three subtests assessing fundamental cognitive skills. Two of the tests, Letter Fluency and Word Reading, are related to verbal skills and one test, Visual Scanning, is related to visual attention. A systematic review highlighted that there are links between obesity and local lower gray matter volume or cortical thickness in several areas of the brain, as measured using MRI.^28^ Differences were mainly observed in frontal and temporal areas that overlap with language areas, such as those involved in verbal fluency and reading. Further studies in adolescents and adults are needed to confirm a relationship between bariatric surgery, the subsequent weight loss, specific improvements in fundamental cognitive skills, and whether they are clinically meaningful or not. Future studies could also look specifically at neural correlates between improved skills after bariatric surgery and local brain structure.

In line with an adolescent US study (n = 29), we did not find any significant association between baseline cognitive functioning and BMI among our participants.^29^ However, a larger (n = 141) US study found that a higher pre-surgical BMI was significantly associated with lower cognitive functioning.^6^

In this study, about half of adolescents with severe and treatment resistant obesity had a general intellectual functioning classified as borderline or below when assessed at baseline. These results should be interpreted cautiously, and not on an individual level, as an abbreviated scale was used. However, our findings corroborate previous data showing that children and adolescents with obesity more often have cognitive deficits compared to the general population.^3^^,^^4^ A reasonable interpretation is that a strong genetic and physiological propensity to develop obesity is more likely to penetrate in individuals with impaired cognitive abilities, thereby rendering them more likely to need obesity treatment.^5^^,^^30^

The substantial proportion of adolescents with a borderline general intellectual functioning or below, also indicates that interventions addressing adolescents with severe obesity must be adapted to be accessible to adolescents with learning difficulties. Of special relevance is the below average results on Vocabulary, indicating that interventions heavily dependent on verbal information should be supported with information in other modalities, such as visual information.

The proportion of adolescents with borderline or lower intellectual functioning was greater in the present study (50%) than in a previous US study of adolescents undergoing bariatric surgery (12.6%) where also an abbreviated version of a Wechler scale was used to estimate IQ.^6^ Yet another US study reported that 21% of adolescents presenting for bariatric surgery had an IQ <80.^29^ The differences in cognitive functioning might reflect differences in patient selection for adolescent bariatric surgery between Sweden and USA.

Thus, the adolescents included in AMOS2 might not be representative of all adolescents with severe obesity eligible for bariatric surgery. Three adolescents in the present study were in special education due to mild intellectual disability, and there was a high number of adolescents in AMOS2 screening positive for a neuropsychiatric disorder.^31^ Differences in cognitive functioning might affect generalisability between cohorts, since the effect of bariatric surgery and subsequent weight loss for improving cognitive functioning might be limited for adolescents with borderline or lower intellectual functioning compared to adolescents with intellectual functioning in the normal range or higher.

The detected significant differences between the surgical and non-surgical group should be interpreted cautiously as they could be caused by chance, which is a specific risk in an exploratory study with repeated significance testing. On the other hand, there could be important differences in cognitive outcomes that our sample was too small to detect. Cognitive functioning was not the primary end point of the AMOS2, and our study might have been underpowered to detect important differences. Fewer participants in the non-surgical group returned to the two-year follow-up, by which time one third had crossed over. Thus, the non-significant findings in this study should not be interpreted as a proof of non-existing differences.

Another limitation is that the adolescents in this study were informed that the data on cognitive functioning were collected to analyse outcomes and patterns for the full sample and not be used for planning education or interventions on an individual level. This may have resulted in some adolescents putting less effort in to their performance, than they would have if the assessment were performed under other, more personally relevant, circumstances.

General cognitive functioning had to be tested using two different Wechler scales (WISC-IV and WAIS-IV) based on the participants’ ages at assessments. Even though WISC-IV and WAIS-IV are developed by the same publisher, and we used the same subtests from respective test, findings from WISC-IV and WAIS-IV are not directly comparable with each other. The WISC-IV has more granulated norms (reported for 3 months at a time) compared to WAIS-IV (reported for 2 years at a time), which might generate higher resolution for participants tested with WISC-IV. This may explain the somewhat lower performances on WISC-IV compared to WAIS-IV on some subtests at baseline and some of the improvement in estimated IQ over time. However, such bias is supposed to affect both groups in a similar way.

Balancing the potential limitations described above, the study's main strength is its randomised design, particularly in comparison to previous studies on cognitive changes after bariatric surgery. Other strengths include the use of validated tests used in clinical practice, and test administration by clinical psychologists. The sample consisted of adolescents aged 13–16 years at baseline, a group for which it would be of particular relevance to offer a treatment to support cognitive function given the intense, and often highly consequential, phase of education and the generally pivotal time in these individuals' lives in determining potential future life trajectory.

In conclusion, this study contributes the first randomised data on cognitive functions and bariatric surgery. Bariatric surgery, which was associated with significant weight loss, was overall no more effective than intensive non-surgical treatment, without weight loss, in improving executive function or memory in adolescents over two years. However, the operated adolescents had significantly greater improvement in some fundamental cognitive skills, which needs to be confirmed in future studies with larger samples. Meanwhile, the substantial proportion of adolescents with borderline general intellectual functioning or below indicates that it is important to develop current treatment options and related information, so they are more easily accessible to adolescents with cognitive challenges.

## Contributors

KJ conceptualised and designed the study with input from JM and TO. KJ and EG planned and organised the data collection. KJ collected and prepared data. MP conducted the statistical analysis. KJ and MP had full access to and verify the underlying study data. KJ wrote the first draft of the manuscript. All authors undertook revisions and contributed intellectually to the development of this paper. All authors have approved the final draft of the manuscript.

## Data sharing statement

As the Adolescent Morbid Obesity Surgery 2 is an ongoing trial with long-term follow-up, data will not be shared before study completion. The protocol, analysis plan, and informed consent documents are included in the appendix.

## Declaration of interests

KJ and LS received speaker honoraria from Novo Nordisk unrelated to the submitted article. All reimbursements were directed to their clinical institutions (Skåne University Hospital [KJ] and Sahlgrenska University Hospital [LS]). AB participated in an advisory board for Ethicon unrelated to the submitted article (personal payment). TO participated in advisory boards and educational activities for Johnson & Johnson and Novo Nordisk and participate in a data safety monitoring board for the MAGNET study unrelated to the submitted article. All reimbursements were directed to his academic institution (Linköping University). All other authors declare no competing interests.
