# Supplementary material for: Cognitive functioning in adolescents with severe obesity undergoing bariatric surgery or intensive non-surgical treatment in Sweden (AMOS2): a multicentre, open-label, randomised controlled trial
Source: eClinicalMedicine. 2024 Feb 27;70:102505. doi: 10.1016/j.eclinm.2024.102505 (PMC11056597; doi:10.1016/j.eclinm.2024.102505)
Supplement: Supplementary Appendix [file mmc1.pdf]

Supplementary appendix

Supplement to: Järvholm K, Gronowitz E, Janson A, et al. Cognitive functioning in adolescents with severe obesity undergoing bariatric surgery or intensive non-surgical treatment: results from a 2-year multicentre, randomised, controlled trial in Sweden

Content

Inclusion and exclusion criteria 4

Inclusion criteria 4

Exclusion criteria 4

Supplementary figures 5

Figure S1. Change in bodyweight (kg) in adolescents with severe obesity over two years after random assignment to bariatric surgery or intensive non-surgical treatment 5

Figure S2. Cognitive functioning in adolescents with severe obesity over 2 years after random assignment to bariatric surgery or intensive non-surgical treatment with multiple imputation of missing data 6

Figure S3. Cognitive functioning in adolescents with severe obesity over 2 years after random assignment to bariatric surgery or intensive non-surgical treatment analysed according to the per protocol 7

Original protocol including a research plan 9

Translated original protocol 10

Original protocol (in Swedish) 20

Translated original research plan 40

Original research plan 46

Informed consent for participants aged ≥ 15 years. 51

Informed consent for caregivers 57

Assent for participants aged < 15 years 65

Amendments to the protocol 70

Translated amendment I to the ethical review board 70

Amendment I to the ethical review board 72

Translated specification to Amendment I to the ethical review board 73

Specification to Amendment I to the ethical review board 75

Translated amendment application II in AMOS2 77

Amendment application II in AMOS2 80

Summary of changes to the protocol 83

Statistical analysis plan 84

1. Study objectives 85

2. Study Design 85

2.1 Overview 85

2.2 Randomization 85

2.3 Eligibility criteria 85

2.4 Data collection and follow-up 85

3. Study outcomes 86

4. Statistical methods ... 86
4.1 Sample size and power ... 86
4.2 Principles of analysis ... 86
4.3 Statistical analyses ... 87
4.4 Missing data ... 87
5. References ... 87
6. Appendix ... 87

Inclusion and exclusion criteria

Inclusion criteria

- Age 13 –16 years
- BMI ≥ 35 kg/m^2
- Insufficient results from comprehensive treatment for obesity for at least one year
- Eligible according to the assessment of the clinical paediatric psychologist within the adolescent metabolic-bariatric multi-disciplinary team
- Pubertal stage Tanner 3 or higher

Exclusion criteria

- Monogenic or syndromic obesity (for example Prader Willi Syndrome, Laurence Moon – Bardet-Biedl)
- Obesity secondary to brain injury
- Severe intellectual disability or other severe, pervasive developmental disorder
- Unsuitable for general anaesthesia
- Psychosis or other major psychiatric illness (e.g. severe depression or suicide attempt during the last year)
- Self-induced vomiting to regulate weight
- Ongoing substance abuse
- Previous major gastrointestinal surgery

Supplementary figures

Figure S1. Change in bodyweight (kg) in adolescents with severe obesity over two years after random assignment to bariatric surgery or intensive non-surgical treatment

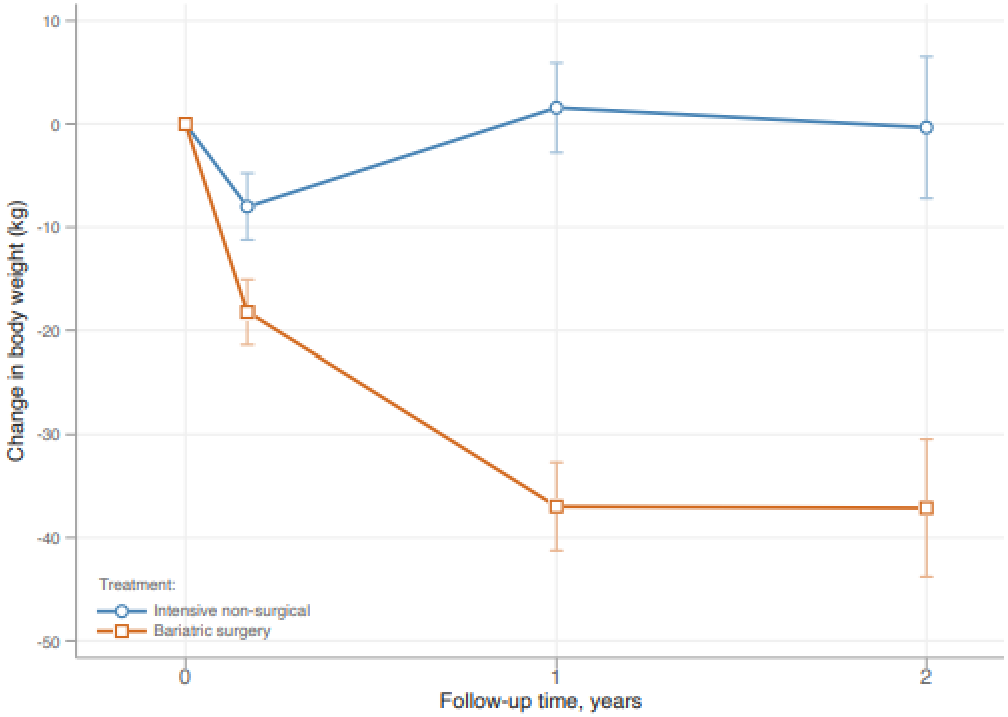

Estimated means with 95%-CI bars from a mixed-effect regression model. Data analysed as intention to treat with mixed-effects regression models, with adjustment for baseline value and stratification variables (sex and centre). Red lines represent adolescents randomised to bariatric surgery (participants with data at baseline [n=23], one-year follow-up [n=23], and two-year follow-up [n=23]). Blue lines represent adolescents randomised to intensive non-surgical treatment (participants with data at baseline [n=23], one-year follow-up [n=22], and two-year follow-up [n=21]).

Figure S2. Cognitive functioning in adolescents with severe obesity over 2 years after random assignment to bariatric surgery or intensive non-surgical treatment with multiple imputation of missing data

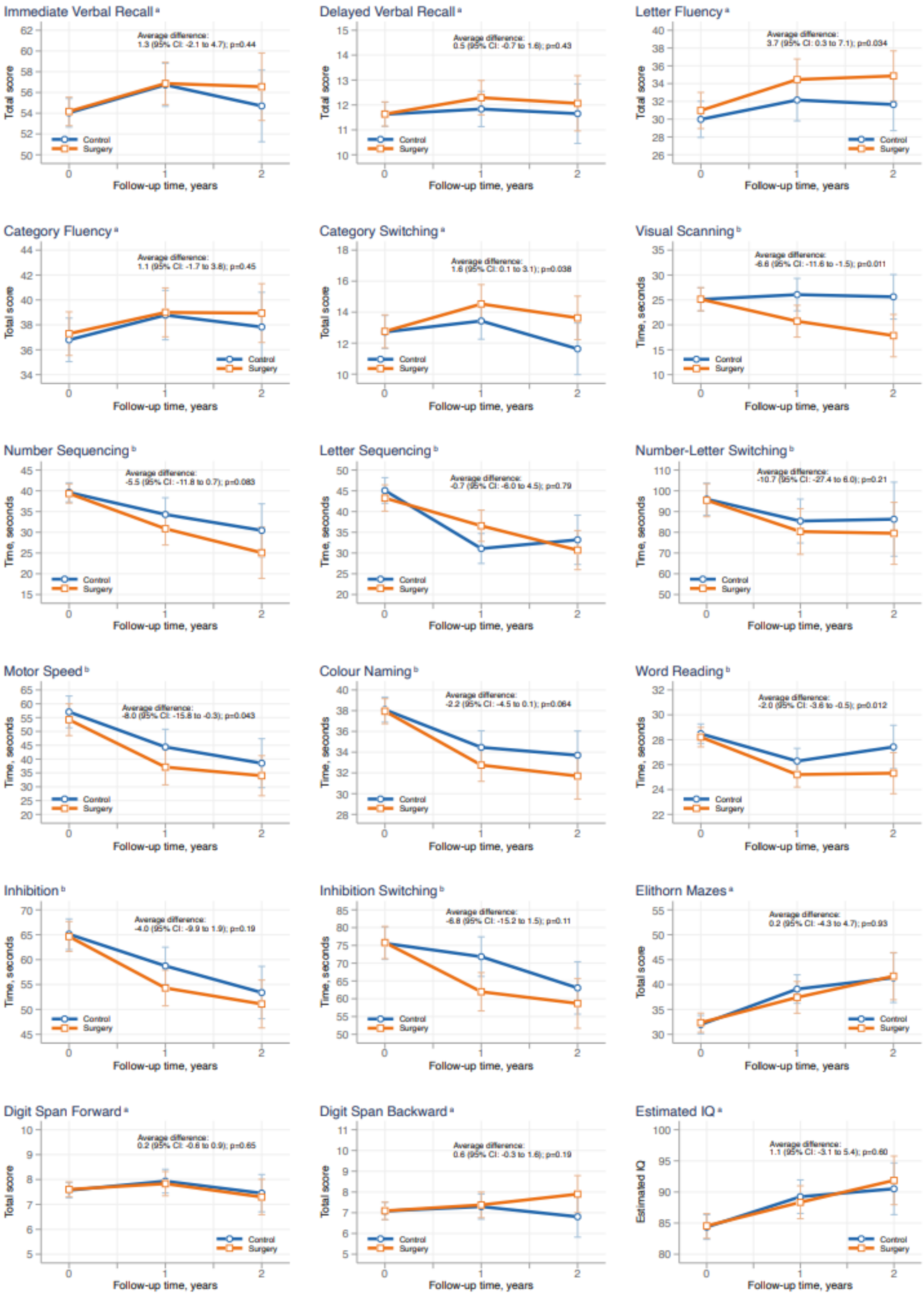

IQ = intelligence quotient. [a]A higher score indicates better performance. [b]A shorter time indicates better performance. Estimated means with 95%-CI bars from a mixed-effect regression model. Data analysed as intention to treat, with multiple imputation of missing data, with mixed-effects regression models, with adjustment for baseline value and stratification variables (sex and centre). The difference between groups is expressed as the average difference during the follow-up.

Red lines represent adolescents randomised to bariatric surgery (participants with data at baseline [n=23], one-year follow-up [n=23], and two-year follow-up [n=23]). Blue lines represent adolescents randomised to intensive non-surgical treatment (participants with data at baseline [n=23], one-year follow-up [n=23], and two-year follow-up [n=23]).

Figure S3. Cognitive functioning in adolescents with severe obesity over 2 years after random assignment to bariatric surgery or intensive non-surgical treatment analysed according to the per protocol

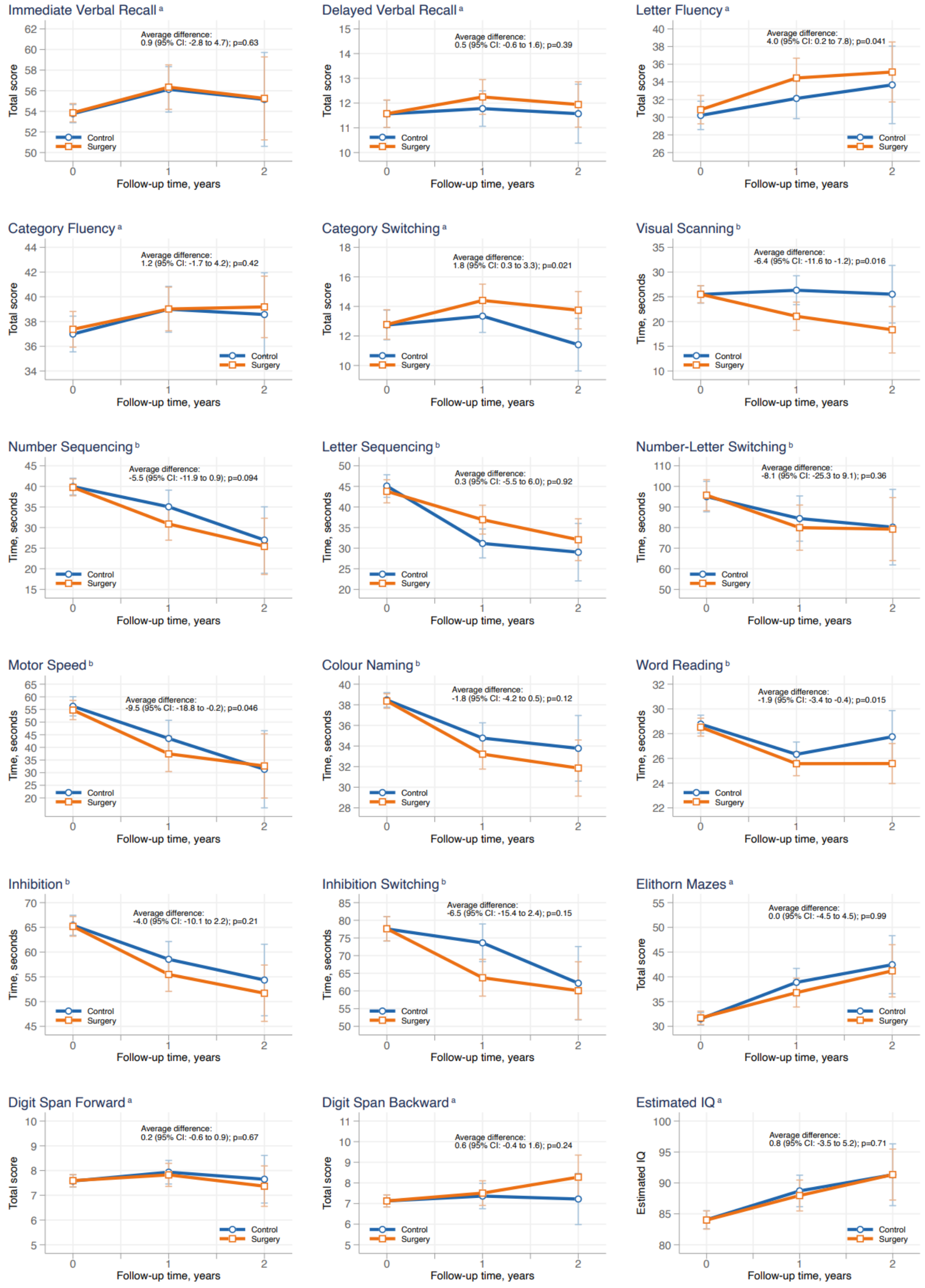

IQ = intelligence quotient. [a]A higher score indicates better performance. [b]A shorter time indicates better performance. Estimated means with 95%-CI bars from a mixed-effect regression model. Data analysed as per protocol (i.e. persons who crossed-over to the other treatment arm were censored at the corresponding time-point, and their data after conversion was not used in the estimation of the treatment effect) with mixed-effects regression models, with adjustment for baseline value and stratification variables (sex and centre). The difference between groups is expressed as the average difference during the follow-up.

Red lines represent adolescents randomised to bariatric surgery (participants with data at baseline [n=23], one-year follow-up [n=22], and two-year follow-up [n=20]). Blue lines represent adolescents randomised to intensive non-surgical treatment (participants with data at baseline [n=23], one-year follow-up [n=21], and two-year follow-up [n=10]).

All documents are from the AMOS2 study

Original protocol including a research plan

(written in Swedish from December 2013 and approved by the ethical review board in January 2014)

An overview of the protocol has been published in Contemporary Clinical Trials Communications in 2020.
https://doi.org/10.1016/j.conctc.2020.100592

This document contains the following items:

1. Original protocol with a research plan (translated and in Swedish) approved by the ethical review board.
2. The amendments approved by the ethical review board (translated and in Swedish).
3. A summary of the changes from the original protocol.

Translated original protocol

Randomized trial between surgical and medical treatment for adolescents between 13 and 15 years of age with severe obesity

1. Responsible parties for the research

Responsible organization:

Sahlgrenska Universitetssjukhuset, Drottning Silvias Barn o Ungdomssjukhus, 41345 Göteborg

Representative for responsible organization:

Ralph Bågenholm, Chief of Staff, VO1, Drottning Silvias Barn och Ungdomssjukhus, 416 85 Göteborg

Principal investigator:

Torsten Olbers, Senior Consultant Surgeon, Dept of Surgery, Kirurgiska kliniken, Sahlgrenska sjukhuset 41345 GÖTEBORG

Additional collaborators:

Jovanna Dahlgren, Drottning Silvias Barn och Ungdomssjukhus, 41685 Göteborg

Annika Jansson Rikscentrum Barnobesitas, Huddinge sjukhus 141 52 Huddinge

Eva Gronowitz, Drottning Silvias Barn och Ungdomssjukhus, 41685 Göteborg

Kerstin Ekbom, Rikscentrum, Karolinska Universitetessjukhuset Huddinge 141 52 Stockholm

Gunnar Göthberg, Drottning Silvias Barn och Ungdomssjukhus, 41685 Göteborg

Peter Friberg, Avd för klinisk fysiologi Sahlgrenska Universitetssjukhuset 413 45 Göteborg

Sven Klaesson, VO Kvinnor och Barn, Södertälje sjukhus AB 152 86 Södertälje

2. Information about the project

Summary

We are applying to conduct a randomized trial for adolescents between the ages of 13 and 15 with severe obesity. The young people are randomized to optimized medical care or to comprehensive care that also includes gastric bypass surgery. A prerequisite for participation in the study is that both the young person and guardian can imagine accepting surgical treatment.

The purpose of the study is to evaluate whether there are advantages to carrying out bariatric surgery early in adolescence compared to waiting until late adolescence or adulthood.

Background:

Obesity is one of the biggest challenges for future healthcare as it can lead to an increased risk of a number of diseases, such as type 2 diabetes, cardiovascular disease, cancer and premature death. Of particular concern is the high prevalence of obesity among children and adolescents.

The likelihood that a teenager suffering from severe obesity will continue to have a severe weight-problems in adulthood is very high. There are currently few or no conventional treatments that have shown good results for severe obesity in adolescence.

For adults and adolescents from the age of 18, bariatric surgery is the only treatment that has demonstrated long-term good results regarding weight loss (>20 years). The permanent weight loss leads to a lower incidence of cardiovascular risk factors, reduced morbidity (such as type 2 diabetes, heart attack, stroke, cancer), increased quality of life and longer life expectancy.

In 2006, a national Swedish study - AMOS - was started, in which a total of 81 young people between the ages of 13 and 18 underwent surgery after conservative weight treatment had failed. They were operated on with laparoscopic gastric bypass and a control group of 81 teenagers matched for sex, age and BMI were identified from the childhood obesity register in Sweden (BORIS) and followed in parallel. The two-year results show that the weight loss and metabolic improvements after gastric bypass are completely comparable to what was previously shown in adults. The surgery was generally well tolerated by the teenagers.

We are now carrying out five-year follow-ups of the 81 young people who underwent gastric bypass as well as the matched control group who received traditional obesity treatment and in an adult group who underwent gastric bypass surgery during the same time-period. The results so far are positive.

Expected benefit:

The expected benefit of surgery is to provide the youth with a normalized psychosocial development in the sensitive period of late adolescence and young adult life. At the same time, there is a possible profit in not living with risk factors for metabolic disease during adolescence when the "disease map" during life is imprinted.

The primary outcome variable is weight loss and weight control. Secondary outcome variables include cardiovascular risk factors, development of vascular changes, quality of life, eating patterns, social, economic, and psychological development. We also want to evaluate the effects of the surgery regarding physical development, vitamin levels, surgical side effects and other potential negative aspects.

The young people in the group who are offered conservative therapy will be offered surgery, if requested, when they have reached the age of 18, and in exceptional cases between the ages of 16 and 18 if they develop a serious obesity-related disease or on other clinical grounds are judged to have an indication for operative treatment.

Which scientific questions underpin the design of the project?

Are there advantages to offering surgery in the early adolescence in comparison to receiving conservative care and waiting until after age 18?

Is the metabolic control (blood sugar, blood fat, blood pressure, inflammation, etc.) better in the group that undergoes surgical treatment than those receiving non-surgical treatment?

Is surgery well tolerated by those who have surgery early in their teens?

Can the operation restore the quality of life to the corresponding norm for the age group?

Is the social, economic, and educational development improving?

Is the physical/skeletal development normal after gastric bypass surgery in the lower teens?

Are there differences regarding appearance of excess skin after weight loss and the quality of life in adolescents undergoing bariatric surgery, differences between early and late adolescence?

Will those randomized to conventional therapy want to undergo surgery at a later stage?

Can healthcare and drug consumption be reduced through surgical treatment?

Previous experiences from animal studies?

Not relevant. The operations are already performed on humans.

Overviewing description of the methods, data collection, and data characteristic

In this study, we plan to recruit 13–15-year-olds with severe obesity (iso-BMI >35) who can consider undergoing bariatric surgery with gastric bypass. They will be informed of our so far cautiously positive results from the previous studies, but that we do not know if it is preferable to operate on them during early adolescence or to carry out continued intensive medical treatment and possibly operate later.

The adolescents are randomized to either:

1. Undergo gastric bypass surgery in the context of a multi-disciplinary care.

2. Intensive non-surgical program for the treatment of obesity. This means an initial Low Calorie Diet period of 8 weeks followed by continued lifestyle treatment with diet and exercise advice and possibly drug treatment.

Because the Swedish current practice does not include operative treatment of obesity under the age of 18, the controls will remain unoperated until they turn 18. For individuals who progress in their obesity in a serious way or develop serious morbidity, surgery may also be considered for those in the noon-surgical arm between the ages of 16 and 18.

In the surgical arm, a laparoscopic gastric bypass is performed using the technique described by Olbers et al and used in AMOS. The perioperative risks in gastric bypass have been low in our preliminary study of 81 patients (two blood transfusions and one antibiotic treatment due to deep infection). In the long term, there is a risk of

developing iron deficiency anaemia and certain other deficiencies due to reduced mineral/vitamin absorption. An observation in our preliminary study that the young people's compliance with vitamin supplementation was poor. We will close the mesenteric defects to minimize the risk of so-called internal herniations causing intestinal obstruction and provide preventive treatment against gallstone formation.

The operations in the randomized study will be performed in collaboration between the Carlanderska Foundation and the Departments of Surgery and Paediatrics at Sahlgrenska University Hospital in Gothenburg. Outpatient visits (inclusion visits and postoperative follow-ups) will take place both at Queen Silvia's children's and youth hospital and at the children's clinic at Astrid Lindgren's children's hospital.

In the medical arm, the study participants will be followed during the study period with intensive multidisciplinary care with regular visits to the obesity clinics at Drottning Silvia's Children and Youth Hospital and at the National Centre for Childhood Obesity, Karolinska Hospital, where the best available treatment for obesity will be given (including medication).

A comprehensive baseline survey will be conducted. The first return visit takes place 8 weeks after initiation of therapy. Further study visits will take place at 1, 2, 5 and 10 years and when adverse events are identified. Study examinations will also be carried out when the operated persons turn 20, 25 and 30 respectively. In addition to these study visits, the young people will also be on additional clinical visits where time coordination is sought to the greatest extent possible.

Inclusion criteria:

• Age 13-15 years

• Iso-BMI ≥ 35 kg/m2

• Completed at least one year of adequate conventional weight-reducing treatment.

• Puberty maturity Tanner >3

• Passed assessment by a psychologist

• Positive attitude to long-term follow-up.

Exclusion criteria:

• Severe lack of compliance

• Previous major abdominal surgery

• Specific obesity syndrome such as Prader Willis, Laurence Moon-Bardet-Biedl

• Obesity secondary to damage to the CNS

• Puberty stage Tanner ≤ 3 or lack of puberty

• Psychotic illness

• Mentally disabled

• Severe general illness and therefore unsuitable for general anaesthesia

• Leptin deficiency or homozygous MC4R defect

Examinations at the study points will be carried out as below:

The visit to the reception will take about 4-5 hours depending on the special needs of different individuals

Questionnaire

For quality of life: RAND-36, OP scale (obesity-specific QoL)

Mental Health: Beck Youth Inventory, 5-15, ASSQ, ARSR, Rosenberg Self Esteem, Mood Adjective Check List

Physical activity/ Eating patterns: Diet history, TFEQ-R21, QEWP-R, IPAQ

Gastrointestinal function: Gastro-Intestinal symptom rating scale (GSRS), Dumping Symptom Rating Scale (DSRS)

Abuse: AUDIT o DUDIT

The questionnaires are validated, and most were used in the previous ethics-reviewed study AMOS Dnr 523-04

Blood sample analysis

Total cholesterol, HDL-cholesterol, LDL-cholesterol and triglycerides and apo-lipoproteins as well as plasma insulin o Glucose and HbA1c will be analysed In addition, we will analyse a range of inflammatory markers (supersensitive CRP, IL-6, TNF-alpha and Adiponectin) as well as analysis of steroid hormones by mass spectrometry.

Genetic samples for screening known obesity genes will be investigated with snip analyses and methylation studies. The purpose of these investigations is partly to get an idea of whether certain genetic profiles respond less well to surgical therapy and partly to be able to give the young people and their guardians information if there was a genetic underlying background to the development of severe obesity, which in that case could be helpful

Blood sampling will be collected with a total of 50 ml of extra blood at the same time as the routine blood sampling at the start of the study, 2, 6, 12 and 24 and 60 months after the start of the study. Accumulated amount extra over 5 years = 250 ml

Endothelial function measurements

Endothelial function will be assessed using reactive hyperaemia peripheral and arterial tonometry (pulse wave velocity)

Ultrasound measurements with high-resolution ultrasound at 55 MHz (Visualsonics)

24-hour blood pressure measurement. Sleep examinations in the home environment.

Development of excess skin and physical activity level and performance are examined.

Health economic analysis

We will prospectively collect accumulated healthcare costs in both groups.

We also plan to follow up on operated young people and controls regarding potential side effects, need for medication, and health economic outcomes by linking clinical data to national registers such as the Patient Register, the Medicines Register, the Cancer Register and the Cause of Death Register at the National Board of Health and Welfare, as well as the Social Insurance Agency's register of sick leave and sickness/activity compensation. This has previously been done within the controlled clinical trial Swedish Obese Subjects, in which representatives from the research group have been involved. Migration data and socio-economic variables (education, marital status, income) are planned to be obtained from Statistics Norway.

Body composition

For body composition, DEXA will be used (minimum radiation dose approx. 0.002mSv) The examination takes approx. 15 min.

We will develop a digital treatment support (website, app) that is adapted for the youth group. The digital treatment support aims to function partly to increase adherence to the changed lifestyle (mainly regarding diet and exercise) and to ensure the group's information needs. The digital tool will be adapted to the youth group's special needs regarding language use, user-friendliness, and interactivity.

We will also try a dietary supplement, which is specially designed to meet the specific need for daily supply of extra vitamins and minerals after gastric bypass in young people. The tablet replaces other supplements (which have been shown to have poor adherence in teenagers) in one and the same tablet taken 1-3 times per day. Supplementation of vitamins and minerals is important in gastric bypass patients to avoid deficiency conditions.

Description of biobank

Whole blood, serum and EDTA plasma are stored in a minus 80-degree freezer at the Growth Laboratory, Centre for Paediatric Growth Research. Queen Silvia's Children and Youth Hospital. The samples are stored in a biobank in accordance with the National Board of Health and Welfare's regulations (in accordance with the 2002 Act; 297).

Written consent is obtained from the research subjects before sampling. The blood samples are coded in the same way as other data within the study and only the person in charge of the study has access to the code key. Conditions for the release of samples involve an ethical review and consent from the relevant research persons.

Documentation, data protection, and archive

Results and data are registered in an e-CRF which is in a secure database with a login that has been previously used in AMOS. Data will also be entered into the national quality registers BORIS- (Child Obesity Register in Sweden) and SOReg- (Scandinavian Obesity Surgery Registry) and, according to current rules, a note in the patient's hospital record about study participation. In the study's compilation for statistical analysis, the data is available in de-identified coded form. The code key is kept under lock and key and only the coordinator and supervisor have access to it.

Describe previous experiences (own and others) of the planned treatment

In 2009, JAMA published a randomized study from Melbourne, Australia, where surgery with so-called laparoscopic gastric banding led to better two-year outcomes than conservative treatment. Several other smaller series of operations on adolescents using different surgical techniques have shown that operating on teenagers appears to be safe and generally associated with positive outcomes.

In 2006, we started AMOS (Adolescent Morbid Obesity Surgery study) in Sweden, where we chose to start a non-randomized comparative study as sufficient safety and long-term data were lacking. A total of 81

adolescents between 13 and 18 years of age, who failed conservative weight management, were operated on with laparoscopic gastric bypass at the same time as a control group of 81 teenagers who were matched for sex, age and BMI were identified in the childhood obesity register in Sweden (BORIS).

Several articles have been published from AMOS with 100% follow up. We describe that the group generally tolerated the operation well and experienced improved in self-image and quality of life, while a sub-group needed more psychological support. We also show that the operated group reduces the body weight as much as a comparative adult control group and that significant and broad improvements in cardiovascular risk factors, and large improvements regarding both psychological and physical quality of life.

3. Information regarding research participants

Selection of sample

Young people with will be recruited by the fact that the highly specialized centres that are part of the study and treat children with severe obesity (University Hospital in Malmö, Queen Silvia's Children's and Youth Hospital in Gothenburg and Rikscentrum barnobesitas, Astrid Lindgren's Children's Hospital in Stockholm) in their regular activities identify and ask young people with iso-BMI >35 to participate in the study. The paediatric obesity doctor assesses whether the young people have received standard available treatment for obesity and whether the young people have made serious attempts at weight reduction. The paediatric obesity doctor also rules out contraindications and gives the young people information about the possibility of joining the planned study and what it entails.

The 13-to-15-year old's who meet the criteria to participate in the randomized study will be asked to participate.

What is the relation between researcher and research participants?

Doctor and patient

Describe the statistical assessment for power calculation

Weight development (calculated as permanent reduction in BMI) is the primary outcome variable. However, we value that it is comparably important to, in addition to weight, study the outcome for secondary outcomes such as quality of life, psychosocial development, cardiovascular risk factors, structural cardiovascular changes and stress problems.

We have sufficient power regarding the primary outcome variable (BMI development) to reach relevant statistical significance (>95% at the 0.01 level) already with 10 youth in each group. To assess secondary outcome variables, such as metabolic and cardiovascular data, we estimate that 25 patients in each group are sufficient to achieve 80% power at alpha of 0.05 based on our preliminary study of 81 patients. Regarding the young people's own perceived health, our study shows that a large majority of our patients get an increased quality of life and that 25 individuals in each arm provide good power for differences at the 0.05 level. By aiming to include 60 young people (30+30), we get a guarantee that even with a 10-20% dropout, we have sufficient statistical power

Could the research participants be asked to participate in additional projects?

Nothing planned.

What type of insurance is covering the participants?

Patient´s insurance applies.

Any economic compensation to participants?

Under certain circumstances reimbursement for travel expenses.

4. Information and informed consent

The procedure for delivering information to the participants

Paediatric obesity doctors will ensure that the young people have received or will receive commonly available counselling for obesity and that they have made serious attempts at weight reduction, as well as provide them with information both verbally and in writing about the possibility of joining the planned study. If there is interest in participating in the study, specific information and evaluation is given by the obesity surgeon and other paediatric medical staff in the form of paediatricians, paediatric nurses, psychologists and dieticians.

After thinking through the information for a good period of time, the young person and their guardian are given the opportunity to once again ask questions to both paediatric and surgical experienced people.

The written information is provided in two variants; one adapted for young people and one for their guardians.

From whom informed consent is obtained

Written informed consent is obtained from both adolescents and their guardians.

5. Research ethics consideration

Describe risks involved for the research participants

Although we have good safety data from a study of operations on young people under the age of five (the AMOS study), it may still be the case that it is not beneficial for everyone to have surgery under the age of 18. Above all, we do not know whether it is beneficial to operate in the early adolescence or wait until after age 18. Complications and side effects of surgery can lead to discomfort.

In the randomized study, there is no option to choose surgical treatment and half of those included in the study will continue to undergo conservative treatment, which can be perceived as a disappointment after they were able to consider surgery. By offering an intensified treatment in the conventional arm, we want to minimize the feeling of perceiving it as the "worse" alternative.

An extension of regular outpatient visits in connection with the treatments will be used for filling in questionnaires and extra examinations. A limited amount of blood (50 ml) extra will be collected at each regular blood sampling occasion.

Some of the intended questionnaires can be perceived as intrusive and there is a risk that the young people find it difficult to describe their situation. All questionnaires are filled in together with trained staff who also have the

experience to take on continued responsibility for further care if the need arises. Some of the questionnaires are used within conventional treatment of obese young people today, and the staff thus have a wide experience of the questionnaires and what their outcome can mean.

All quality registry data is collected routinely, so subjects are not exposed to any additional risks or time-consuming investigations. At the same time, important data on, for example, side effects and health economic variables can be collected, which is difficult to do with full coverage in other ways. Thus, the risks are largely non-existent while there is great benefit in combining clinical data with registry data. However, the privacy aspect is brought up to date, as some study participants could experience this as a breach of privacy. To eliminate this risk of perceived privacy invasion, informed consent is required. Furthermore, results will only be presented at group level so that individual individuals cannot be tracked.

Describe possible benefits by participating in the research

Most of the evidence suggests that the positive effects that can be achieved in adults undergoing surgery can also be achieved in the operation of young people under the age of 18. We therefore want to give the young people between 13 and 15 years of age with particularly severe obesity and medical complications an opportunity to undergo surgery, however in the form of a continuing study. We consider ourselves to have a responsibility to carry out the randomized study which, in that case, can show that surgical treatment is also recommended for teenagers.

Half of those included in the randomized study get the opportunity to undergo surgery and its possible positive consequences, while the conservatively treated group is offered a treatment that is usually more intensive than usual practice for young people.

The goal is to close the knowledge gap that currently exists within healthcare concerning if individuals under the age of 18 with severe obesity can be returned to a normalized life course by carrying out an admittedly radical but life-changing operation. If this is the case, a previously very vulnerable group in society with very high expected morbidity, low quality of life and greatly reduced life expectancy will be able to be offered effective and life-enhancing treatment. Even from a societal perspective, costs will be able to be reduced through reduced morbidity, sick leave etc. that arise because of obesity.

Identify and define possible ethical dilemmas with the research

The ethical dilemma includes that young people with limited autonomy and difficulty in seeing lifelong consequences of their choice to undergo surgical treatment. On the other hand, it is ethically questionable not to be able to offer this potentially life-enhancing treatment to young people in the current lack of alternative effective therapies.

The National Board of Health and Welfare's register routinely collects important data on, for example, side effects and health economic variables, which is difficult to do with full coverage in other ways. The risks are largely non-existent while there is great benefit in combining clinical data with registry data. To eliminate the risk of a perceived breach of privacy, informed consent is required. Furthermore, results will only be presented at group level so that individual individuals cannot be tracked.

Our overall assessment is that it would be unethical not to continue to systematically study the value of surgical treatment for young people with severe obesity in this way.

6. Reporting the results

Who is responsible for data analysis and reporting?

The data are handled by the responsible party for the research (Sahlgrenska University Hospital). The principal investigator, Dr Torsten Olbers, is responsible for data analysis and report writing.

How will data be made publicly available?

Data will be published as articles in international journals and presented at international conferences.

How will you secure the integrity of the research participants at publication?

All data from the study are presented in de-identified form. No data that can be traced back to a single person may be presented without express permission being obtained from the person concerned and their guardian.

7. Economic relationship and dependency relationship

Economic relationship and dependency relationship

Overall promise of co-financing over three years:

Brainbridge SEK 97,000

Johnson&Johnson SEK 80,000

Training of personnel in obesity surgical care.

Account for conflicts of interests of the responsible party for the research, principal researcher, and other participating researchers

Nothing relevant

8. Signatures

Ralph Bågenholm

Torsten Olbers

Original protocol (in Swedish)

ANSÖKAN OM ETIKPRÖVNING 1

ANSÖKAN OM ETIKPRÖVNING AV FORSKNING SOM AVSER MÄNNISKOR

Information till ansökan, ***se Vägledning till ansökan (www.epn.se)***

Beroende på vilken forskning som ansökan gäller kommer de uppgifter som efterfrågas att ha olika relevans. Vid ändring av tidigare godkänd ansökan, se ***Vägledning till ansökan*****.**

Till Regionala etikprövningsnämnden i: Göteborg

Den regionala etikprövningsnämnd till vars upptagningsområde forskningshuvudmannen hör, se respektive nämnd *(www.epn.se)*.

Avgift inbetald datum: 130620

Observera att en ansökan aldrig är komplett och därmed kan behandlas förrän blanketten är korrekt ifylld och avgiften är betald.

Projekttitel: Randomiserad studie mellan kirurgisk och medicinsk behandling för ungdomar mellan 13 och 15 år med svår fetma

Ange en beskrivande titel på svenska för lekmän. Titeln ska ej innehålla sekretesskyddad information. Ange också i förekommande fall, t.ex. vid klinisk läkemedelsprövning, projektets identitet, forskningsplanens/protokollets nummer, version, datum. Vid ändring av tidigare godkänd ansökan, se Vägledning till ansökan.

Projektnummer/identitet: Version nummer: EudraCT nr (vid läkemedelsprövning):

Uppgifter som fylls i av den regionala etikprövningsnämnden

Ansökan komplett: Dnr:

Begäran om ytterligare information (i sak): Begärd information inkommen:

Beslutsdatum: Expeditionsdatum:

Ansökan avser (gäller även vid begäran om rådgivande yttrande):

Forskning där endast en forskningshuvudman deltar (5 000 kr)

Forskning där mer än en huvudman deltar (16 000 kr) ANSÖKAN OM ETIKPRÖVNING 2

Forskning där mer än en forskningshuvudman deltar, men där samtliga

forskningspersoner eller forskningsobjekt har ett omedelbart

samband med endast en av forskningshuvudmännen (5 000 kr)

Endast behandling av personuppgifter (5 000 kr)

(När enbart redan befintliga personregister ska användas, t. ex. nationella databaser)

Forskning som gäller klinisk läkemedelsprövning (16 000 kr)

Ändring av tidigare godkänd ansökan enligt 4 § förordning (2003:615) om

etikprövning av forskning som avser människor (2 000 kr)

Om nämnden finner att forskningsprojektet inte faller inom etikprövninglagens tillämpningsområde

önskas ett rådgivande yttrande. (Se 4a och 4b §§ i förordning 2003:615 och Vägledning till ansökan)

Ja: Nej:

1. Information om forskningshuvudman m.m.

1:1 Forskningshuvudman (Se p. 1:1 i Vägledning till ansökan)

Ansökan om etikprövning av forskning ska göras av forskningshuvudmannen. *Med forskningshuvudman avses en statlig myndighet eller en fysisk eller juridisk person i vars verksamhet forskningen utförs.*

Namn: Sahlgrenska Universitetssjukhuset, Drottning Silvias Barn o Ungdomssjukhus

Adress: 41345 Göteborg

1:2 Behörig företrädare för forskningshuvudmannen

Behörig företrädare är t.ex. prefekt, enhetschef, verksamhetschef. Forskningshuvudmännen bestämmer själva, genom interna arbets- och delegationsordningar eller genom fullmakt, vem som är behörig att företräda forskningshuvudmannen.

Namn: Ralph Bågenholm Tjänstetitel: Verksamhteschef

Adress: VO1, Drottning Silvias Barn och Ungdomssjukhus, 416 85 Göteborg

1:3 Forskare som är huvudansvarig för genomförandet av projektet (kontaktperson) (Se p. 9 bil. nr 10 och p. 1:3 i Vägledning till ansökan)

Observera! Den som är huvudansvarig forskare ansvarar för att andra medverkande som ska genomföra projektet har tillräcklig kompetens (vetenskaplig och klinisk) och vid läkemedelsprövning har tillräcklig kunskap om ”Good Clinical Practice” (GCP). Vid doktorandstudier är som regel handledaren huvudansvarig forskare.

Namn: Torsten Olbers Tjänstetitel: Överläkare

Postadress: Kirurgiska kliniken, Sahlgrenska sjukhuset 41345 GÖTEBORG

E-postadress: torsten.olbers@gu.se

Telefon: 031-3428347

Mobiltelefon: 0736-601729 ANSÖKAN OM ETIKPRÖVNING 3

1:4 Andra medverkande (Se p. 9 bil. nr 1 och p. 1:4 i Vägledning till ansökan)

Övriga deltagande forskningshuvudmän samt forskare ansvariga för att lokalt genomföra projektet (kontaktpersoner) anges här eller i bilaga med namn och adresser (se p. 9 bil. nr 1).

Jovanna Dahlgren, Drottning Silvias Barn och Ungdomssjukhus, 41685 Göteborg

Annika Jansson Rikscentrum Barnobesitas, Huddinge sjukhus 141 52 Huddinge

Eva Gronowitz, Drottning Silvias Barn och Ungdomssjukhus, 41685 Göteborg

Kerstin Ekbom, Rikscentrum, Karolinska Universitetessjukhuset Huddinge 141 52 Stockholm

Gunnar Göthberg, Drottning Silvias Barn och Ungdomssjukhus, 41685 Göteborg

Peter Friberg, Avd för klinisk fysiologi Sahlgrenska Universitetssjukhuset 413 45 Göteborg

Sven Klaesson, VO Kvinnor och Barn, Södertälje sjukhus AB 152 86 Södertälje

1:5 Redovisa tillgång till nödvändiga resurser under projektets genomförande

(Se p. 9 bil. nr 9 och p. 1:5 i Vägledning till ansökan)

Ange vem/vilka som har ansvaret (prefekt, verksamhetschef eller motsvarande) för forskningspersonernas säkerhet vid alla enheter/kliniker där forskningspersoner ska delta. Intyg från dessa ansvariga *ska* bifogas (se p. 9 bil. nr 9). Av intyget ska framgå att erforderliga ekonomiska, strukturella och personella resurser finns tillgängliga för att garantera forskningspersonernas säkerhet.

§

1:6 Ansökan/anmälan till andra myndigheter i vissa fall

(se p. 1:6 i Vägledning till ansökan) **Insänd Datum**

a)Vid klinisk läkemedelsprövning: Läkemedelsverket

b)Vid hantering av personuppgifter om genetiska anlag: Datainspektionen

c)Vid inrättande av biobank: Socialstyrelsen

d)Vid undersökning omfattande joniserande strålning: Strålskyddskommitté ANSÖKAN OM ETIKPRÖVNING 4

2. Uppgifter om projektet

2:1 Sammanfattande beskrivning av forskningsprojektet (Se p. 9 bil. nr 2 och p. 2:1 i Vägledning till ansökan)

Beskrivningen ska kunna förstås av nämndens samtliga ledamöter. Undvik därför terminologi som kräver specialkunskaper. Ange bakgrund och syfte för studien samt den/de vetenskapliga frågeställning (ar) som man

söker svar på. Ange de viktigaste undersökningsvariablerna. Beskriv vilka kunskapsvinster projektet kan förväntas ge och betydelsen av dessa. Ange om det är en registerstudie, uppdragsforskning etc. För fackmän avsedd detaljerad information om forskningsplan/protokoll (program) *ska* bifogas som bilaga (se p. 9 bil. nr 2). För utformning av forskningsplan/protokoll se p. 2:1 i Vägledning till ansökan. Ange när datainsamlingen beräknas vara avslutad. En utförligare beskrivning av studiens genomförande *avsedd för lekmän* kan vid behov bifogas den för fackmän avsedda obligatoriska forskningsplanen.

Vi ansöker om att genomföra en randomiserad studie för ungdomar mellan 13 och 15 års ålder med svår fetma. Ungdomarna randomiseras till optimerat medicinska omhändertagande eller till helhetsomhändertagande som även innfattar gastric bypass-operation. En förutsättning för deltagande i studien är att såväl ungdomen som vårdnadshavare kan tänka sig att acceptera kirurgisk behandling.

Syftet med studien är att värdera om det finns fördelar att genomföra fetmaoperation redan tidigt i tonåren jämfört med att vänta tills sena tonåren och har blivit myndig.

Bakgrund:

Fetma är en av de största utmaningarna för framtidens sjukvård då det kan leda till ökad risk för en rad sjukdomar, som typ 2 diabetes, hjärt-/kärlsjukdom, cancer och förtida död. Särskilt oroväckande är den den höga prevalensen av fetma bland barn och ungdomar.

Sannolikheten att en tonåring som lider av svår fetma fortsätter att ha en hög vikt i vuxen ålder är mycket hög. Det finns f n få eller inga konventionella behandlingar som visat goda resultat för svårt fetma i tonåren.

För vuxna och ungdomar från 18 års ålder är fetmakirurgi den enda behandlingen som har påvisat långsiktigt goda resultat beträffande viktnedgång (>20 år). Den bestående viktminskningen leder till lägre förekomst av kardiovaskulära riskfaktorer, minskad sjuklighet (som typ 2 diabetes, hjärtinfarkt, stroke, cancer), ökad livskvalitet och längre livslängd. ANSÖKAN OM ETIKPRÖVNING 5

År 2006 startades en nationell svensk studie - AMOS - där sammanlagt 81 ungdomar mellan 13 och 18 år opererades efter att konservativ viktbehandling misslyckats. De opererarades med laparoskopisk gastric bypass och en kontrollgrupp på 81 tonåringar som matchats för kön ålder och BMI identifierades från barnfetmaregister i Sverige (BORIS) och följdes parallellt. Tvåårsresultaten visar att viktnedgången och de metabola förbättringarna efter gastric bypass är helt jämförbara med vad som tidigare visats hos vuxna. Operationen tolererades i allmänhet väl av tonåringarna.

Vi genomför nu femårskontroller av de 81 ungdomar som genomgått gastric bypass samt den matchade kontrollgruppen som erhållit traditionell fetmabehandling samt i en vuxen grupp som opererades med gastric bypass under samma tidsperiod. Resultaten är så här långt positiva.

Förväntad nytta:

Den förväntade nyttan med operation är att ge ungdomarna en normaliserad psykosocial utveckling i den känsliga perioden i sena tonåren och unga vuxna livet. Samtidigt finns det möjlig vinst i att inte leva med riskfaktorer för metabol sjukdom under tonårstiden då ”sjukdomskartan” under livet präglas.

Den primära utfallsvariabeln är viktnedgång och viktkontroll. Sekundära utfallsvariabler innefattar kardiovaskulära riskfaktorer, utveckling av kärlförändringar, livskvalitet, ätmönster, social, ekonomisk och psykologisk utveckling.

Vi vill också värdera kirurgins effekter beträffande kroppslig utveckling, vitaminnivåer, kirurgiska bieffekter och andra potentiella negativa aspekter.

Ungdomarna i den gruppen som erbjuds konservativ terapi ska kunna erbjudas kirurgi när de blivit 18 år och myndiga eller i undantagsfall mellan 16 ch 18 år om de utvecklar allvarlig fetmarelaterad sjukdom eller på andra kliniska grunder bedömes ha indikation för operativ behandling.

2:2 Vilken/vilka vetenskaplig (a) frågeställning (ar) ligger till grund för projektets utformning?

Om projektet kan karakteriseras som en hypotesprövning, ange den primära och eventuellt sekundära hypotesen. Hänvisning till mer detaljerad information för fackmän kan ske till bifogad forskningsplan/protokoll enligt punkt 2:1.

Finns det fördelar med att erbjuda operation i nedre tonåren jämfört med att genomföra konservativt omhändertagande och vänta tills de fyllt 18 år? ANSÖKAN OM ETIKPRÖVNING 6

Är den metabola kontrollen (blodsocker, blodfett, blodtryck, inflammation mm) bättre i gruppen som genomgår tidig kirurgisk behandling bättre än de som opereras senare?

Tolereras operation väl av de som opereras tidigt i tonåren?

Kan operationen återställa livskvaliteten till motsvarande normen för åldersgruppen?

Förbättras den sociala, ekonomiska och utbildningsmässiga utvecklingen?

Är den kroppsliga/ skelettmässiga utvecklingen normal efter gastric bypass-operation i nedre tonåren?

Uppkomst av överskottshud efter viktnedgång och livskvaliteten hos ungdomar som genomgår fetmakirurgi, skillnader mellan tidiga o sena tonåren?

Kommer de som randomiseras till konventionell terapi att vilja genomgå operation i ett senare skede?

Kan sjukvårds och läkemedelskonsumtion minskas genom kirurgisk behandling?

2:3 Redogör för resultat från relevanta djurförsök (Gäller klinisk behandlingsforskning)

Om djurförsök inte utförts ange skälen till detta.

Ej relevant. Operationerna utförs redan på människa.

2:4 Redogör översiktligt för undersökningsprocedur, datainsamling och datas karaktär (Se p. 9 bil. nr 5 och p. 2:4 i Vägledning till ansökan)

Av beskrivningen ska framgå hur projektet planeras genomföras. Beskriv insamlade datas karaktär. Ange hur datas tillförlitlighet säkerställs (t.ex. kvalitetskontroll/monitorering). Vid enkäter och intervjuer ska beskrivas tillvägagångssätt och t.ex. frågors innehåll och hur slutsatser dras. Enkäter och skattningsskalor *ska* bifogas (se p. 9 bil. nr 5). För medicinsk forskning ska anges t.ex. typer av ingrepp, mätmetoder, antal besök, tidsåtgång vid varje försök, doser och administrationssätt för eventuella läkemedel och/eller isotoper, blodprovsmängd (även ackumulerad mängd vid multipla försök). Ange om och på vilket sätt undersökningsprocedur m.m. skiljer sig från klinisk rutin. Om en behandling studeras för första gången på människa ska detta framgå och relevanta säkerhetsrutiner beskrivas. Ange proceduren för att ge den eventuella behandling efter projektets slut, som kan erfordras. Ange procedur för insamling av biologiskt material. Redogör för datakällor och procedurer vid behandling av personuppgifter. För mer detaljerad information kan hänvisning ske till bilagd forskningsplan.

I denna studie planerar vi att rekrytera 13-15 åringar med svår fetma (isoBMI >35) som kan tänka sig genomgå fetmaoperation med gastric bypass. De kommer att informeras om våra hittills försiktigt positiva resultat från den tidigare studier men att vi inte vet om det är att föredra att

operera dem direkt eller att genomföra fortsatt intensiv medicinsk behandling och eventuellt operera senare.

Ungdomarna randomiseras till att antingen:

1. Genomgå gastric bypass-operation i ett helhetsomhändertagande

2. Genomföra ett medicinskt program för behandling av övervikt. Det innebär en inledande Low Calorie Diet-period följt av fortsatt livsstilsbehandling med kost- och motionsråd och ev. läkemedelsbehandling.

Genom att vi i Sverige för närvarande inte har indikation för operativ behandling av fetma under 18 år kommer kontrollerna att förbli oopererade tills de fyllt 18 år. För individer som progredierar i sin fetma på ett allvarligt sätt eller utvecklar allvarlig sjuklighet kan operation övervägas även mellan 16 och 18 års ålder.

I den kirurgiska armen utförs en laparoskopisk gastric bypass med den teknik som beskrivs av Olbers et al och använts i AMOS. De perioperativa riskerna vid gastric bypass har varit mycket låga i vår förstudie på 81 patienter (två behövde blodtransfusion och en antibiotika pga infektion). På lång sikt finns en risk att utveckla järnbristanemi och vissa andra brister på grund av minskat mineral/vitaminupptag. Ett observandum i vår förstudie att ungdomarnas compliance för supplementering av vitaminer varit dålig. Vi kommer att försluta slitsar för att minimera risken med så kallade inre bråck som ger tarmvred och ge förebyggande behandling mot gallstensbildning.

Operationerna i den randomiserade studien kommer att utföras i samarbete mellan Carlanderska stiftelsen och avdelningarna för kirurgi och pediatrik vid Sahlgrenska universitetssjukhuset i Göteborg. Öppenvårdbesök (inklusionsbesök och postoperativa uppföljningar) kommer att ske såväl vid Drottning Silvias barn och ungdomssjukhus som vid barnkliniken på Astrid Lindgrens barnsjukhus.

I den medicinska armen kommer studiedeltagarna att följas under studieperioden med intensivt multidiciplinärt omhändertagande med regelbundna besök på fetmamottagningarna vid Drottning Silvias Barn o Ungdomssjukhus samt vid Rikscentrum för barnobesitas, Karolinska sjukhuset, där bästa tillgängliga behandling mot fetma ska ges (inklusive läkemedel). ANSÖKAN OM ETIKPRÖVNING 8

En baslinjeundersökning kommer att genomföras. Det första återbesöket sker 8 veckor efter inledning av terapi. Vidare studiebesök kommer att ske vid 1, 2, 5 och 10 år samt vid identifikation av adverse events. Studiendersökningar kommer också utföras när de opererade fyller 20, 25 respektive 30 år. Utöver dessa studiebesök kommer ungdomarna också vara på ytterligare kliniska besök där samordning tidsmässigt eftersträvas i störst möjliga utsträckning.

Inklusionskriterier:

• Ålder 13-15 år

• Iso-BMI ≥ 35 kg/m2

• Genomfört minst ett års adekvat konventionell viktreducerande behandling.

• Pubertetsmognad Tanner >3

• Genomgått bedömning av psykolog

• Positiv inställning till långsiktig uppföljning.

Exklusionskriterier:

• Svårt bristande compliance

• Tidigare större bukkirurgi

• Specifikt fetmasyndrom som Prader Willis, Laurence Moon-Bardet-Biedl

• Fetma sekundärt till skador på CNS

• Pubertetsstadie Tanner ≤ 3 eller avsaknad av pubertet

• Psykotisk sjukdom

• Psykiskt handikappade

• Svår allmänsjukdom och därför olämpliga för allmän anestesi

• Leptinbrist eller homozygot MC4R defekt

Undersökningar vid studiepunkterna kommer att utföras enligt nedan:

Besöket på mottagnngen kommer att ta ca 4-5 timmar beroeende på olika individers särskilda behov ANSÖKAN OM ETIKPRÖVNING 9

Frågeformulär

För livskvalitet: RAND-36, OP skalan (obesitasspecifikt QoL)

Psykisk hälsa: Beck Youth Inventory, 5-15, ASSQ, ARSR, Rosenberg Self Esteem, Mood Adjective Check List

Fysisk aktivitet/ Ätmönster: Diet history, TFEQ-R21, QEWP-R, IPAQ

Mag-tarmfunktion: Gastro-Intestinal symptom rating scale (GSRS), Dumping Symptom Rating Scale (DSRS)

Missbruk: AUDIT o DUDIT

Enkäterna är validerade och de flesta användes i den tidigare etikgranskade studien AMOS Dnr 523-04

Blodprovsanalys

Totalt kolesterol, HDL-kolesterol, LDL-kolesterol och triglycerider och apo-lipoproteiner liksom plasma insulin o Glukos samt HbA1c kommer att analyseras Dessutom kommer vi att analysera en rad inflammatoriska markörer (supersensitivt CRP, IL-6, TNF-alfa och Adiponectin) samt analys av steroihormoner med massspektrometri. Genetiska prover för screening av kända fetmagener kommer att undersökas med snip-analyser och metyleringsstudier. Syftet med dessa undersökningar är dels att få en uppfattning om vissa genetiska profiler svarar sämre på kirurgisk terapi och dels för att kunna ge ungdomarna och deras vårdnadshavare information om det fanns en genetisk underliggande bakgrund till utvecklingen av svår fetma vilket i så fall kan vara till hjälp

Blodprovtagning kommer att insamlas med sammanlagt 50 ml extra blod vid samma tillfälle som rutinblodprovtagningen sker vid studiestart, 2, 6, 12 och 24 samt 60 månader efter studiestart. Ackumulerad mängd extra under 5 år= 250 ml

Endotelfunktion mätningar

Endotelfunktion kommer att bedömas med hjälp av reaktiv hyperemi perifer och arteriell tonometri (pulse vave velocity)

Ultraljudsmätningar med högupplösande ultraljud på 55 MHz (Visualsonics)

24 timmars blodtrycksmätning. Sömnundersökningar i hemmiljö. ANSÖKAN OM ETIKPRÖVNING 10

Utveckling av överskottshud och fysisk aktivitetsnivå och prestationsförmåga undersöks.

Hälsoekonomisk analys

Vi kommer att prospektiv insamla ackumulerade hälso och sjukvårdskostnader i båda grupperna.

Vi planerar även att följa upp opererade ungdomar och kontroller avseende potentiella biverkningar, behov av läkemedel, samt hälsoekonomiska utfall genom länkning av kliniska data till nationella register såsom Patientregistret, Läkemedelsregistret, Cancerregistret och Dödsorsaksregistret vid Socialstyrelsen, samt

Försäkringskassans register över sjukskrivning och sjuk-/aktivitetsersättning. Detta har tidigare gjorts inom den kontrollerade kliniska prövningen Swedish Obese Subjects, vilken representanter från forskargruppen varit involverade i. Migrationsdata och socioekonomiska variabler (utbildning, civilstånd, inkomst) planeras att inhämtas från Statistiska centralbyrån.

Kroppssammansättning

För kroppssammansättning kommer DEXA att användas (minimal stråldos ca 0,002mSv) Undersökningen tar ca 15 min.

Vi kommer att ta fram ett digitalt behandlingsstöd (hemsida, app) som är anpassat för ungdomsgruppen. Det digitala behandlingsstödet syftar till att dels fungera som ett sätt att öka följsamheten till det förändrade livsmönstret (främst vad gäller diet och motion) och att säkerställa informationsbehovet hos gruppen Det digitala verktyget kommer att anpassas för ungdomsgruppens speciella behov avseende språkbruk, användarvänlighet och interaktivitet.

Vi kommer också att prova ett kosttillskott, som är speciellt framtaget för att tillgodose det specifika behovet av daglig tillförsel av extra vitaminer och mineraler efter gastric bypass hos ungdomar. Tabletten ersätter andra tillskott (som visat sig ha dålig följsamhet hos tonåringar) i en och samma tablett som tas 1-3 gånger per dag. Tillskott av vitaminer och mineraler är viktigt hos gastric bypass-opererade för att undvika bristtillstånd.

2:5 Redogör för om insamlat biologiskt material kommer att förvaras i en biobank

(Se p. 2:5 i Vägledning till ansökan)

Med biobank avses biologiskt material från en eller flera människor som samlas och bevaras tills vidare eller för en bestämd tid och vars ursprung kan härledas till den eller de människor från vilka materialet härrör. Redogör för var och hur prover som ska sparas förvaras, kodningsprocedurer och villkor för utlämnande av prover. Ange huvudman för biobanken. ANSÖKAN OM ETIKPRÖVNING 11

Helblod, serum och EDTA-plasma sparas i minus 80 grader frys på Tillväxtlaboratotiet, Centrum för pediatrisk tillväxtforskning. Drottning Silvias Barn och Ungdoms sjukhus. Proverna sparas i biobank i enlighet med Socialstyrelsens föreskrifter (enl. lagen 2002;297).

Skriftligt samtycke inhämtas från forskningspersonerna innan provtagning. Blodproverna kodas på samma sätt som övrig data inom studien och enbart studieansvarig har tillgång till kodnyckeln. Villkor för utlämnande av prover innebär en etisk granskning och samtycke från vederbörande forskningspersoner.

2:6 Dokumentation, dataskydd och arkivering (Se p. 2:6 i Vägledning till ansökan)

Redogör för hur undersökningsprocedurer och eventuella ingrepp dokumenteras. Ange om band- och videoinspelningar används. Om materialet ska kodas, ange proceduren, vem som förvarar kodlistor/kodnycklar och vem eller vilka som har tillgång till dem, var och hur länge de förvaras samt om materialet kommer att anonymiseras eller förstöras. Redogör för vilken tillgänglighet datamaterialet har och hur det förvaras samt hur erforderligt sekretesskydd erhålls.

Undersökningsresultat o data registreras i ett e-CRF som ligger i en säker databas med inlogg som vi tidigare använt i AMOS. Data kommer dessutom att införas i de nationella kvalitetsregisterna BORIS- (Barnobesitasregister i Sverige) och SOReg- (Scandinavian Obesity surgery Registry) och enligt gällande regler anteckning i patientens sjukhusjournal om studiedeltagande.

I studiens sammanställning för statistisk analys föreligger data i avidentifierad kodad form. Kodnyckeln förvaras inlåst och endast koordinator och studieledare har tillgång till den.

2:7 Redogör för tidigare erfarenheter (egna och/eller andras) av den använda proceduren, tekniken eller behandlingen

Särskilt angeläget är att redovisning av risker för komplikationer görs tydliga och i förekommande fall med angivande av relevanta publikationer. Vid nya behandlingar av patienter, t.ex. med läkemedel, bör anges hur många patienter (med aktuell eller annan åkomma) som tidigare erhållit föreslagen behandling, läkemedelsdosering (eller annan dosering) samt hur långa behandlingsperioder som studerats. ANSÖKAN OM ETIKPRÖVNING 12

Under 2009 publicerades i JAMA en randomiserad studie från Melbourne, Australien där operation med s k laparoskopisk gastric banding ledde till bättre tvåårsutfall än konservativ behandling.

Flera andra mindre serier med operationer av ungdomar med olika operationsmetoder har visat att det förefaller säkert och allmänt sett förenat med positivt utfall att operera tonåringar.

År 2006 startade vi i Sverige AMOS (Adolescent Morbid Obesity Surgery study) där vi valde att starta en icke randomiserad jämförande studie då tillräckliga säkerhets och långtidsdata saknades. Sammanlagt 81 ungdomar mellan 13 och 18 år, som misslyckats med konservativ viktbehandling, har operarats med laparoskopisk gastric bypass samtidigt som en kontrollgrupp på 81 tonåringar som matchats för kön ålder och BMI identifierades i barnfetmaregister i Sverige (BORIS).

Flera artiklar har publicerats från AMOS med 100 % uppföljning. Vi beskriver att gruppen som helhet tolererade operationen väl och förbättrades i självbild och livskvalitet medan en mindre grupp behövde mer psykologiskt stöd. Vi visar också att den opererade gruppen reducerar vikten lika mycket som en jämförande vuxen kontrollgrupp och att påtagliga och breda förbättringar i kardiovaskulära riskfaktorer och även stora förbättringar beträffande både psykisk och fysisk livskavlitet.

3. Uppgifter om forskningspersoner

3:1 Hur görs urvalet av forskningspersoner? (Se p. 9 bil. nr 3 och p. 3:1 i Vägledning till ansökan)

Med forskningsperson avses en levande människa som forskningen avser. Ange urvalskriterier (inklusion och exklusion). Redogör för på vilket sätt forskaren kommer i kontakt med/får kännedom om lämpliga forskningspersoner. Om annonsering sker, *ska* annonsmaterialet insändas som bilaga (se p. 9 bil. nr 3). Om t.ex. barn eller personer som tillfälligt eller permanent inte är kapabla att ge ett eget informerat samtycke ska ingå i

projektet, ska detta särskilt motiveras. Om vissa grupper utesluts från deltagande i projektet ska detta särskilt motiveras.

Ungdomarna kommer att rekryteras genom att de högspecialiserade centra som ingår i studien och behandlar barn med svår fetma (Unviersitetssjukhuset i Malmö, Drottning Silvias Barn- och Ungdomssjukhus i Göteborg och Rikscentrum barnobesitas, Astrid Lindgrens Barnsjukhus i Stockholm) i sin ordinarie verksamhet identifierar och tillfrågar ungdomar med iso-BMI > 35 om att delta i studien. Barnfetmaläkaren värderar om ungdomarna har fått gängse tillgänglig behandling för fetma och att ungdomarna gjort allvarligt menade försök till viktreduktion.
ANSÖKAN OM ETIKPRÖVNING 13

Barnfetmaläkaren utesluter också kontraindikationer och ger ungdomarna information om möjligheten att gå med i den planerade studien och vad den innebär.

De 13-15 åringar som uppfyller kriterierna för att delta i den randomiserade studien kommer att tillfrågas om deltagande.

3:2 Ange relationen mellan forskare/försöksledare och forskningspersonerna

Behandlare (t.ex. läkare, psykolog, sjukgymnast) - forskningsperson (t.ex. patient, klient)

Kursgivare (lärare) - student

Arbetsgivare - anställd

Annan relation som kan tänkas medföra risk för påverkan. Beskriv:

3:3 Redogör för det statistiska underlaget för studiepopulationens (-ernas)/ undersökningsmaterialets (-ens) storlek (Se p. 3:3 i Vägledning till ansökan)

Redovisa statistisk styrka, så kallad ”power”- beräkning eller redovisa motsvarande överväganden som tydliggör studiens möjligheter att besvara frågeställningarna.

Viktutvecklingen (beräknat som bestående reduktion i BMI) är den primära utfallsvariabeln. Dock värderar vi det som jämförbart viktigt att förutom vikten studera utfallet för sekundära utfall som livskvalitet, psykosocial utveckling, kardiovaskulära riskfaktorer, strukturella kardiovaskulära förändringar och belastningsproblem.

Vi har god power beträffande den primära utfallsvariabeln (BMI-utveckling) för att nå relevant statistisk signifikans (>95% på 0,01 nivå) redan med 10 ungdomar i varje grupp. För att värdera sekundära utfallsvariabler, som metabola och kardiovaskulära data, beräknar vi att 25 patienter i varje grupp är tillräcklig för att uppnå 80 % power på alfa på 0,05 på grundval av vår förstudie av 81 patienter. Beträffande ungdomarnas egna upplevda hälsa så visar vår studie att en stor majoritet av våra patienter får ökad livskvalitet och att 25 individer i varje arm ger god power för skillnader på 0,05 nivån. Genom att sikta på att inkludera 60 ungdomar (30+30) får vi en säkerhet för att även med 10-20 % bortfall ha tillräcklig statisktisk styrka

3:4 Kan forskningspersonerna komma att inkluderas i flera studier samtidigt eller i nära anslutning till denna? I så fall, vilken typ av forskning?

(Se p. 3:4 i Vägledning till ansökan)

Inget planerat

3:5 Vilket försäkringsskydd finns för de forskningspersoner som deltar i projektet? ANSÖKAN OM ETIKPRÖVNING 14

Det åligger forskningshuvudmannen att kontrollera att det finns försäkring som täcker eventuella skador som kan uppkomma i samband med forskningen.

Patientförsäkringen gäller.

3:6 Vilken ekonomisk ersättning eller andra förmåner utgår till de forskningspersoner som deltar i projektet och när betalas ersättningen ut? Utförligare beskrivning kan lämnas i bilaga. (Se p. 9 bil. nr 11 och p. 3:6 i Vägledning till ansökan)

Ersättning för obehag och besvär. Ange belopp (före skatt): Nej

Ersättning för förlorad arbetsinkomst Ja Nej

Reseersättning Ja Nej

Befrielse från kostnader för läkemedel Ja Nej

Befrielse från andra kostnader. Vilka? Nej

Andra förmåner. Vilka? Nej

När betalas ersättningen ut?

Ingen ersättning betalas ut

4. Information och samtycke

4:1 Proceduren för och innehållet i den *information* som lämnas då forskningspersoner tillfrågas om deltagande

(Se p. 9 bil. nr 4 och Vägledning till forskningspersonsinformation.)

Enligt 16 § lag (2003:460) om etikprövning av forskning som avser människor ska forskningspersonen informeras om den övergripande planen för forskningen, syftet med forskningen, de metoder som kommer att användas, de följder och risker som forskningen kan medföra, vem som är forskningshuvudman, att deltagande i forskningen är frivilligt och forskningspersonernas rätt att när som helst avbryta sin medverkan. Beskriv hur och när information ges och vad den innehåller. Ange vem som informerar. Normalt ska en kortfattad och lättförståelig skriftlig information ges. Denna skriftliga information *ska* bifogas ansökan (se p. 9 bil. nr 4). Om ingen eller ofullständig information ges, måste skälen för detta noggrant anges.

Barnfetmaläkare kommer att se till att ungdomarna har fått eller får gängse tillgänglig rådgivning för fetma och att de gjort allvarligt menade viktreduktionsförsök, samt ge dem information både muntligt och skriftligt om

möjligheten att gå med i den planerade studien. Om intresse finns för deltagande i studien ges specifik information och utvärdering av obesitaskirurg och övrig barnmedicinsk personal i form av barnläkare, barnsjuksköterska, psykolog och dietist. ANSÖKAN OM ETIKPRÖVNING 15

Efter att under god tid fått fundera igenom informationen bereds ungdomen och dess vårdnashavare möjlighet att ånyo ställa frågor till såväl barnmedicinsk som kirurgiskt erfarna personer.

Den skriftliga informationen lämnas i två varianter; en som är anpassad för ungdomar och en för deras vårdnadshavare.

4:2 Hur och från vem inhämtas *samtycke*? (Se Vägledning till forskningspersonsinformation)

Beskriv proceduren; vem som frågar, när detta sker och hur samtycket dokumenteras. Utförlig redovisning är särskilt viktig då barn eller personer med nedsatt beslutskompetens ingår i studien, likaså vid studier av en grupp/grupper, t.ex. skolklasser, föreningar, organisationer, företag, kyrkosamfund, församlingar eller grupper som interagerar inom sociala medier.

Skriftligt samtycke inhämtas från såväl ungdom som vårdnashavare

5. Forskningsetiska överväganden

5:1 Redogör för alla risker som deltagandet kan medföra

Dessa kan vara t.ex. fysisk eller psykisk skada, smärta, obehag eller integritetsintrång på kort eller lång sikt. Ange vilka åtgärder som har vidtagits för att förebygga riskerna som nämns ovan samt vilken beredskap som finns för att hantera sådana komplikationer. Ange vilka/de metoder som kommer att användas för att efterforska, registrera och rapportera oönskade händelser.

Trots att vi har goda säkerhetsdata från en studie av operationer av ungdomar under fem år (AMOS studien) kan det fortfarande vara så att det inte för alla är fördelaktigt att opereras i åldern under 18 år. Vi vet framför allt inte om det är fördelaktigt att operera i de nedre tonåren. Komplikationer och sidoeffekter av operation kan leda till besvär.

Det finns i den randomiserade studien inte möjlighet att välja kirurgisk behandling och hälften av de som ingår i studien kommer att fortsätta genomgå konservativ behandling vilket kan uppfattas som en besvikelse efter att de kunnat tänka sig operation. Genom att erbjuda en intensifierad behandling i den konventionella armen vill vi minimera känslan av att uppfatta den som det "sämre" alternativet.

En förlängning av ordinarie mottagningsbesök i anslutning till behandlingarna kommer att tas i anspråk för ifyllande av enkäter och extra undersökningar. En begränsad mängd blod (50 ml) extra kommer att insamlas vid varje ordinarie blodprovtagningstilfälle.

Några av de tilltänka enkäterna kan upplevas påträngande och det finns en risk för att ungdomarna upplever det jobbigt att beskriva sin situation. Alla enkäter fylls i tillsammans med utbildad personal som också har erfarenhet att ta ett fortsatt ansvar för vidare omhändertagande ANSÖKAN OM ETIKPRÖVNING 16

om behovet uppstår. Ett flertal av enkäterna används inom den kliniska verksamheten för konventionell behandling av överviktiga ungdomar idag och personalen har därmed en bred erfarenhet av enkäterna och vad dess utfall kan innebära.

Alla registerdata samlas in rutinmässigt, så försökspersonerna utsätts inte för några ytterligare risker eller tidskrävande undersökningar. Samtidigt kan viktiga data över exempelvis biverkningar och hälsoekonomiska variabler samlas in, vilket är svårt att göra med full täckning på andra sätt. Således är riskerna i stort sett obefintliga medan det finns en stor nytta med att kombinera kliniska data med registerdata. Dock aktualiseras integritetsaspekten, eftersom några studiedeltagare skulle kunna uppleva detta som ett integritetsintrång. För att undanröja denna risk för upplevt integritetsintrång krävs informerat samtycke. Vidare kommer resultat enbart presenteras på gruppnivå så att enskilda individer inte kan spåras

5:2 Redogör för möjlig nytta för de forskningspersoner som ingår i projektet (gäller särskilt behandlingsforskning)

Det mesta talar för att de positiva effekter som kan uppnås hos vuxna som genomgår kirurgi också kan erhållas vid operation av ungdomar under 18 år. Vi vill därför ge de ungdomar mellan 13 och 15 år med synnerligen allvarlig fetma och medicinska komplikationer en möjlighet att genomgå operation, dock i fortsatt studieform. Vi anser oss ha ett ansvar att genomföra den randomiserade studie som i så fall kan visa att kirurgisk behandling är att rekommendera även för tonåringar.

Hälften av de som ingår i den randomiserade studien får möjligheten att genomgå operation och dess positiva konsekvenser medan den konservativt behandlade gruppen erbjuds en behandling som i regel är intensivare än gängse praxis för ungdomar.

Målet är att täcka den kunskapslucka som för närvarande föreligger inom sjukvården om individer under 18 års ålder med svår fetma kan återföras till ett normaliserat livslopp genom att genomföra en visserligen radikal men livsförändrande operation. Om så är fallet kommer en tidigare mycket utsatt grupp i samhället med mycket hög förväntad sjuklighet, låg livskvalitet och kraftigt reducerad förväntad livslängd kunna erbjudas en effektiv och livsförbättrande behandling. Även ur ett samhällsperspektiv kommer kostnader kunna reduceras genom minskad sjuklighet, sjukfrånvaro mm som uppstår till följd av fetman.

5:3 Identifiera och precisera om eventuella etiska problem (fördelar/nackdelar) kan uppstå i ett vidare perspektiv genom projektet (Se p. 5:3 i Vägledning till ansökan)

Här kan redovisas om exempelvis vissa grupper (andra än de forskningspersoner som ingår i forskningsprojektet) kan komma att utpekas/få hjälp som ett resultat av studien. ANSÖKAN OM ETIKPRÖVNING 17

Det etiska dilemmat kan vara att ungdomar med begränsad autonomi och svårighet att överblicka livslånga konsekvenser av sitt val att genomgå kirurgisk behandling. Å andra sidan är det etiskt tveksamt att inte kunna

erbjuda denna potentiellt livförbättrande behandling till ungdomari den rådande bristen på alternativa effektiva terapier.

I Socialstyrelsens register samlas rutinmässigt in viktiga data över exempelvis biverkningar och hälsoekonomiska variabler, vilket är svårt att göra med full täckning på andra sätt. Riskerna är att i stort sett obefintliga medan det finns en stor nytta med att kombinera kliniska data med registerdata. För att undanröja risk för upplevt integritetsintrång krävs informerat samtycke. Vidare kommer resultat enbart presenteras på gruppnivå så att enskilda individer inte kan spåras.

Vår sammantagna bedömning är att det vore oetiskt att inte på detta sätt fortsätta att systematiskt studera värdet av kirurgisk behandling för ungdomar med svår fetma.

6. Redovisning av resultaten

6:1 Hur garanteras forskningshuvudmannen och medverkande forskare tillgång till data (anges vid t.ex. uppdragsforskning) och vem ansvarar för databearbetning och rapportskrivning? (Se p. 6:1 i Vägledning till ansökan)

Data hanteras av forskningshuvudmannen. Studieansvarige läkare Torsten Olbers som ansvarar för databearbetning och rapportskrivning

6:2 Hur kommer resultaten att göras offentligt tillgängliga? Kommer studien att insändas för publicering i tidskrift eller publiceras på annat sätt? (Se p. 6:2 i Vägledning till ansökan)

Ange i vilken form resultaten planeras offentliggöras samt tidsplan för detta.

Data kommer att publiceras som artiklar i internationella tidsskrifter och vid internationella konferenser.

6:3 På vilket sätt garanteras forskningspersonernas rätt till integritet när materialet offentliggörs/publiceras?

Redovisas resultat på statistisk gruppnivå? Beskriv procedurer eller metoder för avidentifiering/anonymisering.

All data från studien presenteras i avidentifierad form. Inga data som kan härledas till ensikild person får presenteras utan att man i så fall uttryckligen inhämtat tillstånd från vederbörande och dess vårdnadshavande.

7. Redovisning av ekonomiska förhållanden och beroendeförhållanden

Redovisning enligt punkterna 7:1-7:3 syftar till att tydliggöra alla direkta eller indirekta förhållanden, som kan tänkas påverka forskarens relation till forskningspersonerna (vid t.ex. informations-, samtyckes-, genomförandeprocedurer).

7:1 Vid uppdragsforskning

Ange uppdragsgivaren t.ex. ett företag (vid klinisk läkemedelsprövning eller prövning av andra nya produkter), en organisation eller en myndighet.

Namn: Kontaktperson:

Adress: Telefon/mobiltelefon:

Ange uppdragsgivarens relation till forskningshuvudmannen/medverkande forskare, t.ex. anställningsförhållande

Anställningsförhållande i offentlig sjukvård eller universitet.

7:2 Redovisa eventuella ekonomiska överenskommelser med uppdragsgivare eller

andra finansiärer (namn, belopp)

Vid klinisk läkemedelsprövning bör hänvisning ske till ingånget avtal med sjukvårdshuvudmannen. Liknande överenskommelser kan förekomma vid annan uppdragsforskning och bör redovisas på samma sätt. Separata överenskommelser med den/de som ska genomföra forskningen ska redovisas. Belopp som kommer att erhållas för studien/ersättning till kliniken/genomföraren, vad ersättningen bör täcka och ev. belopp som erhålls per forskningsperson, bör också anges här.

Sammantaget löfte om medfinansiering under tre år:

Brainbridge 97 tkr

Kommer att utveckla en smartphone-app för interaktivitet, information och positiv reinforcement

Johnson&Johnson 80tkr

Utbildning av personal i obesitaskirurgiskt omhändertagande.

7:3 Redovisa forskningshuvudmannens, huvudansvarig forskares och medverkande forskares egna intressen ANSÖKAN OM ETIKPRÖVNING 19

Här redovisas t.ex. aktieinnehav, anställning, konsultuppdrag i finansierande företag, eget företag som kan få (direkt eller indirekt) ekonomisk vinst av forskningen.

Inga relevanta

8. Undertecknande

Behörig företrädare för sökande forskningshuvudman enligt p. 1:2.

Ort: Göteborg Datum:

Signatur: ____

Namnförtydligande: Ralph Bågenholm

Tjänstetitel: Verksamhetschef, överläkare

Undertecknad forskare som genomför projektet (kontaktperson) enligt p. 1:3 intygar härmed att forskningen kommer att genomföras i enlighet med ansökan.

Ort: Göteborg Datum:

Signatur: ____

Namnförtydligande: Torsten Olbers

Tjänstetitel: Överläkare ANSÖKAN OM ETIKPRÖVNING 20

9. Förteckning över bilagor (Se p. 9 i Vägledning till ansökan)

Dokument som, i tillämpliga fall, ska bifogas *om inte motsvarande information finns i blanketten* har markerats med x. Markera de bilagor som skickas in med denna ansökan. **Insänd med ansökan**	**Bil nr**	**Beskrivning**	**Klinisk läkemedels-prövning**	**Annan forskning**
1		Deltagande forskningshuvudmän och medverkande forskare (kontaktpersoner) vid forskning där mer än en forskningshuvudman deltar. Se p. 1:4	x	x
2		För fackmän avsedd forskningsplan, vid behov även för lekmän avsedd bilaga. Se p. 2:1 och Vägledning till forskningsplan/forskningsprotokoll (program)	x	x
3		Annonsmaterial för rekrytering av forskningspersoner. Se p.	x	x

	3:1 och i Vägledning till ansökan p. 3:1			
4	Skriftlig information till dem som tillfrågas. Se p. 4:1 och Vägledning till forskningspersonsinformation och (i förekommande fall) separat samtyckesformulär	x		x
5	Enkät, frågeformulär. Se p. 2:4	x		x
6	Gemensam EU blankett (gäller fr.o.m. den 1 maj 2004), gäller även vid ändring. För information se Läkemedelsverkets hemsida, www.lakemedelsverket.se		x	
7	Sammanfattning av protokollet på svenska		x	
8	Prövarhandbok alt. bipacksedel/produktresumé/IB		x	
9	Intyg från verksamhetschef/motsv. om resurser för forskningspersonernas säkerhet. Se p. 1:5 och förslag till utformning av resursintyg i Vägledning till ansökan p. 1:5	x		x
10	CV för forskare (samma som p. 1:3) med huvudansvar för genomförandet, redovisa forskarens (- arnas) kompetens av relevans för studien. Se	x		x

Vägledning till ansökan

p. 1:3

11	Beskrivning av ersättning till forskningspersoner. Se p. 3:6 och i Vägledning till ansökan p. 3:6	x	x

Translated original research plan

Project plan AMOS2, 12th of Dec 2013

Randomized trial between surgical and medical treatment for adolescents between 13 and 15 years of age with severe obesity

Scientific question

The primary objective is to provide an answer to the question of whether, and if so to what extent, young people under the age of 18 with severe obesity should be offered bariatric surgery.

The ”primary endpoint" is the reduction in BMI. Secondary study variables are quality of life, metabolic health, weight-bearing problems such as joint problems, eating patterns, cardiovascular structure, health economics.

• Are there advantages to offering surgery in the lower teens compared to carrying out conservative management and waiting until they turn 18?

• Is the metabolic control (blood sugar, blood fat, blood pressure, inflammation, etc.) better in the group that undergoes early surgical treatment than those who are operated on later?

• Is surgery well tolerated by those who have surgery early in their teens?

• Can the operation restore the quality of life to the corresponding norm for the age group?

• Is the social, economic, and educational development improving?

• Is the physical/skeletal development normal after gastric bypass surgery in the lower teens?

• How is the experience of excess skin after weight loss and the quality of life in young people undergoing bariatric surgery? Differences between early and late adolescence?

• Will those randomized to conventional therapy want to undergo surgery at a later stage?

• Can healthcare consumption and medications be reduced through surgical treatment?

Research area overview

Obesity is one of the biggest challenges for future healthcare as it leads to an increased risk of several diseases, such as type 2 diabetes, cardiovascular disease and cancer and premature death. Of particular concern is the high prevalence of obesity among children and adolescents.

The likelihood that an adolescent suffering from severe obesity will continue to be so in adulthood is very high (Fontaine, Redden et al. 2003, Holdstock, Engstrom et al. 2003, Baker, Olsen et al. 2007, Biro and Wien 2010). There are currently few or no conservative treatments that have shown good results for severe obesity in adolescence (Danielsson P 2011).

For adults and adolescents from the age of 18, bariatric surgery is the only treatment that can demonstrate long-term good results regarding weight loss (>20 years). The long-term weight loss leads to a lower incidence of cardiovascular risk factors, reduced morbidity (e.g. diabetes and cancer), increased quality of life and longer lifespan (Sjostrom, Peltonen et al. 2012).

During the last decade, gastric bypass has been established as standard treatment in obesity surgery in Sweden because it provides superior weight loss and weight stability compared to "simpler" surgical methods (such as

gastric banding) in comparative studies. In addition, gastric bypass is in most cases associated with very high "eating quality", i.e., that most people feel that they can eat in a normal way, but with a changed dietary composition, by feeling less hunger and faster satiety.

Case studies of surgical treatment of teenagers have shown positive results. However, there is only one randomized study in which laparoscopic gastric banding was found to be superior to conservative treatment for teenagers with BMI>35 in a 2-year perspective (O'Brien, Sawyer et al. 2010). Other studies using gastric banding for teenagers have shown worse results, and a study at Huddinge Hospital in the early 2000s was discontinued after a small number of operations with poor results since several complications arose.

Our project is focused on evaluating whether bariatric surgery should be offered already in adolescence.

In 2006, a national Swedish study - AMOS - was started, where a total of 81 young people between the ages of 13 and 18 underwent surgery after conservative weight management had failed. They were operated on with laparoscopic gastric bypass (Fig 1). while a control group of 81 teenagers matched for sex, age and BMI was identified in the childhood obesity register in Sweden (BORIS). The two-year results show that the weight loss and metabolic improvements after gastric bypass are completely comparable to what was previously shown in adults (Olbers, Gronowitz et al. 2012) (Jarvholm, Olbers et al 2012.).

We are now conducting five-year follow-ups of the 81 young people who underwent gastric bypass as well as the matched control group who received traditional obesity treatment and in an adult group who underwent gastric bypass surgery during the same time period.

The expected benefit of surgery is to provide the youth with a normalized psychosocial development in the sensitive period of late adolescence and young adult life. At the same time, there is a possible profit in not living with risk factors for metabolic disease during adolescence when the "disease map" during life is imprinted. We also need to evaluate the effects of surgery in terms of physical development, vitamin levels, surgical side effects and other potential negative aspects.

Project description

We plan to perform a trial in which young people between 13 and 16 years of age randomized to best medical care (including an initial low calorie diet treatment) or gastric bypass surgery. The prerequisite is that both the young person and guardian can imagine accepting surgical treatment. The youth are randomized to either undergo gastric bypass surgery directly or traditional conservative therapy, the youth in this group will be offered surgery when they reach the age of 18, if desired.

Inclusion criteria:

• Age 13-15 years

• Iso-BMI ≥ 35 kg/m2

• Completed at least one year of adequate conventional weight-reducing treatment.

• Puberty maturity Tanner >3

• Passed assessment by a psychologist

• Positive attitude to long-term follow-up.

Exclusion criteria:

• Severe lack of compliance

• Previous major abdominal surgery

• Specific obesity syndrome such as Prader Willis, Laurence Moon-Bardet-Biedl

• Obesity secondary to damage to the CNS

• Puberty stage Tanner ≤ 3 or lack of puberty

• Psychotic illness

• Mentally handicapped

• Severe general illness and therefore unsuitable for general anaesthesia

• Leptin deficiency or homozygous MC4R defect

Medical treatment of obesity

This means an initial Low Calorie Diet period of 8 weeks followed by continued lifestyle treatment with diet and exercise advice and possibly drug treatment. In the medical arm, the study participants will be followed during the study period with intensive multidisciplinary care with regular visits to the obesity clinics at Drottning Silvias Barn o Ungdomssjukhus and at the National Centre for Childhood Obesity, Karolinska Hospital, where the best available treatment for obesity will be given, diet and activity advice through motivational talks as well as including medications.

Bariatric surgery

The operation is performed by surgeons with extensive experience in obesity operations, especially keyhole surgery. During the operation, most of the stomach is bypassed by pulling up the small intestine and connecting it to a small pocket created by the upper part of the stomach (so-called Gastric Bypass). No part of the stomach or intestine is removed, but simply rewired in a new way (see picture). The operation entails a reduced opportunity to eat large portions of food. You are also "directed" to eat more healthily by feeling bad after the procedure from eating fatty or very sweet food. The portion sizes gradually become larger, and the operated person eats quite "normally" about two years after the operation when the weight loss has stopped. Most often, the individual has then reached a weight that corresponds to being modestly overweight.

The operations are performed using the keyhole technique, which means that you operate through five small holes in the abdominal wall. The operations will be performed in collaboration between the Carlanderska Foundation and the departments of surgery and paediatrics at Sahlgrenska University Hospital in Gothenburg. Outpatient visits (inclusion visits and postoperative follow-ups) will take place both at Queen Silvia's children's and youth hospital and at the children's clinic at Astrid Lindgren's children's hospital.

The investigations

A comprehensive baseline examination will be carried out before the start of treatment. Further study visits will take place at 1, 2, 5 and 10 years and when adverse events are identified. Study examinations will also be carried out when the operated persons turn 20, 25 and 30 respectively. In addition to these study visits, the young people

will also be on additional clinical visits where coordination in terms of time is sought to the greatest extent possible (see flow chart, appendix 1).

Examinations at the study points will be carried out as below:

The visit to the reception will take about 4-5 hours depending on the special needs of different individuals

‘

Questionnaires:

For quality of life: RAND-36, OP scale (obesity-specific QoL)

Mental health: Beck Youth Inventory, 5-15, ASSQ, ARSR, Rosenberg Self Esteem, Mood Adjective Check List

Physical activity/ Eating patterns: Diet history, TFEQ-R21, QEWP-R, IPAQ

Gastrointestinal function: Gastro-Intestinal symptom rating scale (GSRS), Dumping Symptom Rating Scale (DSRS)

Abuse: AUDIT o DUDIT

Blood sample analysis

Total cholesterol

HDL cholesterol

LDL cholesterol

Triglycerides

Apo-lipoproteins

Plasma insulin

Plasma Glucose

HbA1c will

In addition, we will analyse a range of inflammatory markers (supersensitive CRP, IL-6, TNF-alpha and Adiponectin) and analysis of steroid hormones with mass spectrometry.

Genetic samples for screening known obesity genes will be investigated with snip analyses and methylation studies. The purpose of these investigations is partly to get an idea of whether certain genetic profiles respond less well to surgical therapy and partly to be able to give the young people and their guardians information if there was a genetic underlying background to the development of severe obesity, which in that case could be helpful. Blood sampling will be collected with a total of 50 ml of extra blood at the same time as the routine blood sampling at the start of the study, 2, 12 and 24 and 60 months after the start of the study. Accumulated amount extra over 5 years = 250 ml

• Endothelial function measurements- Endothelial function will be assessed using reactive hyperaemia peripheral and arterial tonometry (pulse wave velocity)

• Ultrasound measurements with high-resolution ultrasound at 55 MHz (Visualsonics)

• 24-hour blood pressure measurement. Sleep examinations in the home environment.

• Development of excess skin and physical activity level and performance are examined.

• Health economic analysis

o We will prospectively collect accumulated healthcare costs in both groups.

o We also plan to follow up on operated young people and controls regarding potential side effects, need for medication, and health economic outcomes by linking clinical data to national registers such as the Patient Register, the Medicines Register, the Cancer Register and the Cause of Death Register at the National Board of Health and Welfare, as well as the Social Insurance Agency's register of sick leave and sick/ activity compensation. This has previously been done within the controlled clinical trial Swedish Obese Subjects (SOS study), in which representatives from the research group have been involved. Migration data and socio-economic variables (education, marital status, income) are planned to be obtained from Statistics Sweden.

• Body composition - For body composition DEXA will be used (minimum radiation dose approx. 0.002mSv) the examination takes approx. 15 min.

• We will develop a digital treatment support (website, app) that is adapted for the youth group. The digital treatment support aims to function as a way to increase adherence to the changed lifestyle (mainly regarding diet and exercise), to ensure the information needs of the group and an opportunity for communication between young people and the research group. The digital tool will be adapted to the youth group's special needs regarding language use, user-friendliness and interactivity.

• We will also try a food supplement, which is specially designed to meet the specific need for daily supply of extra vitamins and minerals after gastric bypass in young people. The tablet replaces other supplements (which have been shown to have poor adherence in teenagers) in one and the same tablet taken 1-3 times per day. Supplementation of vitamins and minerals is important in gastric bypass patients to avoid deficiency conditions.

Importance

Most evidence suggests that the positive effects that can be achieved in adults undergoing surgery can also be achieved in the operation of young people under the age of 18. We therefore want to give the young people between 13 and 15 years of age with particularly severe obesity and medical complications an opportunity to undergo surgery, however in the form of a continuing study. We consider ourselves to have a responsibility to carry out the randomized study which, in that case, can show that surgical treatment is also recommended for teenagers.

Half of those included in the randomized study will undergo surgery and its consequences, while the conservatively treated group is offered a treatment that more intensive than usually provided in practice for young people with obesity in Sweden.

The goal is to cover the knowledge gap that currently exists within healthcare if individuals under the age of 18 with severe obesity can be returned to a normalized life course by carrying out an admittedly radical but life-changing operation. If this is the case, a previously very vulnerable group in society with very high expected morbidity, low quality of life and greatly reduced life expectancy will be able to be offered effective and life-enhancing treatment. Even from a societal perspective, costs will be able to be reduced through reduced morbidity, sick leave etcetera that are results of living with severe obesity.

References:

Baker, J. L., et al. (2007). "Childhood body-mass index and the risk of coronary heart disease in adulthood." N Engl J Med 357(23): 2329-2337.

Biro, F. M. and M. Wien (2010). "Childhood obesity and adult morbidities." Am J Clin Nutr 91(5): 1499S-1505S.

Danielsson P, S. V., Kowalski J, Nyberg G, Ekblom Ö and Marcus C (2011). "Importance of age for three-year continuous behavioral obesity treatment success and dropout rate." Obesity facts.

Fontaine, K. R., et al. (2003). "Years of life lost due to obesity." JAMA 289(2): 187-193.

Jarvholm, K., et al. (2012) "Short-term psychological outcomes in severely obese adolescents after bariatric surgery." Obesity (Silver Spring) 2012(2): 318-323.

O'Brien, P. E., et al. (2010). "Laparoscopic adjustable gastric banding in severely obese adolescents: a randomized trial." JAMA 303(6): 519-526.

Olbers, T., et al. (2012). "Two-year outcome of laparoscopic Roux-en-Y gastric bypass in adolescents with severe obesity: results from a Swedish Nationwide Study (AMOS)." Int J Obes (Lond) 36(11): 1388-1395.

Sjostrom, L., et al. (2012). "Bariatric surgery and long-term cardiovascular events." JAMA 307(1): 56-65.

Original research plan
Forskningsplan

Randomiserad studie mellan kirurgisk och medicinsk behandling för ungdomar mellan 13 och 15 år med svår fetma

Vetenskaplig frågeställning

Primära målsättningen är att ge svar på frågan om, och i så fall i vilken utsträckning, ungdomar under 18 år med svår fetma skall erbjudas obesitaskirurgi.

Studiernas ”primary endpoint” är reduktion i BMI. Sekundära studievariabler är livskvalitet, metabol hälsa, viktbärande problem såsom ledbesvär, ätmönster, kardiovaskulär struktur, hälsoekonomi.

- Finns det fördelar med att erbjuda operation i nedre tonåren jämfört med att genomföra konservativt omhändertagande och vänta tills de fyllt 18 år?
- Är den metabola kontrollen (blodsocker, blodfett, blodtryck, inflammation mm) bättre i gruppen som genomgår tidig kirurgisk behandling bättre än de som opereras senare?
- Tolereras operation väl av de som opereras tidigt i tonåren?
- Kan operationen återställa livskvaliteten till motsvarande normen för åldersgruppen?
- Förbättras den sociala, ekonomiska och utbildningsmässiga utvecklingen?
- Är den kroppsliga/ skelettmässiga utvecklingen normal efter gastric bypass-operation i nedre tonåren?
- Uppkomst av överskottshud efter viktnedgång och livskvaliteten hos ungdomar som genomgår fetmakirurgi, skillnader mellan tidiga o sena tonåren?
- Kommer de som randomiseras till konventionell terapi att vilja genomgå operation i ett senare skede?
- Kan sjukvårds och läkemedelskonsumtion minskas genom kirurgisk behandling?

Områdesöversikt

Fetma är en av de största utmaningarna för framtidens sjukvård då det leder till ökad risken för en rad sjukdomar, som typ 2 diabetes, hjärt-/kärlsjukdom och cancer och förtida död. Särskilt oroväckande är den den höga prevalensen av fetma bland barn och ungdomar.

Sannolikheten att en tonåring som lider av svår fetma fortsätter att vara det i vuxen ålder är mycket hög (Fontaine, Redden et al. 2003, Holdstock, Engstrom et al. 2003, Baker, Olsen et al. 2007, Biro and Wien 2010). Det finns f n få eller inga konservativa behandlingar som visat goda resultat för svårt fetma i tonåren (Danielsson P 2011).

För vuxna och ungdomar ner till 18 års ålder är fetmakirurgi den enda behandlingen som kan påvisa långsiktigt goda resultat beträffande viktnedgång (>20 år). Den bestående viktminskningen leder till lägre förekomst av kardiovaskulära riskfaktorer, minskad sjuklighet (t ex diabetes och cancer), ökad livskvalitet och längre livslängd (Sjostrom, Peltonen et al. 2012).

Under senaste decenniet har gastric bypass etablerats som standardbehandling inom obesitaskirurgin i Sverige eftersom den ger överlägsen viktnedgång och viktstabilitet jämfört med ”enklare” operationsmetoder (som gastric banding) i jämförande studier. Dessutom, är gastric bypass i de allra fall är förknippat med mycket hög ”ätkvalitet”, d v s att de flesta upplever att de kan äta på ett normalt sätt, men med ändrad kostsammansättning, genom att de känner mindre hunger och snabbare mättnad.

Fallstudier med kirurgisk behandling av tonåringar har visat positiva resultat. Dock finns det bara en randomiserad studie där laparoskopisk gastric banding visade sig vara överlägsen konservativ behandling för tonåringar med BMI>35 i ett 2-årsperspektiv (O'Brien, Sawyer et al. 2010). Andra studier med användande av

gastric banding för tonåringar har visat sämre resultat, och en studie på Huddinge sjukhus under tidigt 2000-tal avbröts efter ett mindre antal operationer med dåliga resultat sedan flera komplikationer uppstått.

Vårt projekt är inriktat på att utvärdera om fetmaoperation ska erbjudas redan i tonåren.

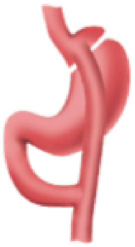
Fig 1: Gastric bypass

År 2006 startades startades en nationell svensk studie - AMOS - där sammanlagt 81 ungdomar mellan 13 och 18 år opererades efter att konservativ viktbehandling misslyckats. De opererarades med laparoskopisk gastric bypass (Fig 1). samtidigt som en kontrollgrupp på 81 tonåringar som matchats för kön ålder och BMI identifierades i barnfetmaregister i Sverige (BORIS). Tvåårsresultaten visar att den viktnedgången och de metabola förbättringarna efter gastric bypass är helt jämförbara med vad som tidigare visats hos vuxna (Olbers, Gronowitz et al. 2012) (Jarvholm, Olbers et al 2012.).

Vi genomför nu femårskontroller av de 81 ungdomar som genomgått gastric bypass samt den matchade kontrollgruppen som erhållit traditionell fetmabehandling och i en vuxen grupp som opererades med gastric bypass under samma tidsperiod.

Den förväntade nyttan med operation är att ge ungdomarna en normaliserad psykosocial utveckling i den känsliga perioden i sena tonåren och unga vuxna livet. Samtidigt finns det möjlig vinst i att inte leva med riskfaktorer för metabol sjukdom under tonårstiden då ”sjukdomskartan” under livet präglas.

Vi måste också värdera kirurgins effekter beträffande kroppslig utveckling, vitaminnivåer, kirurgiska bieffekter och andra potentiella negativa aspekter.

Projektbeskrivning

Vi planerar att:

Randomiserad studie där ungdomar mellan 13 och 16 års ålder

randomiseras till bästa medicinska omhändertagande med pulverkost behandling initialt eller gastric bypass-operation. Förutsättningen är att såväl ungdomen som vårdnadshavare kan tänka sig att acceptera kirurgisk behandling. Ungdomarna randomiseras till att antingen genomgå gastric bypass-operation direkt eller traditionell konservativ terapi, ungdomarna i denna grupp kommer att erbjudas kirurgi när de fyllt 18 år, om så önskas.

Inklusionskriterier:

• Ålder 13-15 år

• Iso-BMI ≥ 35 kg/m^2

• Genomfört minst ett års adekvat konventionell viktreducerande behandling.

• Pubertetsmognad Tanner >3

• Genomgått bedömning av psykolog

• Positiv inställning till långsiktig uppföljning.

Exklusionskriterier:

• Svårt bristande compliance

• Tidigare större bukkirurgi

• Specifikt fetmasyndrom som Prader Willis, Laurence Moon-Bardet-Biedl

• Fetma sekundärt till skador på CNS

• Pubertetsstadie Tanner ≤ 3 eller avsaknad av pubertet

• Psykotisk sjukdom

• Psykiskt handikappade

• Svår allmänsjukdom och därför olämpliga för allmän anestesi

• Leptinbrist eller homozygot MC4R defekt

Medicinsk behandling av övervikt

Det innebär en inledande Low Calorie Diet-period följt av fortsatt livsstilsbehandling med kost- och motionsråd och ev. läkemedelsbehandling. I den medicinska armen kommer studiedeltagarna att följas under studieperioden med intensivt multidiciplinärt omhändertagande med regelbundna besök på fetmamottagningarna vid Drottning Silvias Barn o Ungdomssjukhus samt vid Rikscentrum för barnobesitas, Karolinska sjukhuset, där bästa tillgängliga behandling mot fetma ska ges, diet och aktivitetsråd genom motiverande samtal (inklusive läkemedel).

Operationen

Operationen utförs av kirurger med stor vana av överviktsoperationer, särskilt med titthålskirurgi. Vid operationen kopplas det mesta av magsäcken förbi genom att tunntarmen dras upp och kopplas till en liten ficka som tillskapas av översta delen av magsäcken (s.k. Gastric Bypass). Ingen del av magsäcken eller tarm avlägsnas utan kopplas bara om på ett nytt sätt (se bild).

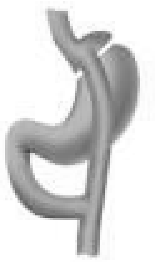

Operationen medför främst en minskad möjlighet till att äta stora portioner mat. Man ”styrs” också till att äta nyttigare genom att man efter ingreppet mår dåligt av att äta fet eller mycket söt mat. Portionsstorlekarna blir efter hand större och den opererade äter ganska ”normalt” ungefär två år efter operationen när viktnedgången avstannat. Oftast har då individen nått en vikt som motsvarar lätt övervikt. Operationerna görs med titthålsteknik vilket innebär att man opererar genom fem små hål i bukväggen. Operationerna kommer att utföras i samarbete mellan Carlanderska stiftelsen och avdelningarna för kirurgi och pediatrik vid Sahlgrenska universitetssjukhuset i Göteborg. Öppenvårdbesök (inklusionsbesök och postoperativa uppföljningar) kommer att ske såväl vid Drottning Silvias barn och ungdomssjukhus som vid barnkliniken på Astrid Lindgrens barnsjukhus.

Undersökningarna

En baslinjeundersökning kommer att genomföras innan behandlingsstart. Vidare studiebesök kommer att ske vid 1, 2, 5 och 10 år samt vid identifikation av adverse events. Studieundesökningar kommer också utföras när de opererade fyller 20, 25 respektive 30 år. Utöver dessa studiebesök kommer ungdomarna också vara på ytterligare kliniska besök där samordning tidsmässigt eftersträvas i störst möjliga utsträckning (vg se flödesschema, bilaga 1).

Undersökningar vid studiepunkterna kommer att utföras enligt nedan:

Besöket på mottagningen kommer att ta ca 4-5 timmar beroende på olika individers särskilda behov

- Frågeformulär
  - För livskvalitet: RAND-36, OP skalan (obesitasspecifikt QoL)
  - Psykisk hälsa: Beck Youth Inventory, 5-15, ASSQ, ARSR, Rosenberg Self Esteem, Mood Adjective Check List

  - Fysisk aktivitet/ Ätmönster: Diet history, TFEQ-R21, QEWP-R, IPAQ
  - Mag-tarmfunktion: Gastro-Intestinal symptom rating scale (GSRS), Dumping Symptom Rating Scale (DSRS)
  - Missbruk: AUDIT o DUDIT
- Blodprovsanalys
  - Totalt kolesterol, HDL-kolesterol, LDL-kolesterol och triglycerider och apo-lipoproteiner liksom plasma insulin o Glukos samt HbA1c kommer att analyseras Dessutom kommer vi att analysera en rad inflammatoriska markörer (supersensitivt CRP, IL-6, TNF-alfa och Adiponectin) samt analys av steroihormoner med massspektrometri.
    Genetiska prover för screening av kända fetmagener kommer att undersökas med snip-analyser och metyleringsstudier. Syftet med dessa undersökningar är dels att få en uppfattning om vissa genetiska profiler svarar sämre på kirurgisk terapi och dels för att kunna ge ungdomarna och deras vårdnadshavare information om det fanns en genetisk underliggande bakgrund till utvecklingen av svår fetma vilket i så fall kan vara till hjälp. Blodprovtagning kommer att insamlas med sammanlagt 50 ml extra blod vid samma tillfälle som rutinblodprovtagningen sker vid studiestart, 2, 12 och 24 samt 60 månader efter studiestart. Ackumulerad mängd extra under 5 år= 250 ml
- Endotelfunktion mätningar- Endotelfunktion kommer att bedömas med hjälp av reaktiv hyperemi perifer och arteriell tonometri (pulse vave velocity)
- Ultraljudsmätningar med högupplösande ultraljud på 55 MHz (Visualsonics)
- 24 timmars blodtrycksmätning. Sömnundersökningar i hemmiljö.
- Utveckling av överskottshud och fysisk aktivitetsnivå och prestationsförmåga undersöks.
- Hälsoekonomisk analys
  - Vi kommer att prospektiv insamla ackumulerade hälso- och sjukvårdskostnader i båda grupperna.
  - Vi planerar även att följa upp opererade ungdomar och kontroller avseende potentiella biverkningar, behov av läkemedel, samt hälsoekonomiska utfall genom länkning av kliniska data till nationella register såsom Patientregistret, Läkemedelsregistret, Cancerregistret och Dödsorsaksregistret vid Socialstyrelsen, samt Försäkringskassans register över sjukskrivning och sjuk-/aktivitetsersättning. Detta har tidigare gjorts inom den kontrollerade kliniska prövningen Swedish Obese Subjects (SOS-studien), vilken representanter från forskargruppen varit involverade i. Migrationsdata och socioekonomiska variabler (utbildning, civilstånd, inkomst) planeras att inhämtas från Statistiska centralbyrån.
- Kroppssammansättning - För kroppssammansättning kommer DEXA att användas (minimal stråldos ca 0,002mSv) undersökningen tar ca 15 min.
- Vi kommer att ta fram ett digitalt behandlingsstöd (hemsida, app) som är anpassat för ungdomsgruppen. Det digitala behandlingsstödet syftar till att fungera som ett sätt att öka följsamheten till det förändrade livsmönstret (främst vad gäller diet och motion), att säkerställa informationsbehovet hos gruppen samt en möjlighet till kommunikation mellan ungdomar och forskningsgruppen. Det digitala verktyget kommer att anpassas för ungdomsgruppens speciella behov avseende språkbruk, användarvänlighet och interaktivitet.
- Vi kommer också att prova ett kosttillskott, som är speciellt framtaget för att tillgodose det specifika behovet av daglig tillförsel av extra vitaminer och mineraler efter gastric bypass hos ungdomar. Tabletten ersätter andra tillskott (som visat sig ha dålig följsamhet hos tonåringar) i en och samma tablett som tas 1-3 gånger per dag. Tillskott av vitaminer och mineraler är viktigt hos gastric bypass-opererade för att undvika bristtillstånd.

Betydelse

Det mesta talar för att de positiva effekter som kan uppnås hos vuxna som genomgår kirurgi också kan erhållas vid operation av ungdomar under 18 år. Vi vill därför ge de ungdomar mellan 13 och 15 år med synnerligen

allvarlig fetma och medicinska komplikationer en möjlighet att genomgå operation, dock i fortsatt studieform. Vi anser oss ha ett ansvar att genomföra den randomiserade studie som i så fall kan visa att kirurgisk behandling är att rekommendera även för tonåringar.

Hälften av de som ingår i den randomiserade studien får möjligheten att genomgå operation och dess positiva konsekvenser medan den konservativt behandlade gruppen erbjuds en behandling som i regel är intensivare än gängse praxis för ungdomar.

Målet är att täcka den kunskapslucka som för närvarande föreligger inom sjukvården om individer under 18 års ålder med svår fetma kan återföras till ett normaliserat livslopp genom att genomföra en visserligen radikal men livsförändrande operation. Om så är fallet kommer en tidigare mycket utsatt grupp i samhället med mycket hög förväntad sjuklighet, låg livskvalitet och kraftigt reducerad förväntad livslängd kunna erbjudas en effektiv och livsförbättrande behandling. Även ur ett samhällsperspektiv kommer kostnader kunna reduceras genom minskad sjuklighet, sjukfrånvaro mm som uppstår till följd av fetman.

Referenser:

- Baker, J. L., et al. (2007). "Childhood body-mass index and the risk of coronary heart disease in adulthood." N Engl J Med 357(23): 2329-2337.
- Biro, F. M. and M. Wien (2010). "Childhood obesity and adult morbidities." Am J Clin Nutr 91(5): 1499S-1505S.
- Danielsson P, S. V., Kowalski J, Nyberg G, Ekblom Ö and Marcus C (2011). "Importance of age for three-year continuous behavioral obesity treatment success and dropout rate." Obesity facts.
- Fontaine, K. R., et al. (2003). "Years of life lost due to obesity." JAMA 289(2): 187-193.
- Jarvholm, K., et al. (2012) "Short-term psychological outcomes in severely obese adolescents after bariatric surgery." Obesity (Silver Spring) 2012(2): 318-323.
- O'Brien, P. E., et al. (2010). "Laparoscopic adjustable gastric banding in severely obese adolescents: a randomized trial." JAMA 303(6): 519-526.
- Olbers, T., et al. (2012). "Two-year outcome of laparoscopic Roux-en-Y gastric bypass in adolescents with severe obesity: results from a Swedish Nationwide Study (AMOS)." Int J Obes (Lond) 36(11): 1388-1395.
- Sjostrom, L., et al. (2012). "Bariatric surgery and long-term cardiovascular events." JAMA 307(1): 56-65.

Informed consent for participants aged ≥ 15 years.

Studieinformation till ungdomar som fyllt 15 år

Behandling av svår fetma hos ungdomar-

Jämförande studie mellan medicinsk behandling och magsäcksoperation

BAKGRUND TILL STUDIEN

Fetma är ett svårbehandlat tillstånd. Hos vuxna är magsäcksoperation en ganska vanlig behandling som leder till viktnedgång och förbättrad livskvalitet och hälsa. Det är fortfarande oklart om magsäcksoperationer ska användas hos ungdomar under 18 år. Vi har skaffat oss en del erfarenheter av operationer hos tonåringar men vi behöver genomföra fortsatta studier innan vi har tillräckligt underlag för att veta vad vi ska rekommendera.

Förfrågan om deltagande

Vi vill tillfråga Dig om deltagande i denna studie eftersom Du behandlats för viktproblem på den mottagning Du går och att Du är i den åldern som vi vill studera.

HUR GÅR STUDIEN TILL?

Innan du bestämmer dig för att delta i studien eller inte kommer du att få en hel del information och du ska ha möjlighet att ställa alla frågor du har.

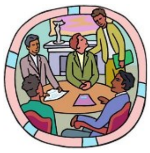

Du får träffa barnläkare, sjuksköterska, dietist, psykolog och kirurg som alla utifrån sitt perspektiv kan berätta vad studien innebär.

Flera av dessa samtal kan samordnas, men ofta är det bra att få information i flera omgångar för att hinna tänka emellan.

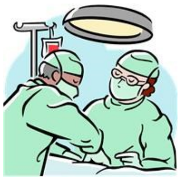

JA/NEJ

Om du efter denna information vill vara med i studien så kommer Du att föreslås ett av nedanstående behandlingsalternativ. Du kan inte själv välja vilket, utan just Din behandling avgörs av ett på förhand uppgjort schema, ett val som varken Du som patient eller vi som behandlare kan påverka.

?

- **Operation med titthålskirurgisk gastric bypass**

  alternativt till

- **Intensiv medicinsk behandling -**Du får komma till oss för behandling med kost och motion 1 besök/månad och eventuellt läkemedel. Behandlingen inleds med att Du får äta pulverkost under åtta veckor i början av behandlingen som gör att du snabbt går ner i vikt.

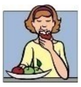 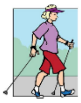

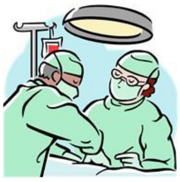

Operation

Du som föreslås kirurgi får ytterligare information. Operationen görs med titthålsteknik vilket innebär att man opererar genom fem små hål i bukväggen. Vid operationen kopplar man förbi det mesta av magsäcken genom att tunntarmen kopplas till en liten ficka på översta delen av magsäcken (s.k. gastric pypass). Ingen del av magsäcken eller tarm avlägsnas utan kopplas bara om på ett nytt sätt (se bild).

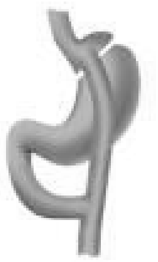

Operationen innebär en minskad möjlighet att äta stora portioner mat. Man ”styrs” också till att äta nyttigare genom att man efter ingreppet mår dåligt av att äta fet eller mycket söt mat. Portionsstorlekarna blir efter hand större och den opererade äter ganska normalt ett-två år efter operationen när viktnedgången avstannat. Oftast har man då en betydligt mindre övervikt.

Efter operationen kommer du följas upp av ett team bestående av läkare, dietister, psykologer och sjuksköterskor. Första återbesöket blir efter 8 veckor

Konservativ behandling med pulverkost

Du som föreslås traditionell behandling kommer att få intensiv medicinsk behandling med en inledande lågkaloribehandling (pulverkost) som därefter följs upp med samtal och råd om kost-, motion på mottagningen ca en gång per månad. Hos vissa kan läkemedelsbehandling bli aktuellt. Sammanlagt är det 10 ”vanliga” besök på mottagningen och ett eller två extra studiebesök därefter. Du kommer att följas under två år. Varje behandlings besök tar ungefär en timme och studiebesöken en gång per år tar ca 4-5 timmar

Om du senare vill bli opererad kommer Du att erbjudas operation med gastric bypass tidigast när Du fyllt 18 år. Om du inte väljer att vara med i studien kan Du inte välja operation eftersom magsäcksoperationer bara görs i denna studie i Din åldersgrupp.

Vad vill vi undersöka i studien?

Oavsett vilken grupp Du hamnar i så kommer Du att bli noggrant och väl omhändertagen! Du får genomgå en hel del kontroller före studien och efter 1, 2, 5 och 10 år. Vi vill följa dig i minst 10 år för att kunna utvärdera nyttan av de olika behandlingarna. Du kommer också däremellan kallas till andra besök hos din ordinarie läkare.

Vid särskilda kontrollerna i studien så får Du göra detta:

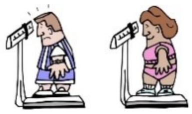

- Vi mäter Din vikt, längd och midje- och höftmått

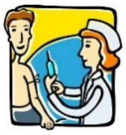

- Du får lämna blod och urinprov. Du få EMLA-plåster innan om du vill. Salvan som finns i plåstret gör att huden blir bedövad så att sticket inte skall kännas så mycket.

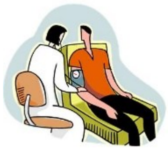

- Blodtryck kontrolleras med att du får ha på dig en blodtryckmätare som mäter ditt blodtryck under 24 timmar. Ett ”band” pumpas upp och kramar till din överarm med jämna mellanrum. Det är viktigt att du inte har på mätaren om du duschar eller badar.

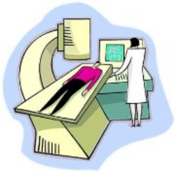

- Vi gör en undersökning med en apparat där vi kan mäta kroppssammansättningen (fördelningen av muskler, fett o skelett). Du får ligga stilla på en brits och apparaten som tar bilderna rör sig över dig. Undersökningen tar ca 15 minuter. Den så kallade DXA-undersökningen ger en mycket låga stråldos. Så om Du vill kan Din förälder vistas i rummet vid undersökning

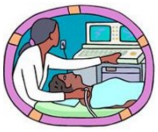

- Ultraljud och undersökning av dina blodkärl
  Dina blodkärl undersöks med ultraljud på arm, fot och hals. Du får ligga på en brits och vila och det ger inget ger obehag, bara en dosa som trycks mot huden.

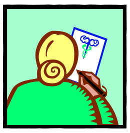

- Du kommer att få fylla i frågeformulär om bland annat hur Du mår, hur Du brukar äta och hur Du mår då man ätit.

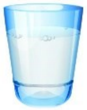

- Sockertest
  Först får Du ett stick i fingret och sen du får dricka en väldigt söt dryck. Efter 2 timmar tar vi ett stick igen. I blodet som vi samlar efter sticket i fingret mäter vi om blodsockret ligger bra och om ditt insulin fungerar som det ska.

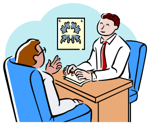

- Samtal med läkare, sjuksköterska, sjukgymnast, psykolog och dietist.

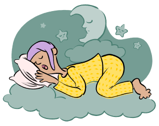

- Du får med dig en liten apparat hem som du kopplar på när du lägger dig och kopplar av på morgonen. Apparaten mäter hur du andas när du sover

Total tidsåtgång för hela besöket: Cirka 4-5 timmar

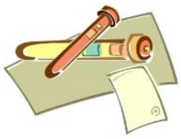

Biobanksprover

De blodprover som tas i studien och inte analyseras direkt sparas i en så kallad ”biobank” som är godkänd av Socialstyrelsen (enl. lagen 2002;297). Blodproverna kodas på samma sätt som övrig data inom studien och enbart studieansvarig har tillgång till kodnyckeln. Du kommer att få godkänna att vi tar dessa prover och inga undersökningar kan göras med dessa prover utan att Du först informeras och godkänt det.

Patientregister

Vi kommer att registrera Dina data i två kvalitetsregister i svensk sjukvård, dels i Barnobesitasregistret i Sverige (BORIS) och vid operation även i Skandinaviskt Obesitaskirurgiskt Register (SOREG). Uppgifter som Din vikt, blodprov, blodtryck, typ av behandling, andra speciella händelser som kan ha med behandlingen att göra registreras. Dessa registerdata samlas in rutinmässigt så Du utsätts inte för några ytterligare risker eller tidskrävande undersökningar

Vi vill även följa eventuella biverkningar, behov av läkemedel, genom en länkning till andra nationella register såsom Patientregistret, Läkemedelsregistret, Cancerregistret och Dödsorsaksregistret vid Socialstyrelsen, samt Försäkringskassans register över sjukskrivning och sjuk-/aktivitetsersättning. Migrationsdata och socioekonomiska variabler (utbildning, civilstånd, inkomst) kommer att inhämtas från Statistiska centralbyrån.”

När man samkör dessa registerdata görs det alltid på anonymiserade data. Resultat kommer enbart tas ut på gruppnivå så att enskilda individer inte kan spåras.

Vilka är riskerna?

Ca 1 av 20 patienter som opereras får en komplikation (t.ex. blödning, infektion, läckage och stopp i tarmpassagen). Ungefär 1 av 50 får en allvarlig komplikation som kan leda till att blodtransfusion eller omoperation behövs. Risken att dö av komplikation är mycket låg (1 av 2000 hos vuxna).

Om man som gastric bypass-opererad äter för fort, för mycket eller fel typ av mat kan man uppleva obehagliga men ofarliga symptom som vi kallar "dumpning" (t.ex. snabb puls, svettningar, trötthet).

Den som är magsäcksopererad behöver under resten av livet ta tillskott av vitaminer och andra ämnen.

Det kan vara svårt att hantera uppfattningen av sin ”nya” kropp efter viktnedgång och många upplever besvär med överflödig hud som inte dragit ihop sig tillräckligt.

Man blir mycket känslig för alkohol efter gastric bypass. Berusning kommer lättare och det finns en liten, men ändå ökad risk att hamna i missbruk av alkohol.

Blodprovstagning kan upplevas som obehaglig. Erfarna sjuksköterskor och hjälp med bedövningssalva (om så önskas) minskar dock detta problem i allmänhet. Övriga undersökningar uppfattas sällan som besvärliga.

Finns det några fördelar?

Du får i studien möjlighet att genomgå behandlingar med nya metoder som inte används normalt i Din åldersgrupp. Förhoppningen är att dessa leder till god viktnedgång. Resultatet kan se lite olika ut för olika individer men målsättningen är att Du ska få lättare att röra på dig och förhoppningsvis sova bättre.

Har du högt blod socker så brukar det förbättras efter viktnedgång liksom blodtryck och blodfetter. Detta är kanske inte något som Du märkt så mycket av tidigare men det är något som kan påverka Din hälsa längre fram.

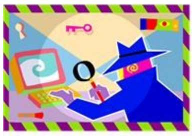

Hantering av data och sekretess

Alla insamlade uppgifter om Dig hanteras kodat, d v s ditt namn och personnummer finns inte med i listorna utan istället ett nummer. Svar och resultat kommer att behandlas och skyddas enligt personuppgiftslagen. Ingen obehörig kommer att få tillgång till Dina data

Ansvarig för behandlingen av personuppgifter är utförarstyrelsen för Sahlgrenska Universitetssjukhuset. Du kan vända dig till sjukhusets personuppgiftsombud, tel 031-3432726, Enheten för informationssäkerhet, Torggatan 1A, 431 35 Mölndal.

I ombudets uppgifter ingår bl. a att kunna ge en allmän information om de rättigheter och skyldigheter Du har.

Hur får jag information om studiens resultat?

Du kommer under studiens gång få besked om Dina provsvar och om Du önskar kommer att få en sammanfattning av undersökningsresultaten. Efter studien kommer Du att inbjudas till en information om vad vi har kommit fram till med studien.

Försäkring etc.

Du är skyddad av patientdatalagen och Sjukvårdens patientförsäkring gäller

Det utgår ingen ekonomisk ersättning för deltagande i studien.

Frivillighet

Deltagandet i studien är frivilligt och om Du inte vill vara med kan Du avbryta när som helst under studiens gång utan att behöva förklara varför. Du kommer då att bli erbjuden fortsatt traditionell behandling

Ansvariga

Har du frågor, svarar vi gärna på dem

Studieledare:

Doktor Torsten Olbers,

Telefon: 031 - 3428347 E-post: torsten.olbers@gu.se

Astrid Lindgrens Barnsjukhus

Doktor Annika Janson,

Telefon: 0707-375147 E-post: annika.janson@karolinska.se

Koordinator Kerstin Ekbom,

Telefon: 08-58587334 E-post: kerstin.ekbom@karolinska.se

Drottning Silvias Barn och Ungdomssjukhus

Doktor Jovanna Dahlgren,

Telefon: 0702-750233 E-post: jovanna.dahlgren@vgregion.se

Koordinator Eva Gronowitz,

Telefon: 031-3434690 E-post: eva.gronowitz@vgregion.se

Informed consent for caregivers

Studieinformation till föräldrar/vårdnadshavare

Behandling av svår fetma hos ungdomar-

Jämförande studie mellan medicinsk behandling och magsäcksoperation

Bakgrund till studien

Det är svårt att hitta en bra och effektiv behandling för ungdomar med kraftig övervikt – fetma. Kost- och motionsråd, beteendeterapi, familjeterapi, behandling med läkemedel eller specialkoster ger ofta inte bestående goda resultat vid behandling i tonåren. Vi vet att man efter magsäckskirurgi på vuxna med obesitas kan få en god och bestående viktnedgång kombinerat med ett bra sätt att äta. Viktnedgången har också positiva effekter på livskvalitén och hälsa. Operationer mot fetma utförs idag enbart på vuxna, d.v.s. 18 år är den nedre åldersgränsen. Det är fortfarande oklart om magsäcksoperationer ska användas hos ungdomar under 18 år. Vi har skaffat oss en del erfarenheter av operationer hos tonåringar men vi behöver genomföra fortsatta studier innan vi har tillräckligt underlag för att veta vad vi ska rekommendera. Vi vill i denna studie undersöka om denna sortens operation är bra som behandling även för ungdomar med fetma. Genom att jämföra ungdomar med fetma som opereras och som får behandling mot sin övervikt på det traditionella sättet kan vi se om det blir någon skillnad i vikten och hur man mår på längre sikt.

Förfrågan om deltagande

Studien genomförs på ungdomar som kommit i kontakt med överviktsenheterna inom specialistsjukvården. De skall ha påbörjat sin pubertet och vara 13 till 15 år gamla. Ungdomar med fetma som inte lyckats få bestående viktnedgång på minst ett års vanlig behandling kommer att tillfrågas om medverkan i studien.

Vi vill tillfråga Ditt Barn om deltagande i denna studie eftersom vi funnit att Ditt Barn kan vara aktuell utifrån de viktuppgifter vi har på Ditt Barn och att Ditt Barn är i den åldern som vi vill studera.

HUR GÅR STUDIEN TILL?

Innan Ni bestämmer er för att delta i studien eller inte kommer Ni att få en hel del information och Ni ska ha möjlighet att ställa alla frågor Ni har. Ni får träffa barnläkare, sjuksköterska, dietist, psykolog och kirurg som alla utifrån sitt perspektiv kan berätta vad studien innebär.

Flera av dessa samtal kan samordnas, men ofta är det bra att få information i flera omgångar för att hinna tänka emellan. Om Ni efter denna information säger **JA** till att ert Barn kan genomgå en magsäcksoperation så är det viktigt att Ni är medvetna om att Ert barn enligt ett datorprogram slumpvis föreslås:

- magsäcksoperation

  eller

- intensifierad medicinsk behandling, dvs. motiverande samtal med kost och motionsråd. Behandlingen kommer att inledas med en 8 veckors pulverkost behandling

Om Ditt Barn föreslagits medicinsk behandling och vill bli opererad så kan Ditt Barn erbjudas operation med gastric bypass tidigast när han/hon fyllt 18 år eller i undantagsfall tidigare om allvarliga fetmakomplikationer uppstår. Om Ditt Barn inte väljer att vara med i studien kan Ditt Barn inte välja operation eftersom magsäcksoperationer bara görs inom ramen för denna studie i Ditt Barns åldersgrupp.

Operationen

De som får förslag om kirurgi får ytterligare information via lokal studieledare innan man de sätts upp för kirurgi. Operationer utförs av kirurger med stor vana av överviktsoperationer med titthålskirurgi. Vid operationen kopplas det mesta av magsäcken förbi genom att tunntarmen dras upp och kopplas till en liten ficka som tillskapas av översta delen av magsäcken (s.k. Gastric Bypass). Ingen del av magsäcken eller tarm avlägsnas utan kopplas bara om på ett nytt sätt (se bild).

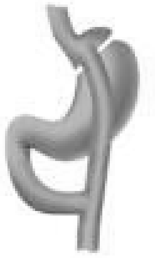

Operationen medför främst en minskad möjlighet till att äta stora portioner mat. Man ”styrs” också till att äta nyttigare genom att man efter ingreppet mår dåligt av att äta fet eller mycket söt mat. Portionsstorlekarna blir efter hand större och den opererade äter ganska ”normalt” ungefär två år efter operationen när viktnedgången avstannat. Oftast har då individen nått en vikt som motsvarar måttlig övervikt. Operationerna görs med titthålsteknik vilket innebär att man opererar genom fem små hål i bukväggen.

Konservativ behandling

De som föreslås medicinsk behandling kommer att få fortsatt intensifierad livsstilsbehandling med kost, motionsråd och motiverande samtal på mottagningen ca en gång per månad efter ett uppgjort schema. Ditt barn kommer också att få påbörja behandlingen med pulverkost diet under 8 veckor i början av behandlingen som oftast leder till en påtaglig snabb viktnedgång.

Uppföljning i studien

Oavsett vilken grupp Ditt Barn hamnar i så kommer vi att följa Ditt Barn med täta kontroller. I början av studien lite tätare och senare glesnar det ut. Vi vill följa Ditt Barn i 10 år för att kunna utvärdera nyttan av de olika behandlingarna. Båda grupperna skall genomföra undersökningar och uppföljningar på samma sätt, studien går ut på att jämföra långtidsresultaten i de två grupperna. Enda skillnaden mellan grupperna är själva operationen. Studiekontrollerna sker vid intagningen i studien och efter 8 veckor därefter vid 1,2,5 och 10 år. Under tiden studien pågår kommer man få träffa läkare, sjuksköterska, dietist och psykolog vid bestämda tidpunkter. Vi vill mäta och utvärdera:

- Viktminskningen och hur den påverkar kroppssammansättningen.
- Förekomst av ev. följdsjukdomar som ofta uppkommer i samband med svår övervikt, (t ex högt blodtryck, förhöjda blodfettsnivåer, diabetes mm).
- Hur den fysiska aktiviteten påverkats
- Hur kost och näringsintaget fungerar efter behandlingen
- I vilken mån följderna av behandlingen har påverkat synen på tillvaron och det psykiska måendet (livskvalitén).

Vad vill vi undersöka i studien vi de olika studiebesöken?

Undersökningarna som ingår är följande:

- Blodprov (kommer att tas när Ditt Barn är fastande på morgonen av van sjuksköterska), mängden blod som tas (ca 50 ml) kommer inte att påverka ditt Barns allmäntillstånd.
- Mätning av vikt, läng midje-, höft-, arm-, hals- omfång
- Mätning av blodtryck
- DXA-undersökning visar på Ditt Barns kroppsammansättning (muskel- fett- och skelettvävnad) och bentäthet. DXA tekniken ger mycket låga stråldoser så vill Du som förälder kan Du vistas i rummet vid undersökningen (tar ca 15 min).
- Undersökning av blodkärl med ultraljud
- Frågeformulär om hur man mår – i sitt humör, hur man brukar äta och hur man mår då man ätit.
- Sockertest, Ditt Barn får dricka socker och så tar vi ett stick i fingret före och efter 2 timmar. Detta för att undersöka att blodsockret ligger bra och att Ditt Barns insulin fungerar som det ska.
- En dietist intervjuar Ditt Barn om matval och matvanor
- studiebesök tidsåtgång ca 4-5 timmar

Biobanksprover

De blodprover som tas i studien och inte analyseras direkt sparas i biobank, godkänd av Socialstyrelsen (enl. lagen 2002;297). Skriftligt samtycke inhämtas från Ditt Barn och från Er som föräldrar innan provtagning. Blodproverna kodas på samma sätt som övrig data inom studien och enbart studieansvarig har tillgång till kodnyckeln.

Vilka är riskerna?

Tyvärr är ingen operation helt riskfri. Ca 1 av 20 patienter som opereras får någon slags komplikation (t.ex. blödning, infektion, läckage och stopp i tarmpassagen). Ungefär 1 av 50 får en allvarlig komplikation som kan leda till att blodtransfusion eller omoperation behövs. Det finns alltid en minimal risk att dö vid en operation men stora studier kring detta visar att risken bland vuxna vid denna sortens operation är mycket låg (1 av 2000 hos vuxna).

Om man som gastric bypass-opererad äter för fort, för mycket eller fel typ av mat kan man uppleva obehagliga men ofarliga symptom som vi kallar "dumpning" (t.ex. snabb puls, svettningar, trötthet).

Den som är magsäcksopererad behöver under resten av livet ta tillskott av vissa vitaminer och andra ämnen.

Det kan vara svårt att hantera uppfattningen av sin ”nya” kropp efter viktnedgång och många upplever besvär med överflödig hud som inte dragit ihop sig tillräckligt.

Man blir mycket känslig för alkohol efter gastric bypass. Berusning kommer lättare och det finns en liten, men ändå ökad risk att hamna i missbruk av alkohol.

Blodprovstagning kan upplevas som obehaglig. Erfarna sjuksköterskor och hjälp med bedövningssalva (om så önskas) minskar dock detta problem i allmänhet. Övriga undersökningar uppfattas sällan som besvärliga.

Finns det några fördelar?

Detta är en studie som går ut på att följa behandlingarnas medicinska hälsoeffekter, fysiskt och psykiskt. De som deltar i studien har under denna tid regelbunden kontakt med extra kunnig personal och en noggrann uppföljning av hälsotillståndet kommer att göras i båda grupperna. Ni har bästa möjliga tillgång till medicinsk utredning samt till behandling.

Ditt barn får möjlighet att genomgå behandlingar med nya metoder som inte används normalt i denna åldersgrupp. Förhoppningen är att dessa leder till god viktnedgång. Resultatet kan se lite olika ut för olika individer men målsättningen är att Ditt Barn skall få lättare att röra på dig och må bättre.

Har Ditt Barn högt blodsocker så brukar det förbättras efter viktnedgång liksom blodtryck och blodfetter. Detta är kanske inte något som Du eller Ditt Barn märkt så mycket av tidigare men det är något som kan påverka Ditt Barns hälsa längre fram.

Hantering av data och sekretess

Alla insamlade uppgifter kommer att datorbearbetas med ditt Barns identitet kodad, dvs namn och personnummer finns inte med i listorna utan istället finns ett nummer. Svar och resultat kommer att behandlas och skyddas enligt personuppgiftslagen (PUL).

Ansvarig för behandlingen av personuppgifter är utförarstyrelsen för Sahlgrenska Universitetssjukhuset. Du/Ni kan vända er till sjukhusets personuppgiftsombud, tel 031-3432726, Enheten för informationssäkerhet, Torggatan 1A, 431 35 Mölndal om Ni har några frågor.I ombudets uppgifter ingår bl.a att kunna ge en allmän information om de rättigheter och skyldigheter vi har att iaktta när vi arbetar med datoriserade uppgifter. Ombudet skall även hjälpa de patienter, anställda och anhöriga som har frågor om datoriserade rutiner.

Hur får jag information om studiens resultat?

Ni kommer under studiens gång få besked om Ert Barns provsvar. Efter studien kommer Du och Ditt Barn att inbjudas till en information om vad vi har kommit fram till med studien. Resultaten kommer också att publiceras i vetenskapliga tidskrifter.

Försäkring

Sjukvårdens patientförsäkring gäller.

Frivillighet

Deltagandet i studien är frivilligt och kan avbrytas när som helst under studiens gång utan att ange orsak eller att det påverkar Ditt Barns behandling. Den sedvanliga kontakten med läkare och annan personal på mottagningen fortsätter som tidigare.

Eftersom operation under 18 års ålder idag inte är en rutinbehandling kan vi inte erbjuda kirurgi om man inte deltar i studien.

Ansvariga

Har du frågor, svarar vi gärna på dem

Studieledare:

Doktor Torsten Olbers,

Telefon: 031 - 3428347 E-post: torsten.olbers@gu.se

Astrid Lindgrens Barnsjukhus

Doktor Annika Janson,

Telefon: 0707-375147 E-post: annika.janson@karolinska.se

Koordinator Kerstin Ekbom,

Telefon: 08-58587334 E-post: kerstin.ekbom@karolinska.se

Drottning Silvias Barn och Ungdomssjukhus

Doktor Jovanna Dahlgren,

Telefon: 0702-750233 E-post: jovanna.dahlgren@vgregion.se

Koordinator Eva Gronowitz,

Telefon: 031-3434690 E-post: eva.gronowitz@vgregion.se

Behandling av svår fetma hos ungdomar- Jämförande studie mellan medicinsk behandling och magsäcksoperation

Samtyckesformulär ***Svarsblankett för föräldrar/Vårdnadshavare***

Ansvarig läkare: Dr Torsten Olbers

Mitt barns namn: ____

Mitt barns personnummer: _______________________________________

☐ Ja, Jag/Vi vill att mitt/vårt barn deltar i studien. Jag/Vi har tagit del av informationen om studien och godkänner det upplägg beskrivs i informationen. Vi har också fått tillfälle att ställa frågor, fått dem besvarade.

Datum: _______________ Ort: ___________________________________

Förälders/ Vårdnadshavares underskrift:

____

Namnförtydligande (TEXTAT) _______________________________________

Datum: _______________ Ort: ___________________________________

Förälders/ Vårdnadshavares underskrift:

____

Namnförtydligande (TEXTAT) _______________________________________

Behandling av svår fetma hos ungdomar- Jämförande studie mellan medicinsk behandling och magsäcksoperation

Ansvarig läkare: Dr Torsten Olbers

Namn: ____

Personnummer: ____

☐ Ja, Jag vill delta i studien. Jag har tagit del av informationen om studien och godkänner det upplägg beskrivs i informationen. Jag har också fått tillfälle att ställa frågor och fått dem besvarade.

Datum: _________________ Ort: ______________________

Namnunderskrift: ______________________________

Adress: ______________________________

____

Telefon: ______________________________ Epost: ______________________________

Jag bekräftar att Jag har informerat om studien och gett tillfälle att ställa frågor

Namnteckning ______________________________ Datum_________________

Namnförtydligande ______________________________

Ansvarig läk/ssk

Assent for participants aged < 15 years
Studieinformation till ungdomar mellan 13-14 år

Behandling av svår fetma hos ungdomar-

Jämförande studie mellan medicinsk behandling och magsäcksoperation

BAKGRUND TILL STUDIEN

Fetma är ett svårbehandlat tillstånd. Hos vuxna är magsäcksoperation en ganska vanlig behandling som leder till viktnedgång och förbättrad livskvalitet och hälsa. Det är fortfarande oklart om magsäcksoperationer ska användas hos ungdomar under 18 år. Vi har skaffat oss en del erfarenheter av operationer hos tonåringar men vi behöver genomföra fortsatta studier innan vi har tillräckligt underlag för att veta vad vi ska rekommendera.

Förfrågan om deltagande

Vi vill tillfråga Dig om deltagande i denna studie eftersom Du behandlats för viktproblem på den mottagning Du går och att Du är i den åldern som vi vill studera.

HUR GÅR STUDIEN TILL?

Innan du bestämmer dig för att delta i studien eller inte kommer du att få en hel del information och du ska ha möjlighet att ställa alla frågor du har.

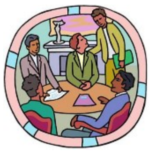

Du får träffa barnläkare, sjuksköterska, dietist, psykolog och kirurg som alla utifrån sitt perspektiv kan berätta vad studien innebär.

Flera av dessa samtal kan samordnas, men ofta är det bra att få information i flera omgångar för att hinna tänka emellan.

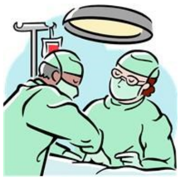

JA/NEJ

Om du efter denna information vill vara med i studien så kommer Du att föreslås ett av nedanstående behandlingsalternativ. Du kan inte själv välja vilket, utan just Din behandling avgörs av ett på förhand uppgjort schema, ett val som varken Du som patient eller vi som behandlare kan påverka.

?

- **Operation med titthålskirurgisk gastric bypass**

alternativt till

- **Intensiv medicinsk behandling**. Du får komma till oss för behandling med kost och motion 1 besök/månad och eventuellt läkemedel. Behandlingen inleds med att Du får äta pulverkost under åtta veckor i början av behandlingen som gör att du snabbt går ner i vikt

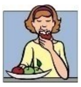
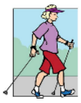

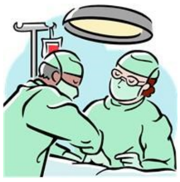

Operation

Du som föreslås kirurgi får ytterligare information. Operationen görs med titthålsteknik vilket innebär att man opererar genom fem små hål i bukväggen. Vid operationen kopplar man förbi det mesta av magsäcken genom att tunntarmen kopplas till en liten ficka på översta delen av magsäcken (s.k. gastric pypass). Ingen del av magsäcken eller tarm avlägsnas utan kopplas bara om på ett nytt sätt (se bild).

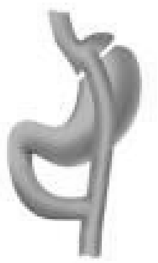

Operationen innebär en minskad möjlighet att äta stora portioner mat. Man ”styrs” också till att äta nyttigare genom att man efter ingreppet mår dåligt av att äta fet eller mycket söt mat. Portionsstorlekarna blir efter hand större och den opererade äter ganska normalt ett-två år efter operationen när viktnedgången avstannat. Oftast har man då en betydligt mindre övervikt.

Efter operationen kommer du följas upp av ett team bestående av läkare, dietister, psykologer och sjuksköterskor. Första återbesöket blir efter 8 veckor

Konservativ behandling med pulverkost

Du som föreslås traditionell behandling kommer få intensiv medicinsk behandling med en inledande lågkaloribehandling (pulverkost), som därefter följs upp med samtal och råd om kost och motion på mottagningen ca en gång per månad. Hos vissa kan läkemedelsbehandling bli aktuellt. Sammanlagt är det 10 ”vanliga” besök på mottagningen och ett eller två extra studiebesök därefter. Du kommer att följas under två år. Varje behandlingsbesök tar ungefär en timme och studiebesöken en gång per år tar 4-5 timmar

Om du senare vill bli opererad kommer Du att erbjudas operation med gastric bypass tidigast när Du fyllt 18 år. Om du inte väljer att vara med i studien kan Du inte välja operation eftersom magsäcksoperationer bara görs i denna studie i Din åldersgrupp.

Vad vill vi undersöka i studien?

Oavsett vilken grupp Du hamnar i så kommer Du att bli noggrant och väl omhändertagen! Du får genomgå en hel del kontroller före studien och efter 1, 2, 5 och 10 år. Vi vill följa dig i minst 10 år för att kunna utvärdera nyttan av de olika behandlingarna. Du kommer också där emellan kallas till andra besök hos din ordinarie läkare.

Vid de särskilda kontrollerna i studien så får Du göra detta:

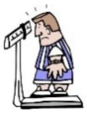
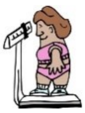

- Vi mäter Din vikt, längd och midje- och höftmått

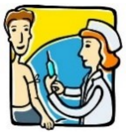

- Du får lämna blod och urinprov. Du få EMLA-plåster innan om du vill. Salvan som finns i plåstret gör att huden blir bedövad så att sticket inte skall kännas så mycket.

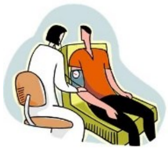

- Blodtryck kontrolleras med att du får ha på dig en blodtrycksmätare som mäter ditt blodtryck under 24 timmar. Ett ”band” pumpas upp och kramar till din överarm med jämna mellanrum. Det är viktigt att du inte har på dig mätaren om du duschar eller badar.

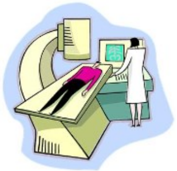

- Vi gör en undersökning med en apparat där vi kan mäta kroppssammansättningen (fördelningen av muskler, fett o skelett). Du får ligga stilla på en brits och apparaten som tar bilderna rör sig över dig. Undersökningen tar ca 15 minuter. Den så kallade DXA undersökningen ger en mycket låga stråldos. Så om Du vill, kan Din förälder vistas i rummet vid undersökning

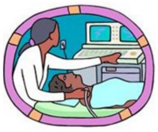

- Ultraljud och undersökning av dina blodkärl
  Dina blodkärl undersöks med ultraljud på arm, fot och hals. Du får ligga på en brits och vila och det ger inget ger obehag, bara en dosa som trycks mot huden.

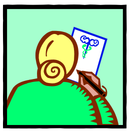

- Du kommer att få fylla i frågeformulär om bland annat hur Du mår, hur Du brukar äta och hur Du mår då man ätit.

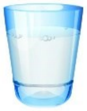

- Sockertest
  Först får Du ett stick i fingret och sen Du får dricka en väldigt söt dryck. Efter 2 timmar tar vi ett stick igen. I blodet som vi samlar efter sticket i fingret mäter vi om blodsockret ligger bra och om ditt insulin fungerar som det ska.

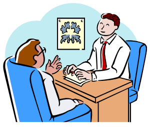

- Samtal med läkare, sjuksköterska, sjukgymnast, psykolog och dietist.

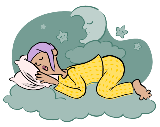

- Sömnregistrering
  Du får med dig en liten apparat hem som du kopplar på när du lägger dig och kopplar av på kvällen. Apparaten mäter hur du andas när du sover

Total tidsåtgång för hela besöket: Cirka 4-5 timmar

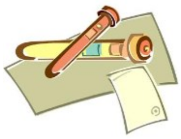

Biobanksprover

De blodprover som tas i studien och inte analyseras direkt sparas i en så kallad ”biobank”, som är godkänd av Socialstyrelsen (enl. lagen 2002;297). Blodproverna kodas på samma sätt som övrig data inom studien och enbart studieansvarig har tillgång till kodnyckeln. Du kommer att få godkänna att vi tar dessa prover och inga undersökningar kan göras med dessa prover utan att Du först informerats och godkänt det.

Patientregistrering

Vi kommer att registrera Dina data i två kvalitetsregister i svensk sjukvård, dels i Barnobesitasregistret i Sverige (BORIS) och vid operation även i Skandinaviskt Obesitaskirurgiskt Register (SOREG).

Uppgifter som Din vikt, blodprov, blodtryck, typ av behandling, andra speciella händelser som kan ha med behandlingen att göra registreras. Dessa registerdata samlas in rutinmässigt, så Du utsätts inte för några ytterligare risker eller tidskrävande undersökningar.

Vi vill även följa eventuella biverkningar, behov av läkemedel, genom en länkning till andra nationella register såsom Patientregistret, Läkemedelsregistret, Cancerregistret och Dödsorsaksregistret vid Socialstyrelsen, samt Försäkringskassans register över sjukskrivning och sjuk-/aktivitetsersättning. Migrationsdata och socioekonomiska variabler (utbildning, civilstånd, inkomst) kommer att inhämtas från Statistiska centralbyrån.”

När man samkör dessa registerdata görs det alltid på anonymiserade data. Resultat kommer enbart tas ut på gruppnivå så att enskilda individer inte kan spåras.

Vilka är riskerna?

Ca 1 av 20 patienter som opereras får en komplikation (t.ex. blödning, infektion, läckage och stopp i tarmpassagen). Ungefär 1 av 50 får en allvarlig komplikation som kan leda till att blodtransfusion eller omoperation behövs. Risken att dö av komplikation är mycket låg (1 av 2000 hos vuxna).

Om man som gastric bypass-opererad äter för fort, för mycket eller fel typ av mat kan man uppleva obehagliga men ofarliga symptom som vi kallar "dumpning" (t.ex. snabb puls, svettningar, trötthet).

Den som är magsäcksopererad behöver under resten av livet ta tillskott av vitaminer och andra ämnen.

Det kan vara svårt att hantera uppfattningen av sin ”nya” kropp efter viktnedgång och många upplever besvär med överflödig hud som inte dragit ihop sig tillräckligt.

Man blir mycket känslig för alkohol efter gastric bypass. Berusning kommer lättare och det finns en liten, men ändå ökad risk att hamna i missbruk av alkohol.

Blodprovstagning kan upplevas som obehaglig. Erfarna sjuksköterskor och hjälp med bedövningssalva (om så önskas) minskar dock detta problem i allmänhet. Övriga undersökningar uppfattas sällan som besvärliga.

Finns det några fördelar?

Du får i studien möjlighet att genomgå behandlingar med nya metoder som inte används normalt i Din åldersgrupp. Förhoppningen är att dessa leder till god viktnedgång. Resultatet kan se lite olika ut för olika individer men målsättningen är att Du ska få lättare att röra på dig och förhoppningsvis sova bättre.

Har du högt blodsocker så brukar det förbättras efter viktnedgång liksom blodtryck och blodfetter. Detta är kanske inte något som Du märkt så mycket av tidigare men det är något som kan påverka Din hälsa längre fram.

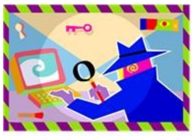

Hantering av data och sekretess

Alla insamlade uppgifter om Dig hanteras kodat, d v s ditt namn och personnummer finns inte med i listorna utan istället ett nummer. Svar och resultat kommer att behandlas och skyddas enligt personuppgiftslagen. Ingen obehörig kommer att få tillgång till Dina data

Ansvarig för behandlingen av personuppgifter är utförarstyrelsen för Sahlgrenska Universitetssjukhuset. Du kan vända dig till sjukhusets personuppgiftsombud, tel.

031-3432726, Enheten för informationssäkerhet, Torggatan 1A, 431 35 Mölndal.

I ombudets uppgifter ingår bl. a att kunna ge en allmän information om de rättigheter och skyldigheter Du har.

Hur får jag information om studiens resultat?

Du kommer under studiens gång få besked om Dina provsvar och om Du önskar kommer att få en sammanfattning av undersökningsresultaten. Efter studien kommer Du att inbjudas till en information om vad vi har kommit fram till med studien.

Försäkring etc.

Du är skyddad av patientdatalagen och Sjukvårdens patientförsäkring gäller

Det utgår ingen ekonomisk ersättning för deltagande i studien.

Frivillighet

Deltagandet i studien är frivilligt och om Du inte vill vara med kan Du avbryta när som helst under studiens gång utan att behöva förklara varför. Du kommer då att bli erbjuden fortsatt traditionell behandling

Ansvariga

Har du frågor, svarar vi gärna på dem

Studieledare:

Doktor Torsten Olbers,

Telefon: 031 - 3428347 E-post: torsten.olbers@gu.se

Astrid Lindgrens Barnsjukhus

Doktor Annika Janson,

Telefon: Telefon: 0707-375147 E-post: annika.janson@karolinska.se

Koordinator Kerstin Ekbom,

Telefon: 08-58587334 E-post: kerstin.ekbom@karolinska.se

Drottning Silvias Barn och Ungdomssjukhus

Doktor Jovanna Dahlgren,

Telefon: 0702-750233 E-post: jovanna.dahlgren@vgregion.se

Koordinator Eva Gronowitz,

Telefon: 031-3434690 E-post: eva.gronowitz@vgregion.se

Amendments to the protocol

Translated amendment I to the ethical review board

Addendum application regarding the project: Optimized treatment of severe obesity in young people between 12-18 years of age

Dnr: 578-13, Approved by the Regional Ethics Review Board in Gothenburg 2014-01-16.

With this addendum, we apply to raise the inclusion age in the above study to include young people who are also 16 years old (i.e., under 17 years, previously under 16 years). The background to this is that during the first year (2014) it has proved difficult to find enough patients for this RCT. The reason has been, among other things, a marginally too low BMI (1-2 units), final exams before high school in year 9 when they are 15 years old.

The steering group (which includes a paediatrician, surgeon, psychologist, and children's nurse) assesses that this adjustment of the age range for inclusion does not in any way affect the basic ethical issues of the study, but that it would significantly facilitate a faster inclusion of participants.

Addition of cognitive tests in the study:

Research has recently shown an improvement in cognitive functions (such as memory, attention, and executive functions) in adults associated with weight loss after bariatric surgery (1). In young people, there seems to be a connection between abdominal obesity (and poorer executive functions (2) and young people seeking to undergo bariatric surgery report high school-related difficulties (3).

With the addition of cognitive tests in the study, we want to investigate whether there is a connection between weight loss after bariatric surgery and improvement of cognitive functions in young people. It is of high clinical relevance as the vast majority of young people who may be subject to bariatric surgery are still in school. Improved cognitive abilities during adolescence could contribute to improved school success.

We wish to add a customized computerized test battery based on established standard tests that measure executive function (working memory, processing speed as well as cognitive interference and flexibility), visuo-spatial memory and verbal memory. Each test session takes about 30 minutes and is carried out at Baseline and after 2 and 6 months and one-, and two years post op. Data is stored in anonymized form in locked premises and protected data files by being linked to a code number to be able to follow individual development. The code key is also kept by the study leader and the research nurse responsible for the study in locked rooms.

Risks to the individual: There is over a hundred years of experience in the assessment of cognitive abilities in children, adolescents and adults and there are no known serious risks of undergoing cognitive tests. Possibly, the test subject may experience a momentary stress when he/she experiences having his/her abilities assessed or alternatively experience a certain degree of boredom.

Sincerely,

Torsten Olbers, Docent, Senior Consultant in Surgery and chief researcher

Surgical Clinic, Sahlgrenska University Hospital, 41345 Gothenburg

Amendment I to the ethical review board

Addendum-ansökan avseende projektet: *Optimerad behandling av svår fetma hos ungdomar mellan 12-18 år.*

Dnr: 578-13, Godkänt av Regionala etikprövningsnämnden i Göteborg 2014-01-16.

Vi ansöker med detta addendum om att få höja inklusionsåldern i ovanstående studie till att innefatta ungdomar som också är 16 år (d v s under 17 år, tidigare under 16 år). Bakgrunden till detta är att under första året (2014) har det visat sig svårt att finna tillräckligt många patienter för denna RCT. Orsaken har bland annat varit marginellt för låga BMI (1-2 enheter), slutprov inför gymnasiet i 9an när de är 15 år.

Styrgruppen (som innefattar barnläkare, kirurg, psykolog och barnsjuksköterska) bedömer att denna justering av åldersintervallet för inklusion inte på något sätt påverkar de grundläggande etiska frågeställningarna i studien, men att det skulle avsevärt underlätta ett snart genomförande.

Tillägg av kognitiva test i studien:

Forskning har nyligen visat på en förbättring av kognitiva funktioner (såsom minne, uppmärksamhet och exekutiva funktioner) hos vuxna associerat med viktnedgång efter fetmakirurgi (1). Hos ungdomar tycks det finnas ett samband mellan bukfetma (och sämre exekutiva funktioner (2) och ungdomar som söker för att genomgå fetmakirurgi rapporterar i hög utsträckning skolrelaterade svårigheter(3).

Vi vill med tillägg av kognitiva test i studien undersöka om det också hos ungdomar finns ett samband mellan viktminskning efter fetmakirurgi och förbättring av kognitiva funktioner. Det är av hög klinisk relevans då den stora majoriteten av ungdomar som kan vara föremål för fetmakirurgi fortfarande går i skolan. Förbättrade kognitiva förmågor under tonåren skulle kunna bidra till förbättrad skolframgång.

Vi önskar lägga till ett skräddarsytt datoriserat testbatteri utifrån etablerade standard-test som mäter exekutiv funktion (arbetsminne, processhastighet samt kognitiv interferens och flexibilitet), viso-spatialt minne och verbalt minne. Varje testtillfälle tar ca 30 min och genomförs vid Baseline och efter 2 och 6 månader samt ett och två år post op. Data lagras i anonymiserad form i låsta lokaler och skyddade datafiler genom att kopplas till ett kodnummer för att kunna följa individuell utveckling. Kodnyckel förvaras likaså av studieledare och studieansvarig forskningssjuksköterska i låsta lokaler.

Risker för individen: Det finns över hundra års erfarenhet av bedömning av kognitiva förmågor hos barn, ungdomar och vuxna och det finns inga kända alvarliga risker med att genomgå kognitiva test. Möjligen kan testpersonen uppleva en momentan stress då han/hon upplever få sina förmågor bedömda alternativt uppleva ett visst mått av uttråkning.

Med vänliga hälsningar,Torsten Olbers, Docent, Överläkare och huvudansvarig forskare

Kirurgiska kliniken, Sahlgrenska Universitetssjukhuset, 41345 Göteborg

1. Alosco ML, Galioto R, Spitznagel MB, Strain G, Devlin M, Cohen R, et al. Cognitive function after bariatric surgery: evidence for improvement 3 years after surgery. Am J Surg. 2014 Jun;207(6):870-876. PubMed PMID: 24119892. Pubmed Central PMCID: PMC3983172. Epub 2013/10/15. eng.

2. Schwartz DH, Leonard G, Perron M, Richer L, Syme C, Veillette S, et al. Visceral fat is associated with lower executive functioning in adolescents. Int J Obes. 2013 10//print;37(10):1336-1343.

3. Freidl EK, Sysko R, Devlin MJ, Zitsman JL, Kaplan SC, Walsh BT. School and cognitive functioning problems in adolescent bariatric surgery candidates. Surg Obes Relat Dis. 2013 Nov-Dec;9(6):991-996. PubMed PMID: 23932993.

Translated specification to Amendment I to the ethical review board

Project title: Randomized study between surgical and medical treatment for adolescents between 13-16 years of age with severe obesity.

D-no: 578-13

Project manager: Torsten Olbers, Surgeon, SU/Sahlgrenska, 413 45 GÖTEBORG

Thank you for your comments regarding the addendum application in the current project. Title has now been changed in that the age has been raised to 16 years compared to the previous 15 years.

Regarding the validation of the cognitive tests that will be performed, they look as follows:

The below test will be used to study the cognitive abilities of the teenagers. All tests are commonly used in clinical practice and research. The tests are chosen on the basis that they have previously been used in studies where cognitive ability was examined in individuals with obesity. Several subtests are taken from the WISC-IV test battery, which is validated and standardized for children and young people aged 6-16. The subtests from the WISC-IV are also available in an adult version (normed and validated from the age of 16) in the WAIS-IV, which will be offered to young people who have reached the age of 16. D-Kefs 8-89 years The partial test will either be administered as a paper-and-pencil test or computerized.

Language ability and cognitive flexibility:

• The Word Fluency subtest from NEPSY – II. The test taker is asked for one minute to reproduce as many items in a given category as she or he can (measures semantic fluency) and as many words as she or he can with a certain initial letter (measures phonological fluency). The NEPSY-II is validated for the 5-16 age group, but similar tests exist for older youth and adults.

• The Vocabulary subtest from the WISC-IV. The test taker is asked to speak about what a word means. The test leader reads out words of increasing difficulty. Measures general word comprehension and verbal concept formation.

Attention, executive functions and working memory:

• The Digit Repetition subtest from the WISC-IV. The test leader reads out numbers that the test taker must reproduce. In the first part, the test taker must reproduce the numbers in the same order as they are read aloud (measures short-term memory). In the second part, the test subject must reproduce them in reverse order (measures working memory).

• Trail making test consists of two parts. In the first part, the test taker must draw lines between circles in ascending numerical order (1-25). In the second part, the test taker must also draw lines between circles, but this time alternate between numbers and letters in ascending order (1-13 and A-L). The test measures psychomotor speed and ability to shift attention.

• The subtest Elithorn's Labyrinths from the WISC-IV integrated. The test taker must draw a line across a specified number of dots in a maze. In addition to visual abilities, measures ability to plan and inhibit impulses.

• Stroop Colour-Word The test subject sees a word in a colour, e.g. red written in another colour e.g. blue. The test subject must indicate which colour the word is written in. Measures ability for verbal interference, i.e. the ability to withhold automatic and irrelevant responses.

Memory:

• Word list The test taker is given a list of 12 words and must then reproduce these words immediately and then with a delay. The test subject is then presented with other words and must then remember which words were on the first list. The test measures ability to learn and remember.

Perceptual function:

• The Matrices subtest from the WISC-IV. The test taker must choose an image, from several suggested, that fits in with other images. Measures logical thinking and abstract problem solving skills.

Process speed:

• The Coding subtest from the WISC-IV. The test taker must copy simple figures and pair them with the correct number according to a code key. Measures i.a. processing speed, working memory and cognitive flexibility.

Sincerely,

Torsten Olbers, Associate Professor

Specification to Amendment I to the ethical review board
Projekttitel: Randomiserad studie mellan kirurgisk och medicinsk behandling för ungdomar mellan 13-16 år med svår fetma.

D-nr: 578-13

Projektansvarig: Torsten Olbers, Kirurgen, SU/Sahlgrenska, 413 45 GÖTEBORG

Hej!

Tack före era kommentarer gällande addendumansökan i aktuellt projekt. Titel har nu ändrats genom att åldern har höjts till 16 år jämfört med tidigare 15 år.

Gällande valideringen av de kognitiva test som kommer att utföras ser de ut som följer:

Nedanstående test kommer att användas för att studera kognitiva förmågor hos tonåringarna. Samtliga test är vanligt förekommande inom klinisk verksamhet och forskning. Testen är valda utifrån att de tidigare är använda i studier där kognitiv förmåga undersökts hos individer med fetma. Flera deltest är hämtade ur testbatteriet WISC-IV, vilket är validerat och normerat för barn och ungdomar 6-16 år. Deltesten från WISC-IV finns också i vuxenversion (normerat och validerat från 16 år) i WAIS-IV, vilket kommer erbjudas till de ungdomar som hunnit fylla 16 år. D-Kefs 8-89 år Deltesten kommer antingen att administreras som papper-och-penna test eller datoriseras.

Språklig förmåga och kognitiv flexibilitet:

- Deltestet Ordflöde från NEPSY – II. Testpersonen ombeds att under en minut återge så många i saker i en given kategori som hon eller han kan (mäter semantiskt flöde) och så många ord hon eller han kan på en viss begynnelsebokstav (mäter fonologiskt flöde). NEPSY-II är validerat för gruppen 5-16 år, men snarlika test finns för äldre ungdomar och vuxna.
- Deltestet Ordförråd från WISC-IV. Testpersonen ombeds att tala om vad ett ord betyder. Testledaren läser upp ord med ökande svårighetsgrad. Mäter allmän ordförståelse och verbal begreppsbildning.

Uppmärksamhet, exekutiva funktioner och arbetsminne:

- Deltestet Sifferrepetition från WISC-IV. Testledaren läser upp siffror som testpersonen ska återge. I första delen ska testpersonen återge siffrorna i samma ordning som de läses upp (mäter korttidsminne). I andra delen ska testpersonen återge dem i omvänd ordning (mäter arbetsminne).
- Trail making test består av två delar. I första delen ska testpersonen dra linjer mellan cirklar i stigande nummerordning (1-25). I den andra delen ska testpersonen också dra linjer mellan cirklar, men denna gång alternera mellan siffror och bokstäver i stigande ordning (1-13 och A-L). Testet mäter psykomotorisk hastighet och förmåga att växla uppmärksamhet.
- Deltestet Elithorns labyrinter från WISC-IV integrerad. Testpersonen ska dra en linje över ett specificerat antal prickar i en labyrint. Mäter utöver visuella förmågor förmåga till planering och inhibition av impulser.
- Stroop Color-Word Testpersonen ser ett ord på en färg t.ex. röd skrivet i en annan färg t.ex. blå. Testpersonen ska ange vilken färg som ordet är skrivet i. Mäter förmåga till verbal interferens, d.v.s. förmågan att hålla tillbaka automatiska och irrelevanta svar.

Minne:

- Ordlista Testpersonen får ta del av en lista på 12 ord och ska sedan återge dessa ord direkt och sedan med fördröjning. Testpersonen blir sedan presenterad för andra ord och ska då komma ihåg vilka ord som fanns på den första listan. Testet mäter förmåga till inlärning och minne.

Perceptuell funktion:

- Deltestet Matriser från WISC-IV. Testpersonen ska välja en bild, av flera föreslagna, som passar in efter andra bilder. Mäter logiskt tänkande och abstrakt problemlösningsförmåga.

Processhastighet:

- Deltestet Kodning från WISC-IV. Testpersonen ska kopiera enkla figurer och para ihop dem med rätt siffra enligt en kodnyckel. Mäter bl.a. bearbetningshastighet, arbetsminne och kognitiv flexibilitet.

Med vänliga hälsningar

Torsten Olbers, docent.

Translated amendment application II in AMOS2
(AMOS1 Marked in yellow, N/A)

Application for ethics review, Application for change

1.1. -Enter the diary number of the previously approved basic application.

- Dnr 578-13 (AMOS2)

- T305-15, AD578-13 (AMOS2)

- Dnr 523-04 (AMOS1)

- Dnr 2019-00769/1163-18 (AMOS1). N/A

1.2. Enter the responsible researcher for the previously approved basic application.

Torsten Olbers

1.3. **Enter the research principal for the previously approved basic application.**

Västra Götaland Region, Sahlgrenska University Hospital, Surgery Sahlgrenska

The studies are currently run by:

Västra Götaland Region, Queen Silvia's Children and Youth Hospital, Children's Medicine

- Regional obesity centre for children and young people.

1.4. Enter the title of the previously approved basic application.

- Randomized trial between surgical and medical treatment for adolescents between 13 and 16 years of age with severe obesity

- Addendum to above for Cognitive test

- Study of surgical intervention to treat severe obesity in young people aged 13 to 18 years

1.5. Briefly describe the change to the previously approved application that is planned.

We wish for the 5 and 10 year follow-up in AMOS 2 (578-13):

1. Cognitive function test up to 2 years is ethically approved (T305-15, AD578-13). Now wish to do the same cognitive test also at 5 and 10 years to study about lasting differences.

2. In-depth interview of the young people and parents was approved for AMOS 1; we wish to do this in AMOS 2 as well.

3. Analyse Blood-Peth (specific blood marker that reflects alcohol consumption in recent weeks) in connection with regular blood sampling in AMOS2. Little or no extra blood. This analysis is previously granted for AMOS1 523-04.

4. We wish to add adult forms for ADHD and Autism screening

o ADHD-RS

o AQ10

The questionnaires are attached, including those previously approved for AMOS1 (523-04).

5. Conduct in-depth interviews with the young people and the guardians in connection with the five-year follow-up.

1.6. State the reasons underlying the planned change.

1. The cognitive tests have worked well; we now wish to follow outcomes also during longer follow-up.

2. Overall, we have seen in both surgery projects that there is a need for young people but also their parents to discuss how they experienced the journey with expectations and concerns and more. Turned out well in the first AMOS, so we now wish to do it in AMOS 2 as well.

3. Added now as the study participants were previously very young.

4. Earlier surveys were based on information from the parents, now they can be done by the young people themselves.

5. The reason for conducting semi-structured in-depth interviews is to supplement and deepen the evidence regarding young people who have undergone surgery as a treatment due to morbid obesity. Data will be collected through a qualitative semi-structured in-depth interview on one occasion. The qualitative method is well suited when a subject is unexplored because the richness of detail in the data collection contributes to a deeper and more detailed knowledge. This, in turn, can increase the understanding of a phenomenon, for example how patients experience different parts of the care, and their experiences around a treatment.

1.7. Assess how the relationship between the risks and benefits of the project changes due to the planned change.

We see no risks with the proposed changes.

- Regarding the questionnaires, we will remove some from previous study visits and add these two so the time required will be approximately the same

- Cognitive testing has already been done on three occasions during the study so the participants are well familiar with it

- B-Peth is analysed partly to map possible differences in the study groups but also to catch incipient overconsumption early (increased risk of overconsumption of alcohol after surgery, therefore in many places the sample is taken on a clinical basis). Feedback is given to the study participants about if elevated and discuss through.

- We do not believe that the written information to the research subjects needs to be changed due to the planned change, but in connection with the examination day it can be discussed with study staff.

1.8. If applicable, describe how other information/appendices change due to the planned change.

No changes.

1.9. If applicable, indicate which attachments are attached to the application.

Surveys:

ADHD-RS

AQ10

____________________________________ _______________________________________

Underskrift ansvarig forskare (eller behörig företrädare för forskningshuvudman)

Torsten Olbers, Professor och överläkare DSBUS   Younis Khalid, verksamhetschef, Barnmedicin

Amendment application II in AMOS2

Ansökan om etikprövning

Ansökan om ändring

1.1. -Ange diarienummer på den tidigare godkända grundansökan.

- Dnr 578-13 (AMOS2)
- T305-15, AD578-13 (AMOS2)

- *Dnr 523-04 (AMOS1)*
- *Dnr 2019-00769/1163-18 (AMOS1)*

1.2. Ange ansvarig forskare för den tidigare godkända grundansökan.

Torsten Olbers

1.3. Ange forskningshuvudman för den tidigare godkända grundansökan.

Västra Götalandsregionen, Sahlgrenska Universitetssjukhuset, Kirurgi Sahlgrenska

Studierna drivs f n genom:

Västra Götalandsregionen, Drottning Silvias Barn och Ungdomssjukhus, Barnmedicin

- Regionalt obesitascentrum för barn och ungdomar

1.4. Ange titel på den tidigare godkända grundansökan.

- **Randomiserad studie mellan kirurgisk och medicinsk behandling för ungdomar mellan 13 till 16 år med svår fetma**
- **Addendum till ovan för Kognitivt test**
- *Kirurgisk interventions studie mot svår övervikt hos ungdomar 13 till 18 år*
- *Addendum: Att genomgå överviktskirurgi: från patient- och föräldraperspektiv (kvalitativ del)*
  Max 500 tecken

1.5. Beskriv kortfattat den ändring av tidigare godkänd ansökan som planeras.

Vi önskar för 5 och 10 årsuppföljningen i AMOS 2 (578-13):

1. Kognitivt funktionstest upp till 2 år är etikgodkänt (T305-15, AD578-13). Önskar nu göra samma kognitiva test även vid 5 och 10 år för att studera om bestående skillnader.

2. Djupintervju av ungdomarna och föräldrar godkändes för AMOS 1; vi önskar göra detta även i AMOS 2.

3. Analysera Blod-Peth (specifik blodmarkör som speglar alkoholkonsumtion senaste veckorna) i samband med ordinarie blodprovstagning. Obetydlig eller ingen mängd extra blod. Denna analys är tidigare beviljad för AMOS1 523-04.

4. Vi önskar lägga till vuxenformulär för ADHD och Autism-screening

   - ADHD-RS
   - AQ10

Enkäterna bifogas, även de tidigare godkända för AMOS1 (523-04).

5. Utföra djupintervjuer med ungdomarna och vårdnadshavarna i samband med femårsuppföljningen.

1.6. Ange de skäl som ligger till grund för den planerade ändringen.

1. Kognitiva testerna har funkat bra, vi önskar nu följa utfall även under längre tids uppföljning.

2. Övergripande har vi i de båda kirurgiprojekten sett att det för ungdomar men också deras föräldrar finns ett behov att få diskutera hur man upplevt resan med förväntningar och oro med mera. Föll väl ut i första AMOS, därför önskar vi nu göra även i AMOS 2.

3. Läggs till nu då studiedeltagarna tidigare varit mycket unga.

4. Tidigare enkäter byggde på information från föräldrarna, nu kan de göras av ungdomarna själva.

5. Skälet till att genomföra semistrukturerade djupintervjuer är att komplettera och fördjupa evidensen kring ungdomar som genomgått kirurgi som behandling på grund av sjuklig fetma. Data kommer att samlas in genom en kvalitativ semistrukturerade djupintervju vid ett tillfälle. Den kvalitativa metoden lämpar sig väl när ett ämne är outforskat eftersom detaljrikedomen i datainsamlingen bidra med en djupare och mer detaljerad kunskap. Detta kan i sin tur öka förståelsen för ett fenomen, exempelvis hur patienter upplever olika delar av vården, och deras erfarenheter kring en behandling.

1.7. Gör en värdering av hur förhållandet mellan riskerna och nyttan av projektet förändras med anledning av den planerade ändringen.

Vi ser inga risker med föreslagna förändringar.

- Vad gäller enkäterna kommer vi att ta bort några från tidigare studiebesök och lägga till dessa två så tidsåtgången kommer bli ungefär detsamma
- Kognitivt test har redan gjorts vid tre tillfällen under studien så deltagarna är väl bekanta med den
- B-Peth analyseras dels för att kartlägga ev. skillnader i studiegrupperna men också för att fånga begynnande överkonsumtion tidigt (ökad risk för överkonsumtion av alkohol efter kirurgi, därför tas på många håll provet på klinisk bas). Feedback ges till studiedeltagarna om förhöjt, och diskuterar igenom.
- Vi anser inte att den skriftliga informationen till forskningspersonerna behöver förändras med anledning av den planerade ändringen, men i samband med undersökningsdag kan det diskuteras med studiepersonal.

1.8. Beskriv i förekommande fall hur annan information/bilagor förändras med anledning av den planerade ändringen.

Inga ändringar.

1.9. Ange i förekommande fall vilka bilagor som bifogas ansökan.

Enkäter:

ADHD-RS

AQ10

I och med att ansökan undertecknas intygar du som är ansvarig forskare följande;

Att den information som lämnas i ansökan om etikprövning och samtliga medföljande bilagor är riktig och fullständig.

Att verksamhetsansvariga i samtliga medverkande verksamheter är informerade om forskningsprojektets innehåll och utförande och att de har samtyckt till att delta i studien.

Att du säkerställt att det i samtliga medverkande verksamheter finns resurser som garanterar forskningspersonernas säkerhet och integritet vid genomförandet av den forskning som beskrivs i ansökan.

Att du tagit del av Etikprövningsmyndighetens information om hantering av personuppgifter på myndighetens webbplats.

Underskrift ansvarig forskare (eller behörig företrädare för forskningshuvudman)

Torsten Olbers, Professor och överläkare<br>DSBUS<br>2020-05-14	Younis Khalid, verksamhetschef, Barnmedicin

Summary of changes to the protocol

Expansion of inclusion criteria

Due to slow recruitment, the original age range 13 to 15 years, was expanded to 13 to 16 years.

Change of time frame

Originally, the time points for follow-up for most of the outcomes were planned at 2, 7, 12, and 17 years after inclusion. This was changed to 2, 5, 10, and 15 years in order to match registration in the national quality of care register SOReg (Scandinavian Obesity surgery Register).

In the original protocol, the primary end point (BMI) was planned to be analysed at all follow-ups (2, 7, 12, and 17 years). The primary end point is now analysed at the 2-year follow-up only.

Expansion of including centres

At the start, there were 2 recruitment sites. Due to slow recruitment a third centre joined the study after 9 months.

Expansion of data collection

- Assessment of cognitive functioning (IQ, cognitive flexibility, processing speed, memory, attention, working memory, and executive functioning) at inclusion and follow-ups.
- Added questionnaires: the ADHD Rating Scale (ADHD-RS) and Autism Spectrum Quotient (AQ10). These are self-report questionnaires assessing neuropsychiatric symptoms and are administrated from the 5-year follow-up and beyond. Neuropsychiatric symptoms were parent-reported at baseline.
- Analyse of Phosphatidylethanol in blood (B-PEth) at follow-ups. B-PEth is a marker for alcohol use and abuse.
- Qualitative interviews with participants and their parents at the 5-year follow-up in order to get a better understanding of the experience of undergoing surgical and non-surgical treatment as a teenager with severe obesity and how the parents experience the process.

Exclusion of planned data collection

- Endothelial function measurements- Endothelial function will be assessed using reactive hyperaemia peripheral and arterial tonometry (pulse wave velocity)
- Ultrasound measurements with high-resolution ultrasound at 55 MHz (Visualsonics)
- 24-hour blood pressure measurement. Sleep examinations in the home environment.
- Addictive behaviour at 2 and 5 years is not assessed with brain response to visual stimuli (not in ethical application, only listed on Clinical trials).
- Presence of OCD (obsessive compulsive disorder) is not assessed at 2 and 5 years (not in ethical application, only listed on Clinical trials).
- Assessment of energy expenditure at 5 years after treatment initiation with doubly labelled water, basic metabolic rate, and 24h energy expenditure chamber is omitted (not in ethical application, only listed on Clinical trials).

Statistical analysis plan

The Adolescent Morbid Obesity Surgery 2 (AMOS2): Cognitive function

Clinical trials identifier: NCT02378259

Principal Investigator: Torsten Olbers, MD, PhD, Linköping University, Department of Biomedical and Clinical Sciences and Wallenberg Centre for Molecular Medicine

Created: 2019-12-13 by Markku Peltonen, PhD

Updated: 2023-07-26

1. Study objectives

The trial is an intervention in adolescents with severe obesity, aiming to achieve weight loss. It aims to evaluate whether it is beneficial to undergo bariatric surgery in early adolescence, as compared to intensive non-surgical treatment for obesity. In addition to the primary outcome of change in body mass index (BMI), it also aims to evaluate a number of secondary outcomes such as development of cardiovascular illness and cancer, biochemical markers of metabolic health, body composition, bone health, physical fitness, quality of life, and psychological and cognitive functioning. This statistical analysis plan considers cognitive functioning, which is one group of the secondary outcomes.

The study was registered at www.clinicalTrials.gov; trial number NCT02378259.

2. Study Design

2.1 Overview

The study is an investigator-initiated, randomized, parallel group, multicentre study with 50 adolescents with severe obesity, aged 13–16 years old, randomised to either bariatric surgery or intensive non-surgical obesity treatment. Three paediatric obesity clinics in Sweden collaborated in designing the study and in the recruitment of patients from 1 August 2014 to 30 June 2017. Patients were selected among adolescents 13-16 years of age who had been in obesity care for at least one year and treated at one of the including clinics for at least six months. The follow-up time for the primary outcome reporting is 2 years.

2.2 Randomization

Patients were randomized in parallel 1:1 to bariatric surgery (predominantly Roux-en-Y gastric bypass) or intensive non-surgical treatment starting with an eight-week low-calorie-diet. Randomization was computerized and stratified for treatment and sex within each study centre. After the inclusion visit, the treatment allocation was not blinded.

2.3 Eligibility criteria

Inclusion Criteria:

- Age 13-16 years
- BMI ≥35
- Failed comprehensive treatment for obesity >1 year
- Approval following assessment by Psychologist
- Tanner pubertal stage ≥3

Exclusion Criteria:

- Monogenic obesity (e.g. Prader Willi, Laurence Moon-Bardet-Biedl)
- Obesity secondary to brain injury
- Severe mental disability
- Unsuitable for general anaesthesia
- Psychotic or other major psychiatric illness
- Previous major gastrointestinal surgery

Detailed inclusion and exclusion criteria are provided in the study protocol [1].

2.4 Data collection and follow-up

Assessments are scheduled at 2 months (weight only), and 1, 2, 5, 10, and 15 years from baseline, including team-visits for assessment of secondary outcome measures. All relevant demographic and medical data, including outcome measures were collected in case report forms (CRF). The study coordinators at each site

reported data to the central coordinator who entered the data in the CRF. Surgical adverse events are retrieved from the Scandinavian Obesity Surgery Registry.

In addition, data from mandatory national registries for assessment of health care consumption and socioeconomic development will be collected. We will also assess how many patients in the non-surgical treatment group that chose to undergo bariatric surgery later, as well as long-term cardiovascular events, cancer incidence, and overall mortality.

3. Study outcomes

Primary outcome

The primary outcome of the study is the difference in changes in BMI over two years between the surgical and non-surgical treatment arms.

Secondary outcome measures

The secondary outcomes will be differences in the development of cardiovascular illness and cancer, biochemical markers of metabolic health, body composition, bone health, physical fitness, quality of life, and psychological and cognitive functioning. All the secondary outcomes to be reported are listed in the appendix.

In addition to the primary reporting after 2-year follow-up, both the primary and secondary outcomes will also be analysed for 5-, 10- and 15-year changes.

This statistical analysis plan considers specifically variables related to the cognitive functioning. The variables include: estimated IQ (Vocabulary, Matrix Reasoning, Digit Span, and Coding), Verbal memory (Immediate and Delayed recall), Verbal fluency (Letter and Category Fluency, and Category Switching), Trail Making Test (Visual scanning, Number and Letter Sequencing, Number-Letter Switching, and Motor Speed), Colour-Word Interference Test (Colour Naming, Word Reading, Inhibition, and Inhibition/Switching) and Mazes at inclusion and follow-ups at 1- and 2-year.

4. Statistical methods

4.1 Sample size and power

For the sample size calculation for the primary outcome, we assumed a 2-year reduction in BMI of 15 kg/m^2 in the surgery group based on the results of the AMOS-study [2] with a standard deviation (SD) 7 kg/m^2. Further, we assumed a reduction in BMI of 5 kg/m^2 in the intensive non-surgical treatment group with SD 7 kg/m^2. Based on these assumptions, a total sample size of 50 (25 in each group) would provide >95% power at 0.01 significance level to demonstrate a difference in BMI-change over two years between the groups.

For cognitive functioning, no formal power calculation was made. Therefore, all the analyses will be regarded explorative in publications.

4.2 Principles of analysis

The primary conclusions from this study will be based on analyses conducted under the principle of intention-to-treat. Thus, all randomized patients will be included in the analyses, and it is assumed that all randomized patients receive the treatment for which they were randomized.

The difference in BMI-changes between the groups will be estimated with a multilevel mixed-effect regression model utilizing BMI measurements at all available time points. This model considers the repeated measurements nested within persons over time. All randomized patients except those who withdraw their consent will be included in this analysis.

4.3 Statistical analyses

Summary tables (descriptive statistics and frequency tables) will be provided for all baseline variables, outcome variables and safety variables. Continuous variables will be summarized with descriptive statistics (n, mean, standard deviation, range and median). Frequency counts and percentage of subjects within each category will be provided for categorical data. The main conclusions of the study on primary outcome will be based on the estimated difference between 2-year changes in BMI in the surgery and intensive non-surgical treatment groups.

The differences between treatment groups on cognitive function will be estimated with a multilevel mixed effect regression model, adjusted for the stratifying variables (sex, study centre) and for the baseline level of the respective cognitive functioning variable. Two-sided p-values <0.05 will be considered statistically significant.

No correction for the significance level will be done due to multiple testing nor multiple confidence intervals, but all the prespecified secondary outcomes to be analysed will also be reported.

4.4 Missing data

For evaluation of primary outcome, it is expected that the coverage will be close 100%. All patients except those who withdraw consent will be included in the analyses. In the case data for the outcome is missing from all follow-up examinations, a sensitivity analysis with multiple imputation will be conducted and reported.

5. References

1. Janson A, Järvholm K, Gronowitz E, et al. A randomized controlled trial comparing intensive non-surgical treatment with bariatric surgery in adolescents aged 13–16 years (AMOS2): Rationale, study design, and patient recruitment. Contemporary Clinical Trials Communications 2020;19:100592. DOI: 10.1016/j.conctc.2020.100592.

2. Olbers T, Beamish AJ, Gronowitz E, et al. Laparoscopic Roux-en-Y gastric bypass in adolescents with severe obesity (AMOS): a prospective, 5-year, Swedish nationwide study. Lancet Diabetes Endocrinol 2017;5(3):174-183. DOI: 10.1016/S2213-8587(16)30424-7.

6. Appendix

Outcomes to be analysed:

**Anthropometrics**	
	Height - m
	Body Weight - kg
	BMI
	BMI - standard deviation score
	Waist circumference — cm
	Hip circumference — cm
**Glucose control**	
	HbA1c (mmol/mol)
	Fasting plasma glucose (mmol/L)
	Fasting plasma insulin (pmol/L)
	Glucose after oral glucose tolerance test
**Blood lipids**	
	Triglycerides (mmol/L)
	LDL cholesterol (mmol/L)
	HDL cholesterol (mmol/L)
**Blood pressure (mm Hg)**	
	Systolic
	Diastolic
**Inflammation**	
	High-sensitivity C-reactive protein (mg/L)
**Liver function**	
	Alanine aminotransferase (µkat/L)

Aspartate aminotransferase (µkat/L)
**Bone health**
DXA z-score total
**Quality of Life**
Physical functioning
Physical role functioning
General health perceptions
Bodily pain
Vitality
Mental health
Social role functioning
Emotional role functioning
Obesity-related problems
**Mental health**
Anxiety symptoms
Depressive symptoms
Anger symptoms
Disruptive behaviour
Self-esteem
**Eating related problems**
Binge eating
Uncontrolled eating
Emotional eating
Cognitive restraint
**Risk taking behaviour**
AUDIT
Phosphatidylethanol in blood
DUDIT
Smoking
**Cognitive functioning**
IQ (Vocabulary, Matrix Reasoning, Digit Span, and Coding)
Verbal memory (Immediate and Delayed recall)
Verbal fluency (Letter and Category Fluency, and Category Switching)
Trail Making Test (Visual scanning, Number and Letter Sequencing, Number-Letter Switching, and Motor Speed)
Colour-Word Interference Test (Colour Naming, Word Reading, Inhibition, and Inhibition/Switching)
Mazes
